# The Human Cardiac “Age‐OME”: Age‐Specific Changes in Myocardial Molecular Expression

**DOI:** 10.1111/acel.70219

**Published:** 2025-09-07

**Authors:** Cassandra Malecki, Giovanni Guglielmi, Benjamin Hunter, Dylan Harney, Yen Chin Koay, Anthony. S. Don, Oscar Han, Jasmine Khor, Lisa Nguyen, Michael Pan, Peter Gawthrop, Nathan Isles, Joshua Chung, Robert. D. Hume, Matthew Taper, XiaoSuo Wang, Mark Larance, Fabian Spill, Vijay Rajagopal, John F. O’Sullivan, Sean Lal

**Affiliations:** ^1^ School of Medical Sciences, Faculty of Medicine and Health The University of Sydney Sydney New South Wales Australia; ^2^ The Baird Institute for Applied Lung and Heart Research Sydney New South Wales Australia; ^3^ Department of Biomedical Engineering The University of Melbourne Melbourne Victoria Australia; ^4^ School of Mathematics University of Birmingham Birmingham UK; ^5^ Chris O’Brien Lifehouse Hospital Sydney New South Wales Australia; ^6^ School of Mathematics and Statistics, Faculty of Science The University of Melbourne Melbourne Victoria Australia; ^7^ Department of Cardiovascular Sciences KU Leuven Leuven Belgium; ^8^ Alan Turing Institute London UK; ^9^ Baker Department of Cardiometabolic Health, Melbourne Medical School The University of Melbourne Melbourne Victoria Australia; ^10^ The Graeme Clark Institute University of Melbourne Parkville Victoria Australia; ^11^ Department of Cardiology Royal Prince Alfred Hospital Sydney New South Wales Australia

**Keywords:** ageing, age‐OME, excitation‐contraction coupling, human heart, metabolism, omics

## Abstract

Ageing is one of the most significant risk factors for heart disease; however, it is still not clear how the human heart changes with age. Taking advantage of a unique set of pre‐mortem, cryopreserved, non‐diseased human hearts, we performed omics analyses (transcriptomics, proteomics, metabolomics, and lipidomics), coupled with biologically informed computational modelling in younger (≤ 25 years old) and older hearts (≥ 50 years old) to describe the molecular landscape of human cardiac ageing. In older hearts, we observed a downregulation of proteins involved in calcium signalling and the contractile apparatus. Furthermore, we found a potential dysregulation of central carbon generation of fuel, glycolysis, and fatty acids oxidation, along with an increase in long‐chain fatty acids. This study presents and analyses the first molecular data set of normal human cardiac ageing, which has relevant implications for understanding the human cardiac ageing process and the development of age‐related heart disease.

## Graphical abstract

A multi‐omics analysis was performed to uncover the molecular landscape of human cardiac ageing. We report several insights for the first time: a decrease in proteins composing the structural machinery of the heart; a likely compensatory increase in energetic flux and redox reductive capacity; and an increase in lysosomal activity.

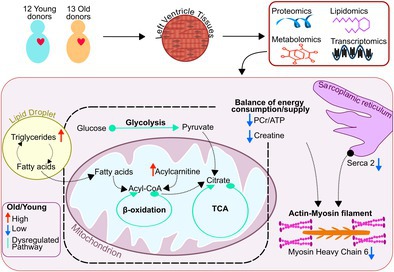

## 1 Introduction

The world's population is rapidly ageing, with older age being a significant contributor to the burden of diseases (Bosch et al. 2019; Ciumărnean et al. 2021; Yazdanyar and Newman 2009). Older age is a strong risk factor for the development of cardiovascular disease (CVD), including heart failure (HF) (Paterson 2011). However, our understanding of the underlying mechanisms that contribute to age‐related risk of CVD in humans is incomplete.

Human cardiac ageing leads to changes in the cardiac cycle, with the prevalence of diastolic dysfunction significantly increasing after 50 years of age (Huynh et al. 2006). There is a profound lack of therapies for diastolic HF; hence, a clear understanding of normal versus pathological human myocardial ageing is needed. Cardiac studies of ageing to date rely on animal models, which have limitations in translatability to the human heart (Anker et al. 2021).

The present study harnesses a unique collection of cryopreserved heart tissues across the human lifespan that were acquired pre‐mortem from heart transplant donors (Lal et al. 2015). Samples were stratified into “younger” (age ≤ 25) and “older” (age ≥ 50) cohorts, where the latter criterion was based upon the increased epidemiological prevalence of CVD above the age of 50 years (Foreman et al. 2018). The resulting molecular resource includes measurement of proteins, metabolites, lipids, and transcripts in donor hearts that depict changes occurring during the human cardiac ageing process. Functional implications of key molecular changes were further investigated using biophysics‐based and physiology‐informed in silico models. The results of the omics analyses are available publicly on an interactive platform (https://humancardiac.shinyapps.io/ageing/).

## 2 Methods

Detailed methods including detailed statistical methods are provided as part of the Supporting Information.

### 2.1 Human Heart Tissue

Control donor hearts were collected in the 1990s/early 2000s at St Vincent's Hospital, Sydney, Australia, the only heart transplant center in the country at the time. The hearts were deemed suitable for heart transplantation but unable to be transplanted (reasons including transportation logistics, immune incompatibility, and donor‐recipient mismatch in size) and were procured as previously described (Lal et al. 2015; Li et al. 2020; Mollova et al. 2013; Polizzotti et al. 2015). These hearts were from patients who had been placed on life support for a period of time, following a catastrophic event and who had then subsequently been declared brain dead due to a non‐cardiac cause of death. Therefore, they are not post‐mortem samples. These patients had no medical history of cardiac disease. Additionally, to be considered as heart donors, no cardiac risk factors were present in any patients (BMI < 30, non‐smokers, no diabetes, no hypertension). Furthermore, the donors did not carry any chronic illnesses. The hearts underwent formal pathological examination and were deemed structurally normal after histological analysis. LV samples were obtained immediately in the operating theatre and were snap frozen in liquid nitrogen (−196°C) and subsequently stored in the Sydney Heart Bank located at The University of Sydney at −170°C to −180°C. The study was approved by the Ethics Committee of The University of Sydney (USYD # 2021/122).

## 3 Results

### 3.1 Donor Characteristics

**FIGURE 1:**
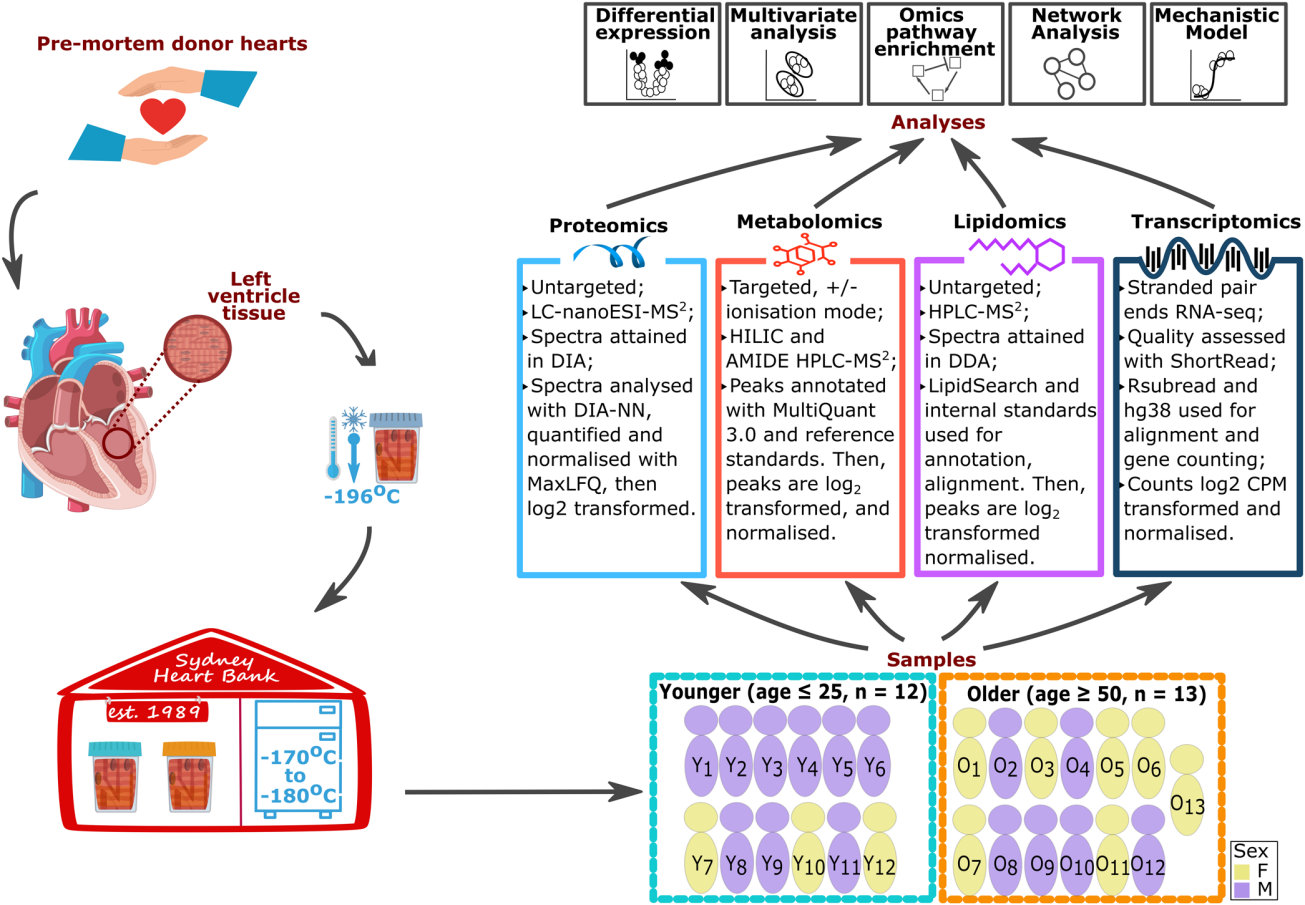
Study design. Left ventricular tissue was acquired from pre‐mortem healthy donor hearts as classified by formal pathological examination and stored at the Sydney Heart Bank. Donors' tissues were selected based on tissue availability (*n*_younger_ = 12, *n*_older_ = 13), and utilised for omics and computational analyses.

The clinical information of each donor in the study is summarised in Table S1. The younger cohort's age ranges from 14 to 25 years old (*n* = 12, mean ± SD age, 19 ± 3.4), and the older cohort ranges from 51 to 65 years old (*n* = 13, mean ± SD age, 57 ± 4.7). Although there was a qualitative difference in sex distribution between the groups, with six males in the older cohort and nine males in the younger cohort (Figure 1), a quantitative assessment through the test of equal proportion did not show any strong evidence of imbalance due to sex in the two cohorts (*P*_val_ = 0.2881). The qualitative asymmetric distribution of the sexes among the younger donors was mainly due to risk‐taking activities that young males exposed themselves to, which led to fatal accidents, such as motor vehicle accidents. The older donor cohort contain more females, who are less at risk of CVD than males yet more likely to suffer brain death due to non‐cardiac‐related pathologies, such as brain aneurysms.

### 3.2 Proteomics of Younger Versus Older Hearts

**FIGURE 2:**
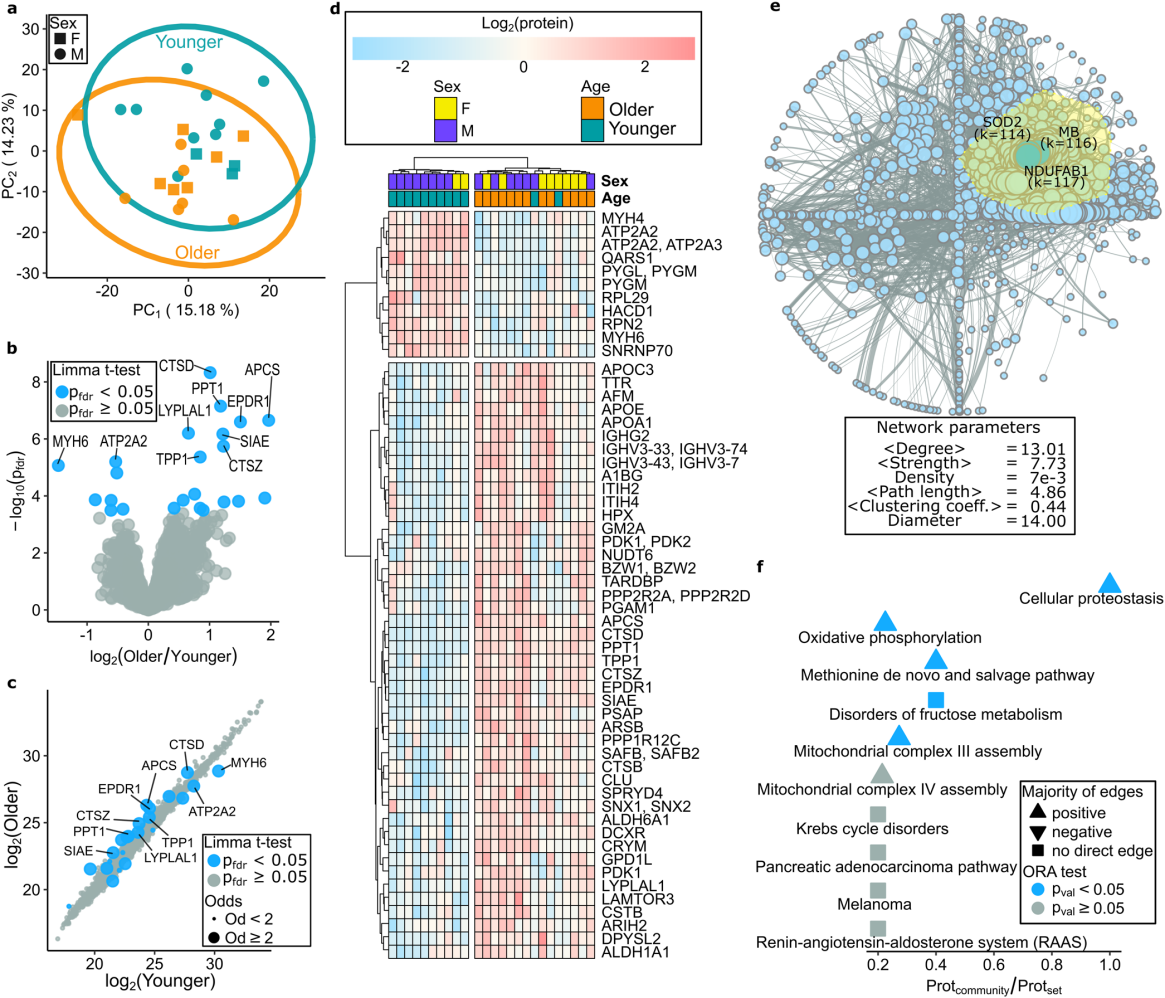
Analysis of protein abundances of younger and older hearts. (a) PCA of younger and older hearts. (c) Volcano plot of protein changes between younger and older hearts. (c) Scatter plot of log_2_ protein abundances of the younger and older cohorts. (d) Heatmap with hierarchical clustering of samples and proteins with *P*_fdr_ < 0.1. (e) WCNA. The nodes, proteins, have radius proportional to their degree, and edges are proportional to the abs(correlation) between proteins. (f) Overrepresentation analysis (ORA) of pathways in the largest protein community. An up/down‐pointing triangle indicates most correlations are positive/negative, and a square is used when proteins within a pathway are not directly correlated.

Untargeted proteomic analysis was performed on human left ventricle (LV) myocardial tissue samples of the younger cohort (*n* = 12) and the older cohort (*n* = 13). A total of 2938 proteins were analyzed. Principal component analysis (PCA) was performed to examine donors within each cohort. In each cohort, the donors show evidence of agreement and no specific clustering based on sex (Figure 2a). Comparison between the two groups identified 56 proteins with FDR corrected *p*‐value below 0.1 (*P*_fdr_ < 0.1), of which 23 proteins exhibit *P*_fdr_ < 0.05 (Figure 2b–d, Table S2). We will refer to the latter as differentially expressed (DE) proteins. A heat map summarizing the protein abundances with associated *P*_fdr_ < 0.1 is presented in Figure 2d, with unsupervised hierarchical clustering showing a distinction between the younger and older cohorts, except for only two samples.

The protein that exhibited the strongest evidence of increased expression in the older hearts was cathepsin D (*CTSD*) (FC = 2.01, *P*_fdr_ = 1.39 × 10^−5^), a member of the family of cathepsin proteases, which are major components of the lysosomal proteolytic system (O'Toole et al. 2020). Cathepsin Z (*CTSZ*) (FC = 2.34, *P*_fdr_ = 7.56 × 10^−4^) and cathepsin B (*CTSB*) (FC = 1.69, *P*_fdr_ = 2.1 × 10^−2^) were also increased in older hearts. There was also an increase in other lysosomal proteins, including palmitoyl‐protein thioesterase 1 (*PPT1*) (FC = 2.27, *P*_fdr_ = 1.04 × 10^−4^), which removes long‐chain fatty acids (LCFAs) from specific proteins (assists with protein breakdown) (Lu et al. 2002), tripeptidyl peptidase 1 (*TPP1*) (FC = 1.80, *P*_fdr_ = 1.59 × 10^−3^) and arylsulfatase B (*ARSB*) (FC = 1.80, *P*_fdr_ = 4.0 × 10^−2^), involved in the breakdown of glycosaminoglycans (Pohl et al. 2018).

Upregulation of apolipoprotein E (*APOE*) (FC = 2.36, *P*_fdr_ = 2.7 × 10^−2^) and apolipoprotein C3, *APOC3*, (FC = 2.78, *P*_fdr_ = 2.7 × 10^−2^) was also observed in the older hearts. Both proteins are involved in cholesterol metabolism and triglyceride homeostasis through the formation of lipoproteins (Sacks 2015).

The two proteins with the most substantial evidence of downregulation in the older cohort are essential in cardiac contraction and relaxation. The first is sarcoendoplasmic reticulum Ca^2+^‐ATPase 2, *SERCA2*, or *ATP2A2* (FC = 0.69, *P*_fdr_ = 2.06 × 10^−3^), which is located on the sarcoplasmic reticulum (SR) membrane and regulates Ca^2+^ re‐uptake from the cytosol into the SR during diastole, ready to be released from the SR during the next cycle of contraction (Eisner et al. 2020). Immunostaining of *SERCA2* in a subset of young and older LV tissue also highlighted a significant decrease in *SERCA2* in older hearts (Figure S1). The second most significantly decreased DE protein in older hearts was cardiac alpha (α)‐myosin heavy chain (*αMyHC*), or *MYH6* (FC = 0.36, *P*_fdr_ = 2.51 × 10^−3^), a core component of type II myosin which generates fast mechanical force during contraction (Miyata et al. 2000). Immunostaining of *MYH6* showed a significant decrease in *MYH6* in older hearts, supporting the proteomics analysis (Figure S1). Furthermore, myosin heavy chain 4 (*MYH4*) decreased in the older cohort (FC = 0.75, *P*_fdr_ = 4.0 × 10^−2^).

To assess sex‐specific changes, additional analysis of the proteomics data was performed, comparing younger females versus older females and younger males versus older males. An important caveat to these analyses is the low sample size per sex (3 younger females vs. 7 old females; 9 younger males vs. 5 older males) and therefore adjusting for multiple comparisons was not statistically robust. For this reason, nominal *p* values were considered when interpreting the data (Table S3). A list of proteins that satisfy one of the following criteria were compiled: *p*_fdr_ < 0.1 in the overall ageing analysis, *p*_val_ < 0.05 in the ageing female analysis, or *p*_val_ < 0.05 in the ageing male analysis (Table S4). Key DE proteins seen in the overall ageing analysis including ATP2A2 (SERCA), MYH6, the cathepsin proteins (CTSD, CTSB, CTSZ) and APOE saw similar significant changes in both male and female‐specific analyses. Interestingly, the Mammalian target of rapamycin (mTOR) protein was significantly increased in older females. This significance was lost in the male‐only analysis and in the overall aging analysis. The mTOR signaling pathway plays a role in multiple essential cellular processes including cell proliferation, protein synthesis, autophagy, and survival and has a well‐established role in aging and longevity (Mannick and Lamming 2023).

We next performed Weighted Correlation Network Analysis (WCNA) to identify proteins with expression levels that are highly correlated with one another across two cohorts (Table S5). Highly connected proteins in the network are typically more important to the underlying biology (Han et al. 2004), and therefore, their identification can unveil pivotal proteins and biological processes that regulate and assist healthy ageing of cardiac tissue. Figure 2e illustrates the network analysis, with each node representing a protein and each edge representing correlations between two proteins. NADH:Ubiquinone oxidoreductase subunit AB1 (*NDUFAB1*), myoglobin (*MB*) and superoxide dismutase 2 (*SOD2*) were identified in our network analysis as hubs, which are the most interconnected nodes in the network. The hubs are important network nodes, and their deletion or inhibition might disrupt the healthy ageing process. *NDUFAB1* is a subunit of NADH dehydrogenase or mitochondrial complex I of the mitochondrial electron transport chain and has a role in oxidative phosphorylation and NAD^+^ Synthesis (Sharma et al. 2009). *MB* is an oxygen‐binding protein in striated muscle essential in oxygen storage and delivery (Ordway and Garry 2004). *SOD2* is a mitochondrial antioxidant enzyme pivotal in clearing ROS and protecting against oxidative stress (Palma et al. 2020). After clustering the nodes, the largest cluster of the network contains the “hubs” proteins. The overrepresentation analysis was performed on the most populated and tightly interconnected community of proteins (Figure 2f), and it unveiled that proteins belonging to cellular proteostasis and oxidative phosphorylation play a crucial role in maintaining the healthy aging process. Most proteins in both pathways exhibit a positive correlation.

Additionally, network analyses were performed on the younger and older cohorts separately to identify functional modules specific to each age group (Figure S2, Table S11). As seen in the overall WCNA, cellular proteostasis was a common functional module in the younger and older cohorts. Other functional modules in the younger cohort are related to the oxidative phosphorylation pathways, while the network estimated on older donors showed modules related to DNA damage, nucleotide repair, progeria, as well as mitochondrial complex IV assembly.

### 3.3 Transcriptomics of Younger Versus Older Hearts

RNA sequencing was performed to cross‐examine the transcriptional signatures of cardiac ageing with the results summarized in Figure S3a–d. The RNA data was subsequently used to investigate if the changes to key proteins observed in the proteomics data analysis were the result of a change at the transcript level (Figure S3, Table S2). The log_2_FC of transcript expression between the younger and older cohorts was compared to the corresponding log_2_FC obtained at the protein level (Figure S3e). The fold change of the majority of proteins did not correlate with the fold change seen at the RNA level. To investigate this further, the protein abundance of the DE proteins was compared to the transcript expression of the corresponding RNA (Figure S5). Only one protein showed a significant correlation with the transcript levels (APOE). However, the abundance of key biologically relevant proteins including SERCA2 (ATP2A2), MYH6, and two of the Cathepsin proteins (CTSD and CTSZ) showed no correlation with their corresponding transcript levels. This indicates that these proteins are not directly correlated to the transcriptional regulation of the relevant coding genes, likely due to the non‐linearity of the processes of transcription, translation, and protein synthesis.

Network analysis on the RNA of the younger cohort showed enrichment of multiple modules associated with metabolic processes including oxidative phosphorylation, the TCA cycle, beta oxidation, and glycolysis (Figure S4). Network analysis on the older cohort also identified oxidative phosphorylation as a functional module; however, this was accompanied by functional modules including DNA damage, apoptosis, and nucleotide repair‐associated pathways.

### 3.4 Metabolomics of Younger Versus Older Hearts

Targeted metabolomics was performed to measure polar metabolites in positive and negative ionization modes on the same myocardial tissue using liquid chromatography–tandem mass spectrometry (LC–MS/MS).

**FIGURE 3:**
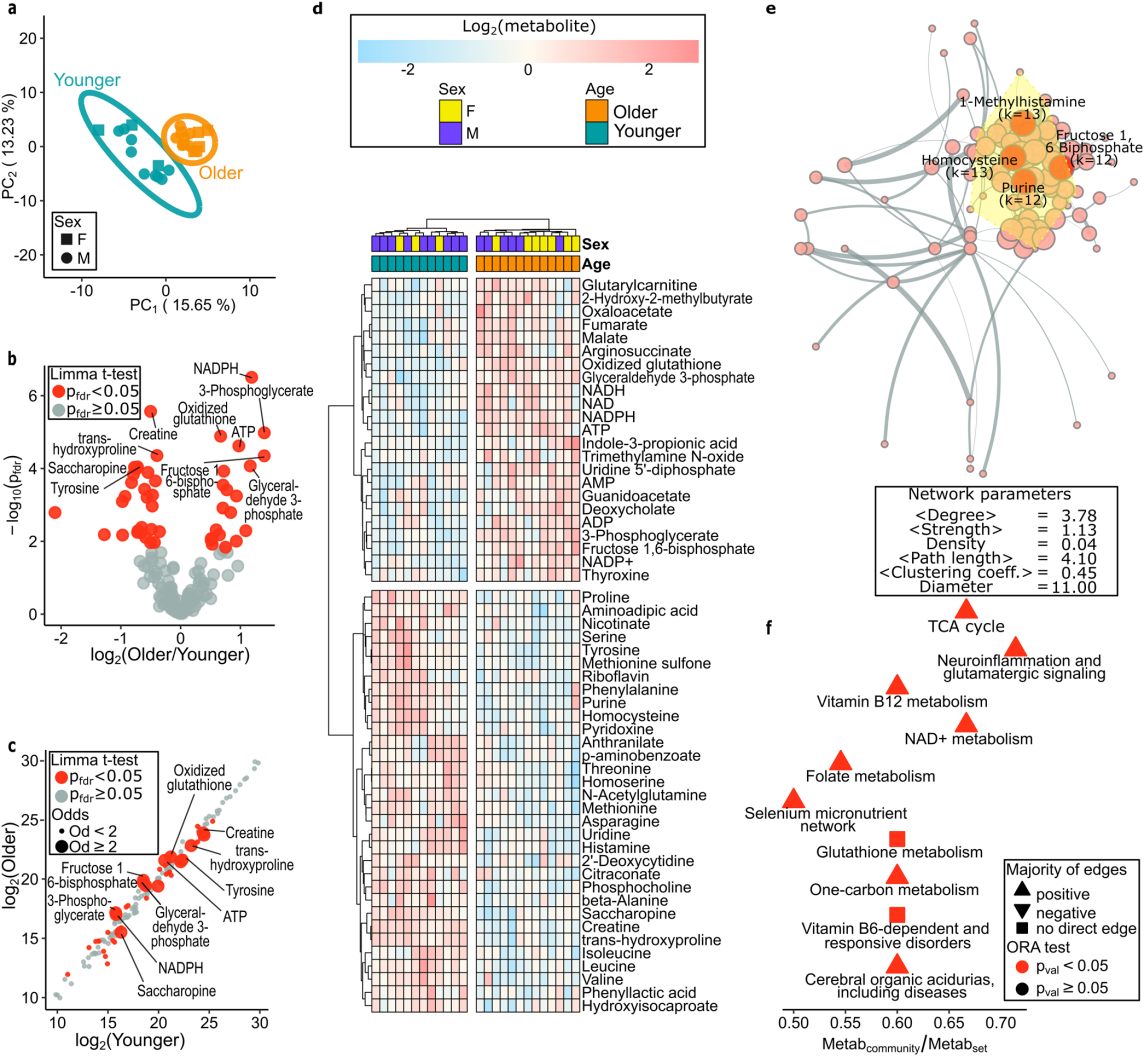
Analysis of metabolite abundances for younger and older hearts. (a) PCA of younger and older hearts. (b) Volcano plot of metabolite changes between the younger and older hearts. (c) Scatter plot of log_2_ metabolite in the younger and older cohorts. (d) Heatmap with hierarchical clustering of samples and metabolites with *P*_fdr_ < 0.01. (e). The nodes have radius proportional to their degree, and edges are proportional to the abs(correlation) between pairs. The nodes with the highest degrees are labelled and the most populated community is highlighted in yellow. (f) ORA of pathways in the largest metabolite community. An up/down‐pointing triangle indicates most correlations are positive/negative, and a square is used when metabolites within a pathway are not directly correlated.

The principal component scores of donors within each cohort exhibit evidence of agreement and good clustering, and no evidence of clustering due to sex was observed. The comparison of metabolite abundances between the younger and older cohort led to the identification of 55 metabolites with *P*_fdr_ < 0.1. Among the 55 metabolites with evidence of dysregulation, 47 were identified as differentially abundant or expressed (*P*_fdr_ < 0.05) in the older cohort (Figure 3b–d, Table S2). The heat map (Figure 3d) suggests a neat separation of younger and older donors in agreement with the PCA.

Several amino acids were decreased in the aged cohort. This included 10 proteinogenic amino acids (tyrosine, leucine, valine, phenylalanine, methionine, isoleucine, proline, threonine, asparagine and serine), with tyrosine being the most significantly decreased amino acid in the older cohort (FC = 0.61, *P*_fdr_ = 1.37 × 10^−3^). There was also a major downregulation in non‐proteinogenic amino acids, such as trans‐hydroxyproline, a major component of collagen, and homocysteine, a recognized biomarker for cardiovascular disease (Veeranna et al. 2011). In addition, there was a reduction in saccharopine, a key intermediate of lysine degradation, and methionine sulfone, an oxidized derivative of methionine.

The older hearts showed changes in metabolites involved in the glutathione antioxidant defense system, which is key in maintaining cellular redox homeostasis. The reduced form of nicotinamide adenine dinucleotide phosphate (NADPH), which provides reducing power to the glutathione system, was the most significantly increased metabolite in the older cohort (FC = 2.28, *P*_fdr_ = 4.71 × 10^−5^). The oxidized form of nicotinamide adenine dinucleotide phosphate (NADP^+^) was also increased in the older hearts (FC = 1.64, *P*_fdr_ = 2.52 × 10^−3^). Considering the NADP^+^ to NADP ratio, the older hearts display a significantly lower ratio than the younger ones (Figure S6c). Oxidized glutathione (GSSG) was increased in old hearts (FC = 1.59, *P*_fdr_ = 4.86 × 10^−4^), suggesting increased oxidative stress. Additionally, the ratio of oxidized to reduced glutathione was increased in older hearts (Figure S6l).

Several metabolites relevant to energy metabolism were altered in the older cohort. Creatine, a key molecule in energy transport and energy reserve reactions within the human myocardium (Balestrino 2021), was the most significantly decreased metabolite in older hearts (FC = 0.71, *P*_fdr_ = 2.02 × 10^−4^). Additionally, guanidoacetate, a precursor of creatine, increased significantly in the older hearts, suggesting a potential defect in creatine synthesis. Adenosine triphosphate (ATP) and adenosine diphosphate (ADP) were significantly increased in older hearts; adenosine monophosphate (AMP), although statistically not significant, showed a qualitative increase with ageing (FC = 0.78, P_*fdr*_ = 0.06). When ADP/ATP and AMP/ATP were considered, both ratios did not exhibit any strong changes associated with ageing (Figure S6i,k, respectively). Additionally, nicotinamide adenine dinucleotide (NAD^+^) and its reduced form (NADH) both significantly increased in the older cohort (FC = 1.43, *P*_fdr_ = 3.1 × 10^−2^, FC = 1.65, *P*_fdr_ = 1.48 × 10^−3^, respectively). Furthermore, NAD^+^/NADH ratio was reduced, indicating a shift to a reduced state in the aged hearts (Figure S6f). Fructose 1,6‐bisphosphate, glyceraldehyde 3‐phosphate, and phosphoglycerate, intermediates of glycolysis, and fumarate and malate, intermediates of the TCA cycle, were all significantly increased in older hearts. When considering the Phosphocreatine/ATP ratio, we observed a decrease in the aged hearts (see Figure 5).

Perhaps reflective of an ageing‐related change to the gut microbiome, there was an increase in metabolites created by gut microbiota in the older cohort. Indole‐3‐propionic acid, a microbial tryptophan derivative that is emerging as an important protective factor in a range of CVD, including atherosclerosis (Xue et al. 2022) and heart failure with preserved ejection fraction (HFpEF), was significantly increased in older hearts. The atherogenic metabolite trimethylamine N‐oxide and deoxycholate, a secondary bile acid produced by intestinal bacteria from the primary bile acid, cholic acid (Yang and Qian 2022), were also significantly increased in older hearts.

The overall WCNA performed on all metabolites abundances allows us to investigate further the metabolites that act as hubs and the possible functions that play a crucial role during ageing (Figure 3e, Table S6). Homocysteine, 1‐Methylhistamine, Fructose 1,6 Biphosphate, and Purine are the nodes acting as hubs and likely key players in the ageing process. Clustering coupled with overrepresentation analysis further reveals that the TCA cycle and NAD^+^ metabolism are among the pathways that represent the largest community of the network, where the majority of the analytes in each pathway positively correlated in ageing (Figure 3f). When the younger and older cohorts were analysed separately (Figure S7, Table S11), NAD biosynthesis and Alanine and aspartate metabolism were enriched functional modules in the younger cohort. The network analysis for the older cohort did not show any evidence of enrichment for any functional modules.

### 3.5 Lipidomics of Younger Versus Older Hearts

**FIGURE 4:**
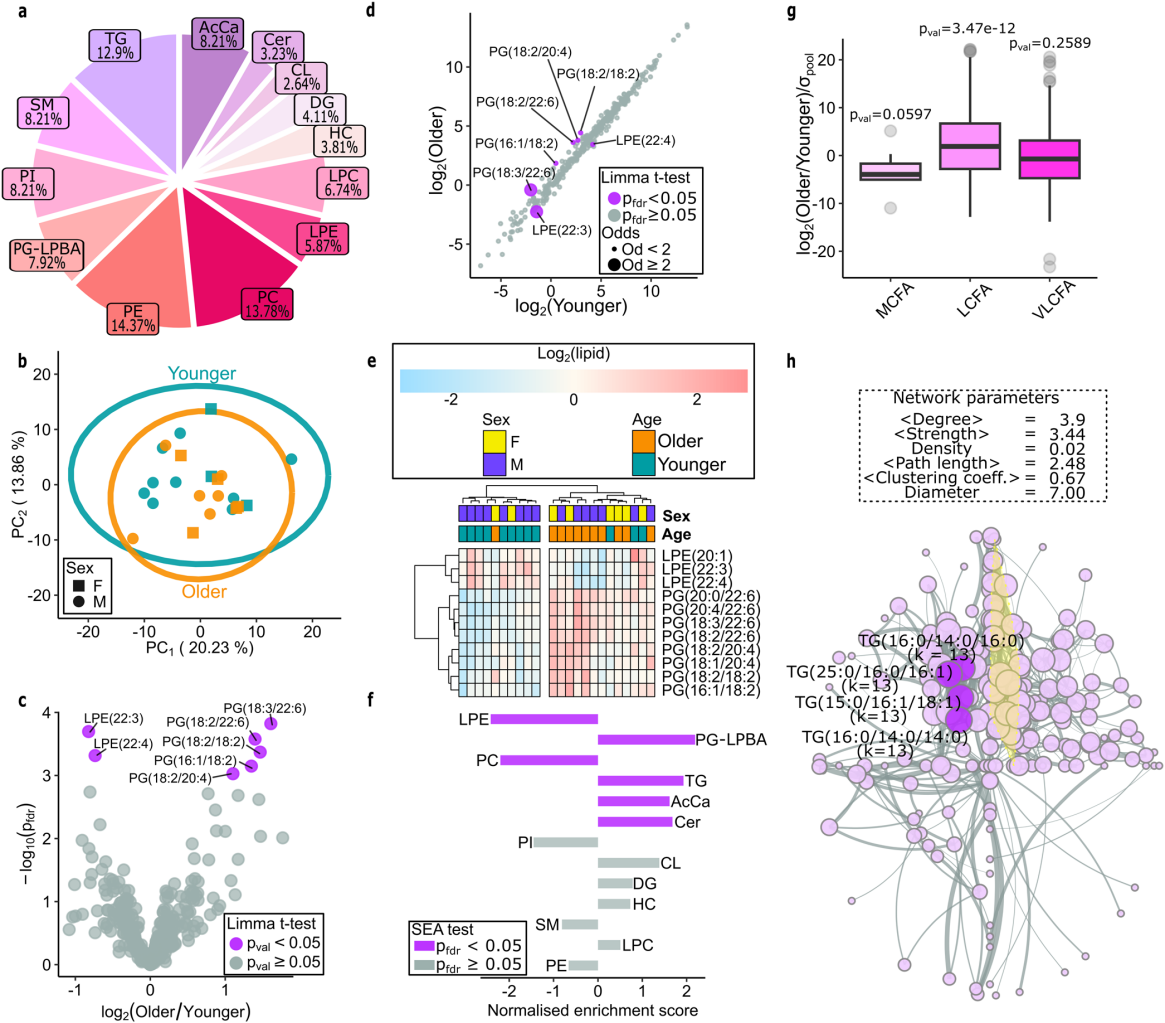
Analysis of lipid abundances between younger and older hearts. (a) Lipid classes detected in donor hearts. (b) PCA of younger and older hearts. (c) Volcano plot of lipid changes between the younger and older cohorts. (d) Scatter plot of log_2_ lipid in younger and older hearts. (e) Heatmap with hierarchical clustering of samples and lipids with *P*_fdr_ < 0.1. (f) Analysis of lipid classes. (g) Analysis of lipid's fatty acid tails. (h) WCNA. The nodes have radius proportional to their degree, and edges are proportional to the abs(correlation) between pairs. The nodes with the highest degrees are labelled and the most populated community is highlighted in yellow.

Untargeted lipidomic analysis of the cardiac tissue resulted in the quantification of 342 lipids, covering 13 different lipid classes, including glycerophospholipids (phosphatidylcholine, PC; phosphatidylethanolamine, PE; phosphatidylglycerol/lysobisphosphatidic acid, PG/LBPA; phosphatidylinositol, PI; cardiolipin, CL; lysophosphatidylcholine, LPC; and lysophosphatidylethanolamine, LPE) glycerolipids (triglyceride, TG; and diacylglycerol, DG), fatty acyls (acylcarnitine, AcCA) and sphingolipids (ceramide, Cer; hexosylceramide, HC; and sphingomyelin, SM). PG and LBPA have identical molecular weights and therefore were not differentiated by mass spectrometry (Fahy et al. 2009). When considering lipids grouped by class, the PE's comprised the largest portion of lipids in the data (Figure 4a).

The PCA demonstrated that clusters of the younger and older cohorts were not as distinct as for the proteomics and metabolomics, yet agreement across observations within each cohort can be observed (Figure 4b). In contrast, the sample‐wise hierarchical clustering associated with the heat map showed better cluster properties (Figure 4e). Differential expression analysis between the younger and older cohorts identified a total of 7 lipids that had age‐related changes (Figure 4c–e). All five upregulated lipids belonged to the PG/LBPA class (18:3/22:6, 18:2/22:6, 18:2/18:2, 16:1/18:2, and 18:2/20:4), while the two downregulated lipids were part of the LPE class (22:3 and 22:4).

The enrichment analysis identified 6 lipid classes that exhibit overall changes across the younger and older cohorts (Figure 4f, Table S7). Consistent with the individual lipid analysis, the PG‐LBPA's, with LPBA being a late endosome and lysosome‐specific phospholipid (Hullin‐Matsuda et al. 2009), were increased in the older cohort. The AcCa's, which play an essential role in shuttling fatty acids into the mitochondria for β‐oxidation (Aitken‐Buck et al. 2020), and TG's, which have a clear association with atherogenic CVD (Talayero and Sacks 2011) and are stored in the heart under metabolic stress, including obesity and T2D (Goldberg 2018) were also statistically enriched in the older cohort.

Cer's, which are predictive biomarkers of cardiometabolic complications (Choi et al. 2021), were also increased in the older hearts. Ceramides play an important role in many key cellular pathways including inflammation and oxidative stress. To investigate the relationship between ceramide levels and oxidative stress in the human heart samples, oxidative stress makers in our datasets (proteomics, transcriptomics and methbolomics) were retrieved and compared to levels of various ceramides. Multiple ceramides significantly correlated with protein and RNA of oxidative stress markers (Figure S8, Table S13) including glutathione peroxidase 1 (GPX1), glutathione peroxidase 3 (GPX3), transcription factor Sp1 (SP1), C‐fos (FOS), Nuclear Factor Kappa B Subunit 1 (NFKB1), and Monoamine oxidase A (MAOA). Significant correlations between metabolites oxidised glutathione and uracil and ceramides were also observed. This confirms the clear connection between ceramides and oxidative stress, which both play a role in the ageing process.

We then considered differences between fatty acid chain lengths and the number of double bonds between younger and older hearts (Figure S9e–g). Overall, older hearts had an increased abundance of LCFAs when compared to young hearts; an opposite trend is shown for the medium chain fatty acids (Figure 4g). When considering specific lipid classes, the increased Cer and TG lipids in the older hearts were predominantly LCFAs (Figure S9d,e, respectively).

Finally, overall WCNA revealed that lipids acting as hubs in cardiac ageing belong to the TG class (Figure 4h, Table S9). The ORA performed on the largest community of the network identified that TG lipids are representative of the ageing process, and most of the species in the community exhibited a positive correlation (Figure S4b). When the network analyses were performed separately on each cohort (Figure S10, Table S11), there was no enrichment of any particular modules in the younger cohort. Interestingly, AcCAs were the only representative module enriched in the older cohort, which is complementary to the increase of AcCAs seen in the older hearts.

### 3.6 Analysis of Metabolic and Mechanical Processes

**FIGURE 5:**
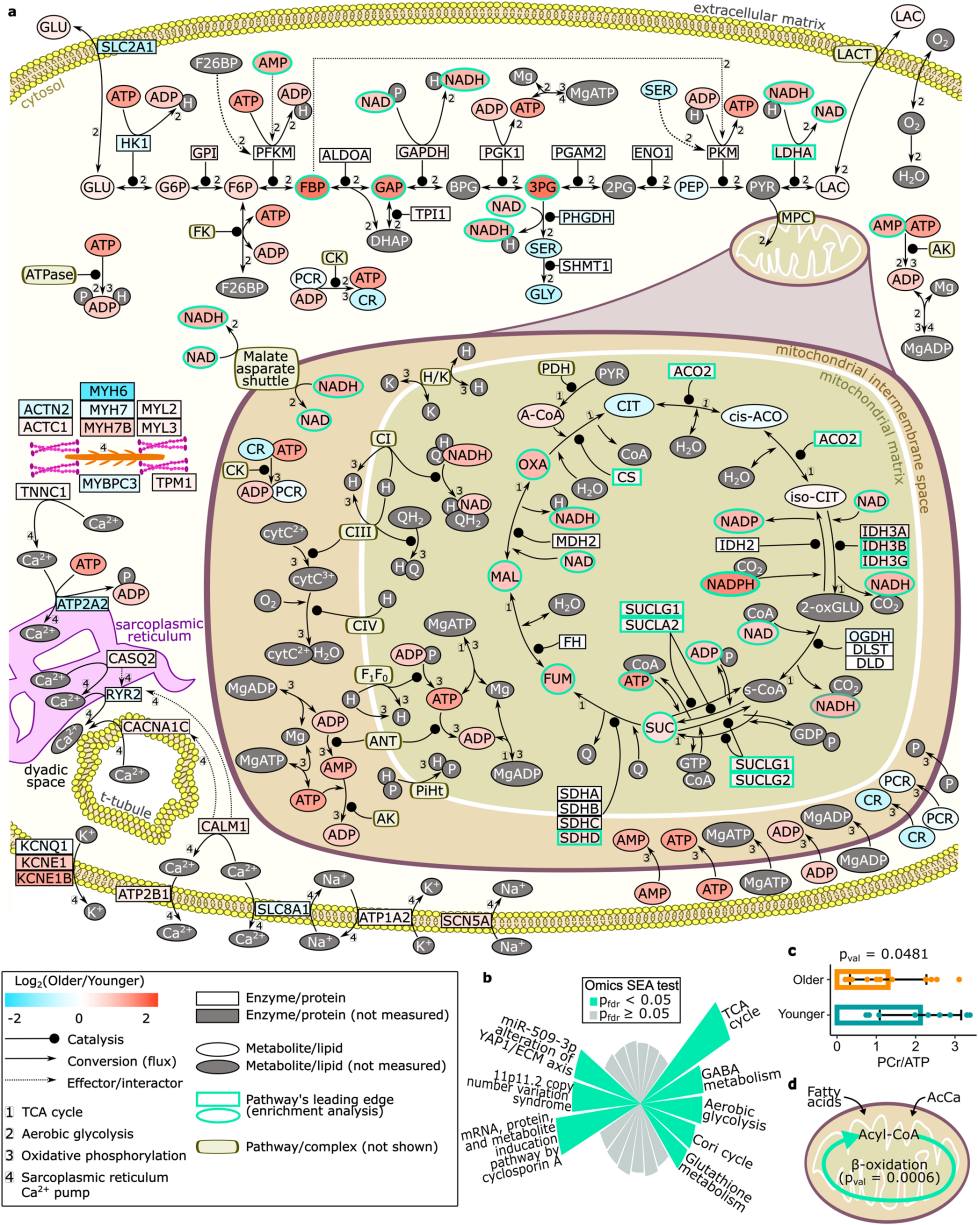
Metabolic and mechanical processes in the ageing heart. (a) Core metabolic and mechanical network interlinking the TCA cycle, aerobic glycolysis, and oxidative phosphorylation with the contractile machinery and calcium signalling. The analytes are coloured based on the log_2_ (older/younger). The analytes with aquamarine borders are the leading edges of the omics Set Enrichment Analyses (omics SEA). (b) Omics SEA. The enriched pathways with *P*_fdr_ < 0.05 are highlighted, and the bars are proportional to the *P*_val_ of each pathway. (c) PCr/ATP ratio. (d) Dysregulation of β‐oxidation detected via omics SEA.

A schematic capturing the comprehensive changes, along with subcellular localisation, of enzyme, metabolic, and lipid is illustrated in Figure 5a, which highlights protein and metabolite changes in hearts at a cardiac bioenergetics level, including the glycolysis pathway, TCA cycle, and oxidative phosphorylation. The changes in the contractile apparatus and sarcoplasmic reticulum are also highlighted. The omics Set Enrichment Analysis was performed on the proteomics, transcriptomics, metabolomics, and lipidomics data (Figure 5b, Table S10). The analyses identified eight dysregulated pathways in older hearts, five belonging to the cell's metabolism. Among the metabolic pathways, aerobic glycolysis (Set size = 20, *P*_fdr_ = 1.99 × 10^−2^) and TCA cycle (Set size = 32, *P*_fdr_ = 9.71 × 10^−4^), pivotal components of the central carbon metabolism, presented substantial evidence of dysregulation. The Cori cycle (Set size = 31, *P*_fdr_ = 3.39 × 10^−2^), a chemical pathway responsible for lactate metabolism, also exhibited significant age‐related changes. Evidence of aberrant regulation was also found for the glutathione metabolism (Set size = 24, *P*_fdr_ = 3.68 × 10^−2^), with possible consequences for the cellular oxidative stress. The GABA metabolism, or GABAergic system, was also dysregulated (Set size = 20, *P*_fdr_ = 1.93 × 10^−2^), possibly affecting the Ca^2+^ cardiac dynamics in the cardiomyocytes. The age‐related metabolic changes were also confirmed by the Phosphocreatine/ATP ratio (Figure 5c). Based on the literature, a manually curated beta‐oxidation was reconstructed to account for the AcCa lipids detected in our study. The oxidation of the fatty acids displayed evidence of alteration (Set size = 54, *p*_val_ = 5.84 × 10^−4^) with ageing (Figure 5d, Figure S11).

In summary, proteins essential for efficient contraction are mostly decreased in the older heart, including *SERCA2* (or *ATP2A2*) and *MYH6*. The central cardiac energetic pathways (glycolysis, TCA cycle, beta‐oxidation) were impaired in older hearts, also confirmed by the decrease in the phosphocreatine/ATP ratio.

### 3.7 In Silico Investigation of Calcium Signalling, Contraction, and Oxidative Phosphorylation in Cardiomyocytes

**FIGURE 6:**
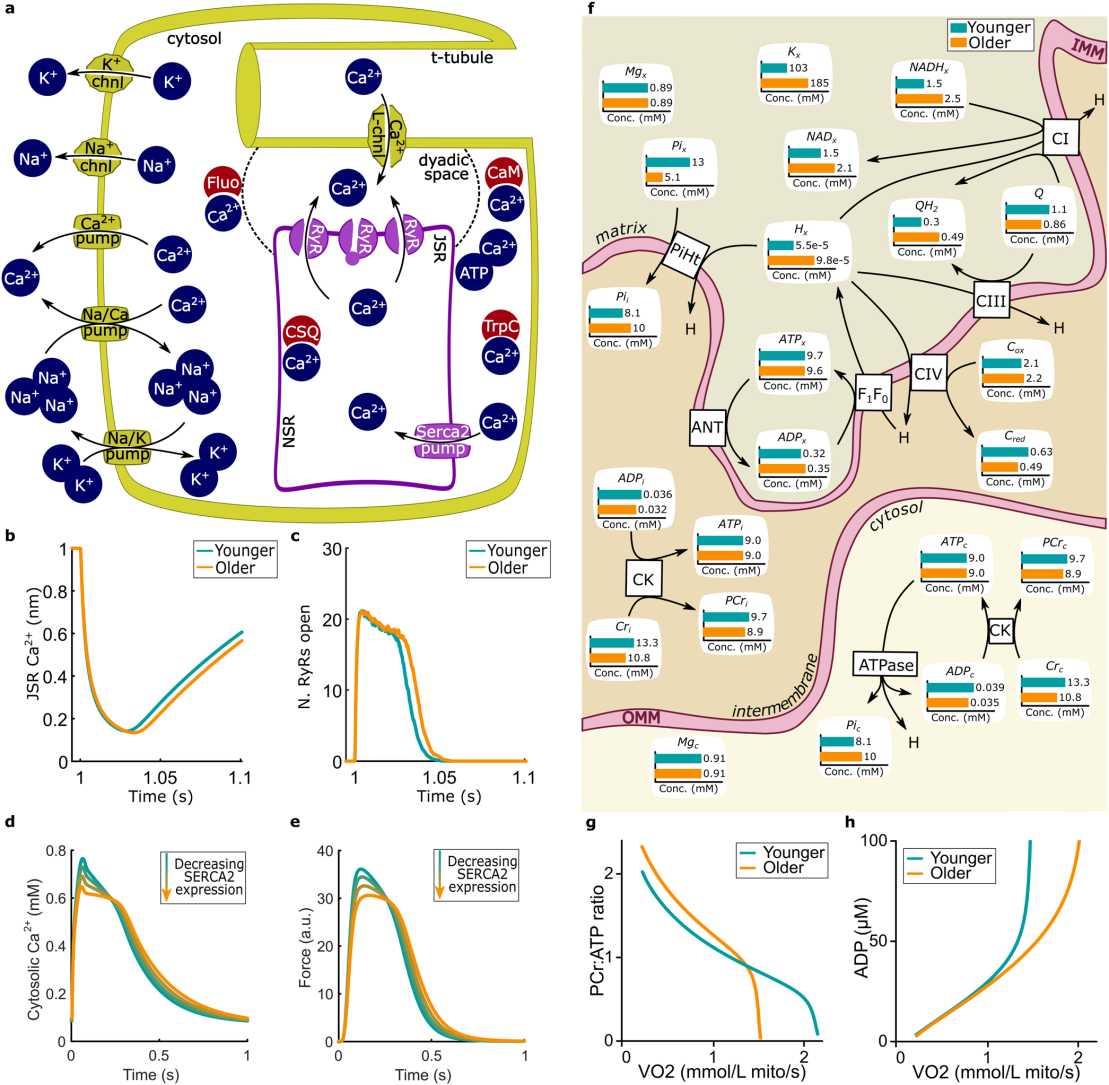
Biophysical modelling of calcium signalling and oxidative phosphorylation. (a) A schematic of cardiac electrophysiology and calcium handling in a cell. (b, c) A stochastic computational model of the dyad was used to perform 200 simulations of calcium‐induced sparks, and the average results are shown for (b) JSR Ca^2+^ and (c) RyR channels. (d, e) A deterministic model of electrophysiology was coupled to models of calcium release and cellular contraction, and timeseries were plotted for (d) cytosolic Ca^2+^ and (e) contractile force. The activity of *SERCA2* was varied from a high (green) to low (orange) value. The simulations show lower peaks and longer transients for both cytosolic Ca^2+^ and contractile force. (f) A schematic of the biophysical model of oxidative phosphorylation, along with predictions of metabolite concentrations under a high workload. Predictions for younger cardiomyocytes are in green, and predictions for older cardiomyocytes are in orange. The models for the younger and older cohorts were simulated over different workloads, and (g) the PCr/ATP ratio and (h) ADP concentrations were plotted. The model predicts that older cardiomyocytes maintained a higher PCr/ATP ratio and lower ADP concentration at higher workloads.

Biophysically constrained computational models of cardiomyocyte calcium handling (Figure 6a) and oxidative phosphorylation (Figure 6f) were employed to gain additional insights into the possible effects of decreased expression of proteins related to contraction and increased abundance of cardiac energetics‐related metabolites in older hearts. A suite of biophysics‐constrained computational models of cardiac electrophysiology, calcium handling, and energetics has been created over the last 20 years (Beard 2005; Rajagopal et al. 2022; Vendelin et al. 2000). These models encode physiological processes of interest with equations that are constrained by thermodynamics and Fick's law of diffusion. These models treat concentrations of each species as dynamic variables in a differential equation, with fluxes through reactions and transporters determining their rates of change.

We first used a computational model of the cardiac dyad to computationally infer the potential impact of reduced *SERCA2* expression with age on elementary calcium release events involved in excitation–contraction coupling, the calcium spark (Cheng et al. 1993). The model (Figure 6a) simulates stochastic gating of Ryanodine Receptors (RyRs) and subsequent binding and unbinding of calcium to buffers, its diffusion through the dyad and cytosolic space, and its re‐uptake into the SR by *SERCA2*. This model was adapted from a previously published computational study (Chung et al. 2023) and parameterized to sheep cardiomyocytes to more closely simulate calcium sparks in larger mammals. Simulations of the baseline model were used to represent sparks in young cardiomyocytes, while simulations with the previously calculated log_2_FC, −0.53233, applied to the *SERCA2* activity parameter represented sparks in older cardiomyocytes. Our simulations predict an increase in calcium spark duration in older hearts. This is evidenced by an increase in duration on average for calcium in the junctional SR to nadir (Figure 6b) and for RyRs to close completely for spark termination (Figure 6c).

The effect of a reduced *SERCA2* expression with age on the global calcium transient and additional excitation‐contraction coupling properties was examined next. To this end, a lumped‐parameter coupled ordinary differential equation (ODE) model of human cardiomyocyte electrophysiology, calcium handling (Iyer et al. 2004) and cardiac contractility (model 5 in ref. (Rice et al. 1999)) was employed. The model generated physiological calcium transients when paced at 1 Hz (Figure 6d). The baseline parameter for *SERCA2* activity in the model was scaled down to 2^−0.53233^ at regular intervals to simulate the incremental effect of reduced *SERCA2* expression observed in older hearts. When the abundance of *SERCA2* was reduced (from orange to green lines), the calcium transient exhibited a lower peak and longer duration due to slower calcium reuptake. This leads to a downstream effect on cardiac contractility as indicated by the decreased force amplitude and longer duration in the contracted state (Figure 6e). The prolonged calcium transient is also consistent with the longer spark duration predicted by the dyad model. These computational results also corroborate our omics‐based pathway analysis conclusion that the downregulation of *SERCA2* in older hearts may contribute to poorer heart contractility.

We then investigated the potential impact of changes in metabolite abundance on mitochondrial oxidative phosphorylation and cardiac bioenergetics using a bond‐graph approach (Gawthrop and Crampin 2014; Gawthrop and Pan 2022; Pan et al. 2021) (see model details in Supporting Information, Item S1). With sparse human‐specific data on mitochondrial oxidative phosphorylation enzymes and reaction rates, we based the model on previously published experimental and computational studies on bioenergetics in rat hearts (Beard 2005). Creatine kinase enzymes were included in the model to infer the potential effects of alterations to creatine abundance we observed in old hearts. The young cohort was taken to be the baseline model, with fold changes applied to simulate older hearts. Because the metabolomic analysis found that NAD^+^ and NADH were upregulated in older hearts, oxidative phosphorylation in older hearts was simulated by increasing NAD^+^ and NADH concentrations by the magnitudes measured in the metabolomics data (+43% and + 65%, respectively). Similarly, the initial conditions for creatine and phosphocreatine were decreased by 29% and 8%, respectively, in accordance with the fold changes observed in the metabolomics data. Model predictions of energy consumption under a high workload (1.21 mol O_2_/L mito/s) are shown in Figure 6f. The model predicts changes in the electron carrier ubiquinone (Q) and cytochrome c; the reduced form of ubiquinone (QH_2_) and oxidized form of cytochrome c (C_ox_) are favored in older hearts. Lower phosphate and higher proton concentrations were also seen in the matrix of older cardiomyocyte mitochondria. To explore the effects of age at a wider range of workloads, the phosphocreatine (PCr) to ATP ratio and ADP concentration were plotted against workload. At low workloads, the model predicted that younger cardiomyocytes maintained a higher PCr/ATP ratio due to the higher amount of total creatine available (Figure 6g). However, older hearts were able to maintain a higher PCr/ATP ratio at higher workloads due to the enhanced availability of NADH (Figure 6g). ADP concentration remained low at higher workloads in older hearts (Figure 6h). Since low PCr/ATP and higher ADP concentration ratios are associated with metabolic derangements in HF (Neubauer 2007) the changes to NAD^+^/NADH may be seen as protective against HF in older hearts at high workloads.

## 4 Discussion

Ageing is the largest risk factor for most non‐communicable diseases, including CVD. We provide the most comprehensive molecular analysis and resource of human cardiac ageing to date, employing a multi‐omics approach that outlines changes at the gene, protein, metabolite, and lipid levels. Additionally, we mapped our data to single‐cell computational models, which allowed extrapolation of the molecular changes to cardiomyocyte functional outcomes. The previous few studies that have examined human cardiac aged tissue have been limited to the use of pathological cardiac tissue from patients undergoing bypass surgery (Gergs et al. 2019) or non‐diseased post‐mortem human hearts (Choudhury et al. 2022; Gazoti Debessa et al. 2001). By examining pre‐mortem healthy heart samples across the lifespans, we have identified important changes related to the general ageing process as well as cardiomyocyte‐specific changes.

The downregulation of the SERCA2 in older hearts suggests an underlying diastolic dysfunction in normal ageing (Nayor et al. 2018). *SERCA2* is an intracellular pump responsible for Ca^2+^ reuptake into the SR, reducing Ca^2+^ concentration in the endoplasm after contraction (Eisner et al. 2020; Park and Oh 2013). The rate of Ca^2+^ uptake by the SR determines myocardial relaxation rate, which affects cardiac function (Zhihao et al. 2020). *SERCA2* knockout mice and heterozygous *SERCA2* mutant mice exhibit considerable reductions in diastolic function (Andersson et al. 2009; Periasamy et al. 1999), supporting its importance in normal cardiac relaxation. The decrease in *SERCA2* we have observed in human cardiac aging is reflected in rodent models of ageing (Bodyak et al. 2002; Dai et al. 2009; Jiao et al. 2012; Taffet and Tate 1993). Additionally, *SERCA2* expression and activity are decreased in rodent and human HF studies and are a potential therapeutic target to augment cardiac function in HF patients (ClinicalTrials.gov: NCT04703842) (Eisner et al. 2013; Greenberg et al. 2016; Hasenfuss et al. 1994; Romeo et al. 2023). As *SERCA2* is essential to diastolic function, its decrease suggests impaired Ca^2+^ reuptake into the SR after contraction, delaying active relaxation of cardiomyocytes and resulting in impaired diastolic function. Our biophysical model supports this hypothesis, as decreased *SERCA2* in cardiomyocyte calcium signaling resulted in delayed Ca^2+^ reuptake into the sarcoplasmic reticulum and reduced contractility. A reduction in *SERCA2* may be an early molecular signature of cardiac aging and may be important in age‐associated diastolic dysfunction.

Furthermore, *MYH6* was decreased in older hearts, which may be another important factor in reduced cardiac function as we age. Human hearts predominately contain β‐myosin heavy chain (*βMyHC* or MYH7), with *MYH6* representing approximately 7% of the total amount of myosin heavy chain within cardiomyocytes (Miyata et al. 2000). Despite its relatively low abundance, myosin containing *MYH6* has a higher ATPase activity, resulting in greater shortening velocity and power generation (Rundell et al. 2005). A study comparing cardiac muscle from rats aged 2, 6, and 24 months showed that *MYH6* declined with age (Long et al. 1999). Additionally, levels of *MYH6* decrease to < 1% of the total myosin heavy chain content in failing human hearts (Miyata et al. 2000). A mathematical model of myocardial isometric twitch kinetics predicts that even the low level of *MYH6* seen in healthy myocardium significantly enhances twitch kinetics and systolic function compared to a state where the isoform is essentially absent, as it is in HF (Locher et al. 2011). Therefore, a reduction in *MYH6* may lead to a less efficient contraction as we age, negatively impacting cardiac function. And like *SERCA2*, it may be an early molecular signature of cardiac ageing.

While the protein levels of both SERCA2 and MYH6 significantly decreased in older hearts, the same pattern is not observed at the transcript level. This suggests that these proteins are not decreasing in aged hearts due to a change in transcriptional regulation. Interestingly, the majority of proteins analysed did not show a correlation with their corresponding RNA. While it is well established that mRNA levels do not always correlate with protein levels due to post‐transcriptional and post‐translational modulators, it has been shown that post‐transcriptional dysregulation and discordance between mRNA and protein levels increases with age (Anisimova et al. 2020; Takasugi et al. 2024; Wei et al. 2015). Therefore, further work to understand how post‐transcriptional regulators, such as miRNAs, are altered as we age is essential to help further help characterise the molecular changes associated with human cardiac ageing.

Sex specific analysis of the proteomics data saw many of the key DE proteins, including SERCA and MYH6 changing in both males and females. However, interestingly, mTOR was only significantly increased in older females, suggesting a sex specific involvement of mTOR in cardiac ageing. The mTOR signaling pathway plays a role in multiple essential cellular processes including cell proliferation, protein synthesis, autophagy, and survival and has a well‐established role in ageing and longevity (Mannick and Lamming 2023). Additionally, mTOR has been implicated specifically in cardiac ageing (Dai et al. 2023). Therefore, it is an interesting, biologically relevant result. The specific change in the females is a novel finding. It is known that females have a higher risk of developing HFpEF (Scantlebury and Borlaug 2011; Sotomi et al. 2021) and therefore further investigations into how mTOR signaling changes in the hearts of females as they age and the relationship to diastolic dysfunction may be necessary, but beyond the scope of the current study.

The metabolomic findings also suggest early deleterious changes in older hearts. For example, creatine, which has an essential role in buffering and transporting chemical energy in cardiomyocytes, decreased in older hearts. A decrease in creatine is also noted in HF (Ingwall 2009; Ten Hove et al. 2005). The decrease in creatine in older hearts may result from impaired synthesis, as a precursor of creatine, guanidinoacetate, was elevated in older hearts. Additionally, there was a global decrease of proteogenic amino acids in older hearts. These include the three branched‐chain amino acids (BCAAs)—isoleucine, leucine, and valine—important energetic substrates of the heart under times of stress. Suppression of BCAA catabolism is implicated in maladaptive remodelling in HF (Li et al. 2017; Neinast et al. 2019; Wang et al. 2016) resulting in BCAA accumulation. Our results indicate that BCAA accumulation is not characteristic of healthy myocardial aging. The decrease of BCAA with aging in the cardiovascular system of healthy individuals has been reported in a study conducted on plasma samples of an ethnically Chinese subgroup of the Singaporean population (Chen et al. 2020). Similar results on human plasma were reported in previous studies (Chaleckis et al. 2016; Le Couteur et al. 2020). Interestingly, while BCAAs are higher in individuals with Heart Failure, BCAA supplementation is often associated with increased lifespan of mammals, although further studies are needed (D'Antona et al. 2010).

Changes that are reflective of general aging were also observed in our study. Several of the cathepsin proteins, major components of the lysosomal proteolytic system, were increased in older hearts. Complementing these results, a lysosomal lipid class, LBPA, was also increased. Lysosomes play an essential role in cell and tissue homeostasis through their key function of cellular degradation. Protein homeostasis is disrupted during the aging process, which leads to accumulation of damaged proteins (Vilchez et al. 2014). Damage that occurs during aging leads to loss of cells with myocardial aging and disease pathologies (Goldspink et al. 2003). This damage therefore increases the demand on systems responsible for removing damaged cellular structure, that is, lysosomes (Terman et al. 2008), leading to an increase in the number and size of lysosomes (Fernandez‐Sanz et al. 2014), which may explain the increase we see in the lysosomal proteins in the older hearts. Specifically, cathepsin D (*CTSD*) was the most significantly upregulated protein in older hearts. A recent study has demonstrated that elevated circulating levels of cathepsin D in HF patients are associated with a more severe disease phenotype (Hoes et al. 2020). The same study also showed that human stem cell‐derived cardiomyocytes released cathepsin D in response to mechanical stretch, with silencing of cathepsin D inducing necrosis, increased stress‐induced metabolism, and increased release of troponin T when under stress, indicating that cathepsin D may be necessary for cardiomyocyte survival when under stress. With relevance to cardiac aging, cathepsin B has been shown to directly affect cardiac hypertrophy and fibrosis (O'Toole et al. 2020; Wu et al. 2015), both of which increase in human and animal studies of cardiac ageing (Lakatta 2003; Lu et al. 2017).

Oxidative stress is commonly observed with ageing, and the production of reactive oxygen species (ROS) has the strongest correlation with overall longevity (Finkel and Holbrook 2000). Glutathione acts as an important antioxidant by facilitating the reduction of hydrogen peroxide in the presence of glutathione peroxidase, resulting in the production of oxidized glutathione. Oxidized glutathione is then reduced back to glutathione by glutathione reductase. In the current study, the older hearts had elevated oxidized glutathione, which is most commonly an indication of oxidative stress causing glutathione reductase to become rate limiting, preventing reduction of oxidized glutathione, therefore leading to its accumulation (Lu 2009). Elevated NADPH in the older cohort is suggestive of the heart trying to combat this increase in oxidative stress. Both NADP^+^ and NADPH are important mediators of intracellular redox reducing capacity. NADPH enables the glutathione system to eliminate ROS and plays a major role in metabolism and antioxidant defence (Xiao and Loscalzo 2020). An increase in NADPH may indicate an elevated capacity and/or requirement to handle redox stress in older hearts with a shift towards increased antioxidant defense.

A decrease in NAD^+^ levels has been implicated in the ageing process and in the development of HF (Abdellatif et al. 2021). Conversely, we observed an increase in both the NAD^+^ and NADH pools in older hearts. To date, NAD^+^ decline in human ageing has been shown only in brain (Zhu et al. 2015), skin (Massudi et al. 2012), plasma (Clement et al. 2019) and skeletal muscle (Janssens et al. 2022). This discrepancy may be due to differences in cohort ages, with the old cohort used in the skeletal muscle study aged 65–80 years old, which is much older than the cohort of the current study, with the age range being 51–65 years old. Additionally, the same study demonstrates that a portion of the old cohort who are very physically active had NAD^+^ levels comparable to the young cohort, suggesting a close relationship of physical activity and preservation of NAD^+^ levels, and not a clear‐cut relationship between NAD^+^ levels and age alone. Notably, NADH was elevated to a greater degree than NAD^+^, with a significant decrease in the NAD^+^/NADH ratio seen in the old hearts. This is consistent with an elevated need for cardiac cells to mitigate redox stress and consistently observed in healthy ageing in rodent models (Braidy et al. 2011) and human models (Clement et al. 2019; Zhang et al. 2020). However, it is important to note that the biosynthesis and degradation of NAD^+^ are highly compartmentalised and regulated by specific enzymes (Cambronne and Kraus 2020) and to fully understand the dynamics of NAD^+^ levels, measuring NAD^+^ levels in different cellular compartments, such as mitochondria, would be beneficial. A change in the NAD^+^/NADH ratio may also be related to key changes associated with animal models of cardiac ageing. Changes in NAD^+^ levels and the NAD^+^/NADH ratio play a role in regulating the catalytic activity of sirtuins, a class III histone deacetylases. Sirtuin activity regulates cardiac ageing and influences cardiac hypertrophy (Alcendor et al. 2007). Therefore, further investigation into the activity of sirtuins in response to a decrease in the NAD+/NADH ratio seen in aged human hearts is warranted in future studies.

Changes in lipid profiles have also been shown to occur as we age and are linked to the development of CVDs (Hsu et al. 2021; Kaneko et al. 2021; Kawanishi et al. 2018). Specifically, evidence suggests that Cers are cardiotoxic (Choi et al. 2021; Park et al. 2008) while TG's have a clear association with atherogenic CVD (Talayero and Sacks 2011). Plasma levels of Cers and TGs have been shown to increase in healthy ageing (Gui et al. 2020; Spitler and Davies 2020; Vozella et al. 2019). Additionally, TG levels have been seen to increase in heart tissue of aged mice (Eum et al. 2020). Our data suggest that this increase is also reflected in human cardiac tissue as we saw both classes of lipids increase in older hearts. Interestingly, Cers and TGs with long chain fatty acids were more abundant in the older hearts, a result also observed when considering all lipid classes combined. This change has also been reflected in the blood, with the plasma lipid profile of healthy individuals older than 50 years skewed towards long chain fatty acids (Li et al. 2017; Neinast et al. 2019; Wang et al. 2016). Additionally, fatty acid chain length has been demonstrated to play a role in regulating healthy ageing as it inversely correlates with longevity (Murashige et al. 2020).

In relation to energy metabolism, oxidation of fatty acids is the main source of energy production in the heart. Long‐chain fatty acids enter the mitochondria for oxidation with the help of the carnitine transport system, while medium‐chain fatty acids can freely diffuse directly into the mitochondria (Schönfeld and Wojtczak 2016). Our lipidomics data show an increase in AcCas in older hearts. AcCas have an essential role in beta‐oxidation by acting as a carrier to transport long‐chain fatty acids into the mitochondria. Additionally, we saw an increase in TGs in older hearts. These results suggest that there is a reduced consumption of AcCas and TGs by the beta‐oxidation pathway in older hearts. Based on fold change, carnitine, which facilitates the transport of AcCas into the mitochondria, is decreased in older hearts, which may indicate an issue with the transport of substrates into the mitochondria for beta‐oxidation is responsible for the accumulation we see. This possible reduced substrate availability may result in increased glycolysis to compensate. We also observed increased glycolytic intermediates in older hearts and an increase in glycolysis (Figure 5a). The increase in the use of glucose as a substrate for energy production has been consistently observed in early studies of cardiac ageing in rodents (McMillin et al. 1993). Additionally, it is reflected in a human study where PET scans were used to measure fatty acid and glucose utilization when comparing young and older individuals (Kates et al. 2003). This compensatory increase in glycolysis substrates may indicate a decrease in the ability of the aging heart to extract maximum ATP from fatty acid oxidation, the most costly in terms of oxygen consumption. However, flux studies are necessary in the future to confirm this hypothesis. Interestingly, our network analysis of proteins and RNA demonstrated enrichment of functional modules related to metabolism in the younger cohort, while not for the older cohort, possibly due to metabolic inflexibility that increases with ageing of the myocardium (Zhou et al. 2023). In relation to cardiac energetics, we observed an increase in ATP in older hearts. This result may be indicative of either an increase in ATP generation or a decrease in ATP utilization. Increased intermediates of the TCA cycle and an overall increase of the TCA cycle, in addition to a reduced NAD^+^/NADH ratio (NAD^+^ is a main enzymatic cofactor in energetic reactions) as seen in our study, support increased energy generation. These findings indicate a, perhaps compensatory, elevation of energy production in older hearts to necessitate increased energy requirements to sustain optimal contraction due to dysregulation of calcium handling (decreased *SERCA2*) and contractile function (decreased MYH6). The aberrant energy status of the heart is also confirmed by the decrease in PCr/ATP ratio (Yaniv et al. 2013). By increasing NAD^+^ and NADH to the levels seen in our metabolomics results, a biophysical in silico experiment suggests that under high workloads, older cardiomyocytes maintained a higher PCr/ATP ratio and lower ADP concentrations, further indicating a compensatory change to cardiac energetics in older hearts. Additionally, decreased ATP utilization may also be contributing to the increase in ATP in older hearts. Myosin ATPase's and SERCA are the two largest consumers of ATP in the heart (Tran et al. 2015). Therefore, a reduction in these proteins will likely impact ATP utilization. This is supported by an overall decrease in the ADP/ATP ratio seen in older hearts, although not statistically significant (*P*_val_ = 0.1289), indicating less conversion of ATP to ADP. As the decrease in levels of mechanical proteins did not seem to occur in tandem with a decrease in energy provision, it is possible that decreased mechanical efficiency led to a “backup” or accumulation of energetic substrates. However, in HF, there is a decrease in both the mechanical and energetic apparatus (Balaban 2002), and it may be that preservation of energy generation is what prevents the *ageing heart* from becoming the *failing aged heart*. Future mechanistic studies are needed to fully elucidate the reason for the increased ATP seen in older hearts.

A limitation of the study is the small sample size, attributed to the difficulty in obtaining healthy human hearts, particularly hearts spanning the age range needed to investigate cardiac aging. The sample size, which was determined with the assumption of a matched biological sex in the two cohorts and a Cohen effect size of 3, can guarantee only detection of very large changes across analytes between the younger and older cohorts. However, the difficulty of obtaining young donor hearts in the modern medical era due to the increased availability of heart transplant centers, advanced immunosuppression techniques, and advancements in surgical transplant techniques highlights the uniqueness of the sample set used, with a replicative sample set unlikely to be repeated. Potential confounding factors due to lifestyle differences between younger and older individuals are another limitation of using human pre‐mortem samples. However, the criteria that must be followed for a donor heart to be considered for transplantation do help to mitigate some potential confounders. For example, regardless of whether young or old, significant comorbidities related to CV disease (diabetes, high BMI, hypertension, heavy smoking etc.) or any chronic illnesses were not present in any patients, as the hearts would then have not been considered for transplantation. Another possible confounding factor to consider is the differing menopausal status of the women in the older cohort. The average age of the older women in our study is 56, and we can assume that most of these donors would be in menopause. However, we do not have specific information regarding the menopausal status of each woman and therefore recognize this as a potential limitation. Regarding the type of samples able to be obtained, we are unable to analyze the extreme ends of aging, with our oldest donor being 65. Older patients would not have been suitable for heart transplantation and therefore do not fit the criteria for healthy hearts collected. However, it is important to consider the advantages of analyzing the early stages of cardiac aging. This can and will assist in our understanding of how we can prevent changes to the heart that will ultimately affect functions and are risk factors for heart failure. These key limitations regarding the number and type of human cardiac samples we are able to obtain may contribute to the low number of differentially expressed genes and proteins detected in the study. Therefore, while highly relevant molecular signatures of human cardiac aging have been uncovered, there may be genes and/or proteins that were not detected as differentially expressed in this study that are relevant to the aging process. Another limitation of the current study is that only whole myocardial level data is analyzed. However, the study undertaken here is the only possible way in which comparative investigations can be done on pre‐mortem cryopreserved normal human myocardium of different ages. As prospective fresh tissue acquisition is highly unlikely in the modern era, definitive attribution to cellular origin is extremely difficult. Another consideration is that while we have observed unique changes present in human cardiac aging, the methodology of the current study is not able to differentiate if these changes are causative in the aging process or a consequence of it. However, our biological modeling begins to address this point, as disrupting Ca^2+^ handling lends itself to a system of HF.

## 5 Conclusions

We present the first comprehensive resource of molecular changes underpinning normal human cardiac aging. This study leveraged unique pre‐mortem, cryopreserved human myocardium across the age span, paired with comprehensive multi‐omics investigation and biophysics‐based, physiology‐informed in silico modeling. We report several insights for the first time: a decrease in proteins composing the structural machinery of the heart; a likely compensatory increase in energetic flux and redox reductive capacity; and an increase in lysosomal activity and storage. The specific components of these processes, and the physiological consequences our modeling outlines, are instructive for strategies to develop potential therapeutic targets for age‐related CVDs, in addition to providing the first human cardiac aging resource (https://humancardiac.shinyapps.io/ageing/).

## Author Contributions

Cassandra Malecki jointly conceived the study, extracted RNA for transcriptomics, and wrote the manuscript. Giovanni Guglielmi jointly conceived the study, performed the statistical analyses, created the figures and the online interactive application, and wrote the manuscript. Benjamin Hunter retrieved the samples, processed the tissue, and extracted the samples for proteomics and metabolomics. Dylan Harney performed the proteomics. Yen Chin Koay designed and performed the metabolomics. Anthony. S. Don supervised and provided intellectual critique of the lipidomics data. Oscar Han assisted with initial study design and initial statistical analyses. Jasmine Khor extracted samples for lipidomics. Lisa Nguyen performed proof of concept experiments. Michael Pan, Peter Gawthrop, Nathan Isles, and Joshua Chung performed and analyzed the computation modeling. Robert. D. Hume and Matthew Taper performed immunofluorescence staining and confocal imaging. Xiao Suo Wang assisted with metabolomics analyses and interpretation. Mark Larance supervised and provided intellectual critique of the proteomics data. Fabian Spill jointly conceived the study, supervised and provided intellectual critique of all statistical analyses, oversaw data interpretation, and wrote the manuscript. Vijay Rajagopal jointly conceived the study, supervised, and provided intellectual critique of computer modeling, oversaw analysis and data interpretation, and wrote the manuscript. John F. O'Sullivan jointly conceived the study, oversaw the metabolomics analysis and data interpretation, and wrote the manuscript. Sean Lal conceived the original study design, oversaw the myocardial tissue retrieval, oversaw analysis and data interpretation, and wrote the manuscript.

## Conflicts of Interest

The authors declare no conflicts of interest.

## Supporting information


**Data S1.** acel70219‐sup‐0001‐DataS1.zip.

**Table S0.**The log transformed, normalised values of all samples for proteomics, metabolomics, lipidomics, and transcriptomics as well as data with all imputed values for each omics set. Additionally, the overlap of samples between each omics analysis is also presented.

**Table S1.**The clinical information for each donor sample used in the study

**Table S2.**Differential expression analysis results of younger versus older hearts for proteomics, metabolomics, lipidomics, and transcriptomics. Each analyte's abundance difference was tested using the two‐sided LIMMA t‐test on the normalised and log_2_ transformed data.

**Table S3.**Differential expression analysis of proteins between younger males versus older males and younger female's versus older females.

**Table S4.**Effect size comparison and related *p*‐values for the whole cohort, and gender stratified cohorts

**Table S5.** Weighted Correlation Network Analysis (WCNA) results and over‐representation analysis (ORA) results at the protein level. The ORA was performed on the most populated community presented in the WCNA.

**Table S6.** Weighted Correlation Network Analysis (WCNA) results and over‐representation analysis (ORA) results at the metabolite level. The ORA was performed on the most populated community presented in the WCNA.

**Table S7.** Set enrichment analysis results of younger versus older hearts at the lipid level.

**Table S8.** Weighted Correlation Network Analysis (WCNA) results and over‐representation analysis (ORA) results at the lipid level. The ORA was performed on the most populated community presented in the WCNA.

**Table S9.** Weighted Correlation Network Analysis (WCNA) results and over‐representation analysis (ORA) results at the transcript level. The ORA was performed on the most populated community presented in the WCNA.

**Table S10.** Set enrichment analysis results of old versus young hearts at the protein, metabolite and transcript level. The analysis was performed on the WikiPathways database.

**Table S11.** Results of network analyses done separately on the younger and older cohorts for the protein, metabolite, lipid, and transcript level as well the identification of significant functional modules in each omics set

**Table S12.** Correlation results between key differentially expressed proteins and their corresponding transcript levels.

**Table S13.** Results of correlation analysis between ceramide levels and oxidative stress markers measured in the proteomics, metabolomics and transcriptomics datasets.

## Data Availability

All data and omics results are available via an interactive online repository created using Shiny and R (https://humancardiac.shinyapps.io/ageing/) as a publicly available resource. The web app was created using Shiny on R 4.2.2. The mass spectrometry proteomics data have been deposited to the ProteomeXchange Consortium via the PRIDE partner repository with the dataset identifier PXD050610, Username: reviewer_pxd050610@ebi.ac.uk, Password: xqdgKe5f. The mass spectrometry metabolomics data are currently available on Metabolomics Workbench at https://doi.org/10.21228/M8N42X. The lipidomics data are hosted on Metabolomics Workbench at https://doi.org/10.21228/M8N42X. The transcriptomics data are deposited in a public repository and available on GEO at https://www.ncbi.nlm.nih.gov/geo/query/acc.cgi?acc=GSE263566. The analysis Differential, Heatmap and Principal Component Analysis, Set Enrichment Analysis, and Network Analysis has been performed in R 4.2.2; the ggplot2 package was used to produce the figures. The code is available at https://github.com/gguglielmi92/Human‐Cardiac‐Ageing.git. The oxidative phosphorylation model was implemented in Julia 1.7.2, and it is available at https://github.com/mic‐pan/ageing_heart_oxphos. The dyad and SERCA2 models were implemented in MATLAB R2017b and 2021a, respectively, and the source codes are respectively hosted on https://github.com/CellSMB/1D‐Dyad‐Model‐RyR‐IP3R.git and https://github.com/CellSMB/ageing_heart_ecc.git.
