# Supplementary material for: The Human Cardiac “Age‐OME”: Age‐Specific Changes in Myocardial Molecular Expression
Source: Aging Cell. 2025 Sep 7;24(11):e70219. doi: 10.1111/acel.70219 (PMC12610413; doi:10.1111/acel.70219)
Supplement: Supplementary file 1 — Data S1. acel70219‐sup‐0001‐DataS1.zip. [file ACEL-24-e70219-s011.zip › Item S1.pdf]

Computational model of oxidative phosphorylation

Contents

A Introduction **3**

B The bond graph approach **3**

B.1 Species 3

B.2 Reactions 4

B.3 Network topology 4

B.4 Energy transformation 5

C Model components **5**

C.1 Species 5

C.2 Mitochondrial membrane capacitance 6

C.3 Reactions 6

C.4 Model integration 18

D Parameter estimation **20**

D.1 Hydrogen buffering 20

D.2 Adenine nucleotide translocase 20

D.3 ATP synthase 21

E Initial conditions **21**

E.1 Energetic molecules 23

F Sensitivity analysis **23**

G Simulation protocol **24**

A Introduction

This note describes the oxidative phosphorylation model used to generate insights on the functional consequences of metabolic changes that were observed in our study. The in-silico framework used to analyse the implication of our findings is based on the bond graph approach; this approach ensures energy conservation [1, 2].

The in-silico model was based on a previously published rat model [3], although a few updates, described as follows, were required to incorporate some processes of interest. Firstly, creatine kinase reactions were added to the cytosol and intermembrane space to enable predictions of creatine buffering and transport. Secondly, the transport rate of the adenine nucleotide translocator (ANT) was adjusted to fit with measurements of the phosphocreatine to ATP ratio in cardiomyocytes [4]. Thirdly, initial conditions were updated to match those seen in physiological conditions. Finally, The mitochondrial volume (relative to cell volume) was updated to reflect those seen in humans.

In Appendix B, the bond graph framework is briefly introduced. In Appendix C, the model details are presented, while Appendix D discusses the parameters used. Next, Appendix E reviews the initial conditions employed in the model with a sensitivity anaylysis shown in Appendix F. Appendix G discusses the details regarding the simulations.

B The bond graph approach

This section introduces the bond graph framework, a technique that ensures that thermodynamically consistent description of any biophysical system. We outline the main concepts of bond graph modelling below; the reader is referred to Gawthrop and Pan [2] for further detail.

Two variables are required to track energy flow through a biochemical system, the chemical potential μ (J/mol) and the flux v (mol/s). The bond $\rightharpoonup$ is the fundamental unit of connection in a bond graph, and it carries both chemical potential and flux variables. The product of these is the power P (J/s), allowing the modelling of the flow of energy. We note that that most conventional models consider the flux, but not the chemical potential.

B.1 Species

Species are modelled as energy-storing **Ce** components in a bond graph. These components encode the equations

$$\dot{x}_i = v_i \tag{1}$$

$$c_i = x_i/V \tag{2}$$

$$\mu_i = \mu_i^0 + RT \log\left(c_i/c_i^0\right), \tag{3}$$

where:

$$x_i = \text{molar amount of species } i \text{ (mol)} \tag{4a}$$
$$v_i = \text{rate of change of species } i \text{ in time (mol/s)} \tag{4b}$$
$$c_i = \text{concentration of species } i \text{ (mol/L)} \tag{4c}$$
$$V = \text{volume of the compartment (L)} \tag{4d}$$
$$\mu_i = \text{chemical potential of species } i \text{ (J/mol)} \tag{4e}$$
$$\mu_i^0 = \text{reference chemical potential of species } i \text{ (J/mol)} \tag{4f}$$
$$c_i^0 = \text{reference concentration of species } i \text{ (mol/L)} \tag{4g}$$
$$R = \text{ideal gas constant} = 8.314 \text{ J/mol/K} \tag{4h}$$
$$T = \text{absolute temperature} \tag{4i}$$

B.2 Reactions

Reactions are modelled as energy-dissipating **Re** components. Assuming mass action kinetics, these encode the equations

$$v = \kappa_j \left[\exp\left(\frac{\mu_j^f}{RT}\right) - \exp\left(\frac{\mu_j^r}{RT}\right) \right] \tag{5}$$

where κ_j is the rate parameter of the reaction and μ_j^f and μ_j^r are the total chemical potentials of the reactants and products respectively. Frequently, κ_j and μ_j^f and μ_j^r will take on extreme values. For example, κ_j may be vanishingly small whereas μ_j^f and μ_j^r may be large to compensate. Calculating $\exp(\mu_j^f/(RT))$ and $\exp(\mu_j^r/(RT))$ prior to multiplying by κ_j may result in values too large or small for numerical solvers to handle. Therefore, for numerical stability, we reformulate the above equation as follows in our simulations:

$$v = \exp\left(\frac{\mu_j^a + \mu_j^f}{RT}\right) - \exp\left(\frac{\mu_j^a + \mu_j^r}{RT}\right) \tag{6}$$

where $\mu_j^a = RT \ln(\kappa_j)$ is a log-transformed parameter that can be seen as being related to the activation energy of the reaction. For more complex reactions where regulation is involved, μ_j^a can be expressed as a function of the chemical potentials of the regulators; we represent reactions as multi-port **R** components in this case.

B.3 Network topology

The network topology of a bond graph links the species and reactions, ensuring that the chemical potentials and fluxes are correct. There are two key junctions that impose the network topology in bond graphs, which we describe below:

- **0**-junctions are used when a species is involved in multiple reactions. A **0**-junction with n connected bonds enforces the following relationships:

$$\mu_1 = \mu_2 = \ldots = \mu_n \tag{7}$$
$$\sum_{i=1}^{n} d_i v_i = 0 \tag{8}$$

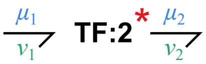

Figure A: Bond graph of a TF component.

where for the bond i, μ_i indicates the chemical potential, v_i denotes the flow and d_i is -1 if the bond points out, and 1 if the bond points in. Eq. 7 equates the chemical potential, since the same chemical potential is the same regardless of the reaction. Eq. 8 enforces mass conservation.

- **1**-junctions are used when a reaction has more than two reactants or products. A **1**-junction with n connected bonds enforces the following relationships

$$v_1 = v_2 = \ldots = v_n \tag{9}$$

$$\sum_{i=1}^{n} d_i \mu_i = 0 \tag{10}$$

  Eq. 9 enforces the common flux since all connected reactants/products are consumed/produced at the same rate. Eq. 10 ensures that the chemical potentials contributions of each of the reactants/products are summed.

B.4 Energy transformation

When multiple stoichiometries are involved, **TF** components are used. A **TF** component has two attached bonds (see Figure A), and the general equations are as follows:

$$\mu_2 = n\mu_1 \tag{11}$$

$$v_1 = nv_2 \tag{12}$$

where (μ_1, v_1) correspond to the incoming bond, (μ_2, v_2) correspond to the outgoing bond (indicated by the red asterisk) and n is the winding ratio (or stoichiometry). Eq. 11 ensures that multiples of the chemical potential are used, and Eq. 12 ensures that the reaction contributes multiple stoichiometry to the rate of accumulation of species.

TF components are also used when energy is transduced between different forms. In oxidative phosphorylation, conversion between chemical and electricity is of interest because of the movement of ions across a charged membrane. The conversion between these quantities is given by

$$\mu = FV \tag{13}$$

$$I = Fv \tag{14}$$

where V (Volts) is the electrial voltage, I (Amperes) is the current and $F = 96485$ Coulombs/mol is the Faraday constant.

C Model components

C.1 Species

The species in the model are listed in Table 1, along with their standard chemical potentials. The volumes of each compartment are summarised in Table 2. When fitting the model to rat data,

the rat mitochondrial volume was used. However, for simulations of the human heart, the human mitochondrial volume was used.

It has been observed that ATP production rates relative to tissue mass are lower in humans compared to rats [5–7]. We hypothesise that a lower mitochondrial density in humans [8] is a contributor to these differences in ATP production, although lower mitochondrial complex activity is also likely given the large differences in cardiac output between the two species [5]. To our knowledge, differences in complex activities between the two species have not been quantified. Hence, we only incorporated differences in mitochondrial density. Thus, our results likely overestimate the ATP production rates in human cardiomyocytes. However, we are mainly concerned with the relative changes to ATP production due to changes in metabolite and protein abundances with age, and we believe that those are robust to the baseline ATP production rate used.

C.2 Mitochondrial membrane capacitance

Because the mitochondrial membrane is electrically charged, the membrane voltage changes when ions are transported across. A linear relationship between membrane capacitance and charge was assumed for the mitochondrial matrix, consistent with previous studies [3]. This gives rise to the equations

$$\dot{q}_{\mathrm{im}} = I \tag{15}$$

$$\Delta\Psi = q_{\mathrm{im}}/C_{\mathrm{im}} \tag{16}$$

where q_{im} is the accumulated charge difference, I is the sum of the ionic currents, $\Delta\Psi$ is the mitochondrial membrane potential and C_{im} = 651.9 F/L mitochondria is the capacitance of the mitochondrial inner membrane [3]. These equations can be modelled by using a linear **C**:im component using bond graphs, analogous to an electrical capacitor. The membrane voltage $\Delta\Psi$ also influences the kinetics of the electrogenic transport processes; these are discussed in the relevant reactions in § C.3.

C.3 Reactions

We use the following notation to help with the interpretation of the reactions:

1. For ease of interpretion, some bonds will be labelled using chemical potential (blue) and flux (green) variables.
2. Several of the reactions are regulated by metabolites not involved in the reaction. In these cases, we will show the regulators using purple components.
3. For convenience, we define the functions $E(x) = \exp\left(\frac{x}{RT}\right)$ and $L(x) = RT\ln(x)$.

We list the parameters used for each reaction. Appendix D provides details on how the parameters were estimated.

C.3.1 Dehydrogenase reaction

$$\mathrm{NAD_x} \rightleftharpoons \mathrm{NADH_x} + \mathrm{H_x} \tag{17}$$

Table 1: Species included in the model of oxidative phosphorylation. The compartments are the cytosol (c), intermembrane space (i) and mitochondrial matrix (x). For magnesium, the ic compartment treats the cytosolic and intermembrane space compartments as the same volume, assuming that magnesium freely diffuses through the mitochondrial membrane [3, 9]. All standard free energies are taken at a standard concentration of 1M.

Species	Description	Compartments	μ^0 (kJ/mol)	Source
NAD	Nicotinamide adenine dinucleotide	x	18.10	[a]
NADH	Reduced NAD	x	39.31	[a]
Q	Ubiquinone	x	65.17	[a]
QH_2	Ubiquinol	x	−23.30	[a]
C_{ox}	Oxidised cytochrome c	i	−6.52	[a]
C_{red}	Reduced cytochrome c	i	−27.41	[a]
O_2	Oxygen gas	x	16.40	[a]
fADP	Free ADP	c,i,x	−1903.96	[a]
mADP	Mg-bound ADP	c,i,x	−2381.86	[d]
fATP	Free ATP	c,i,x	−2771.00	[a]
mATP	Mg-bound ATP	c,i,x	−3255.51	[e]
Pi	Inorganic phosphate	c,i,x	−1098.27	[a]
H	Hydrogen ion	c,i,x	0.00	[c]
B	Hydrogen buffer	x	0.00	[c]
HB	Hydrogen-buffer complex	x	−40.86	[g]
Mg	Magnesium	ic,x	−458.20	[b]
K	Potasssium ion	i,x	0.00	[c]
AMP	Adenosine monophosphate	c,i	−1034.66	[a]
Cr	Creatine	c,i	−252.68	[a]
PCr	Phosphocreatine	c,i	−1071.01	[f]

[a] Wu et al. [10], Table A3 of Supplementary Material.
[b] Taken from eQuilibrator [11], using an ionic strength of 0.17 M.
[c] Taken to be zero. The hydrogen ion H is in its standard state by convention [12], whereas the standard free energies for K and B are assumed to be in their standard forms for this model.
[d] Calculated using the equation $\mu^0_{\mathrm{mADP}} = \mu^0_{\mathrm{Mg}} - \mu^0_{\mathrm{fADP}} + RT\ln(K_{DD})$, where $K_{DD} = 3.47 \times 10^{-4}$ M is the dissociation constant of Mg from ADP.
[e] Calculated using the equation $\mu^0_{\mathrm{mATP}} = \mu^0_{\mathrm{Mg}} - \mu^0_{\mathrm{fATP}} + RT\ln(K_{DT})$, where $K_{DT} = 2.4 \times 10^{-5}$ M is the dissociation constant of Mg from ADP.
[f] Calculated using the relationship between standard free energies and equilibrium constant [13]. For the reaction $\mathrm{Cr} + \mathrm{mATP} \rightleftharpoons \mathrm{PCr} + \mathrm{ADP} + \mathrm{H}$, $\mu^0_{\mathrm{PCr}} = \mu^0_{\mathrm{Cr}} + \mu^0_{\mathrm{mATP}} - \mu^0_{\mathrm{ADP}} - \mu^0_{\mathrm{H}} + RT\ln(K^{\mathrm{eq}}_{\mathrm{CK}} K_{DD}/K_{DT})$, where $K^{\mathrm{eq}}_{\mathrm{CK}} = 3.57 \times 10^{8}\ \mathrm{M}^{-1}$ is the equilibrium constant of the creatine kinase reaction [10]. Because the Mg-bound forms of ADP are used, this equilibrium constant is adjusted by K_{DD} and K_{DT}.
[g] Calculated using the equation $\mu^0_{\mathrm{HB}} = \mu^0_{\mathrm{H}} - \mu^0_{\mathrm{B}} + RT\ln(K_d)$, where $K_d = 6.6892 \times 10^{-8}$ M is the dissociation constant (see § D.1)

Table 2: Volumes of each compartment. All volumes in units L water/L mitochondria (the volume of water in the compartment relative to the volume of the total mitochondrial volume). The volume of the cytosol corresponds to the volume relative to all mitochondria in the cell.

Compartment	Volume	Source
Cytosol (human)	2.1336	Barth et al. [8]
Cytosol (rat)	2.0194	Vinnakota and Bassingthwaighte [14]; Beard [3][a]
Intermembrane space	0.072376	Beard [3]
Matrix	0.651384	Beard [3]

[a] From the second column of Table 5 in Vinnakota and Bassingthwaighte [14], the cytosol has a 2.79 ratio of water volume relative to mitochondria. From Beard [3], the water fraction is 0.72376. Multiplying these together gives the water volume of 2.0194 in rats.

The reactions of the TCA cycle were lumped into a single phenomenological dehydrogenase reaction, shown by the bond graph in Figure B. The reaction follows the law of mass action, and is regulated by matrix phosphate. Phosphate control was included as it is required to explain experimental measurements on the mitochondrial membrane potential [13].

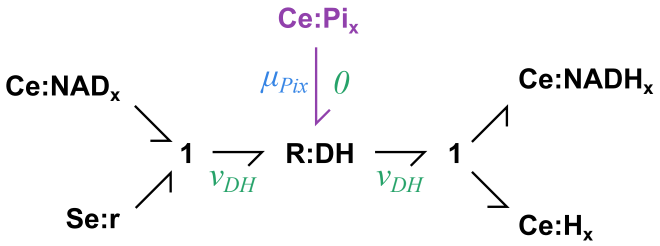

Figure B: Bond graph of the dehydrogenase reaction.

The rate law of the reaction is

$$v_{\mathrm{DH}} = r_{\mathrm{DH}}^{\mathrm{reg}} \left[E(\mu_{\mathrm{DH}}^a + \mu_r + \mu_{\mathrm{NAD_x}}) - E(\mu_{\mathrm{DH}}^a + \mu_{\mathrm{NADH_x}} + \mu_{\mathrm{H_x}}) \right] \tag{18}$$

$$r_{\mathrm{DH}}^{\mathrm{reg}} = \frac{1 + E(\mu_{\mathrm{Pi_x}} - \mu_{\mathrm{Pi1}}^{\mathrm{reg}})}{1 + E(\mu_{\mathrm{Pi_x}} - \mu_{\mathrm{Pi2}}^{\mathrm{reg}})} \tag{19}$$

$$\mu_{\mathrm{DH}}^a = -4.21 \ \mathrm{kJ/mol} \tag{20}$$

$$\mu_r = -16.03 \ \mathrm{kJ/mol} \tag{21}$$

$$\mu_{\mathrm{Pi1}}^{\mathrm{reg}} = -1120.32 \ \mathrm{kJ/mol} \tag{22}$$

$$\mu_{\mathrm{Pi2}}^{\mathrm{reg}} = -1116.32 \ \mathrm{kJ/mol} \tag{23}$$

$$\tag{24}$$

C.3.2 Complex I

$$\mathrm{NADH_x + Q + H_x \rightleftharpoons NAD_x + QH_2 + 4\Delta H} \tag{25}$$

The bond graph of Complex I is shown in Figure C.

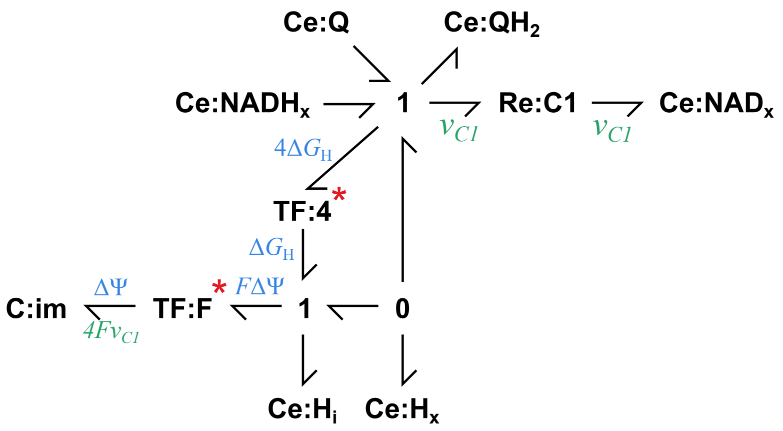

Figure C: Bond graph of Complex I.

From the bond graph, the reaction follows the rate law

$$v_{\mathrm{C1}} = E(\mu^a_{\mathrm{C1}} + \mu_{\mathrm{NADH_x}} + \mu_{\mathrm{Q}} - \mu_{\mathrm{QH_2}} + \mu_{\mathrm{H_x}} - 4\Delta G_H) - E(\mu^a_{\mathrm{C1}} + \mu_{\mathrm{NAD_x}}) \tag{26}$$

$$\Delta G_H = \mu_{\mathrm{H_i}} - \mu_{\mathrm{H_x}} + F\Delta\Psi \tag{27}$$

$$\mu^a_{\mathrm{C1}} = -21.01 \text{ kJ/mol} \tag{28}$$

C.3.3 Complex III

$$2\mathrm{C_{ox}} + \mathrm{QH_2} \rightleftharpoons 2\mathrm{C_{red}} + \mathrm{Q} + 2\mathrm{H_x} + 4\Delta\mathrm{H} \tag{29}$$

The bond graph of Complex III is shown in Figure D

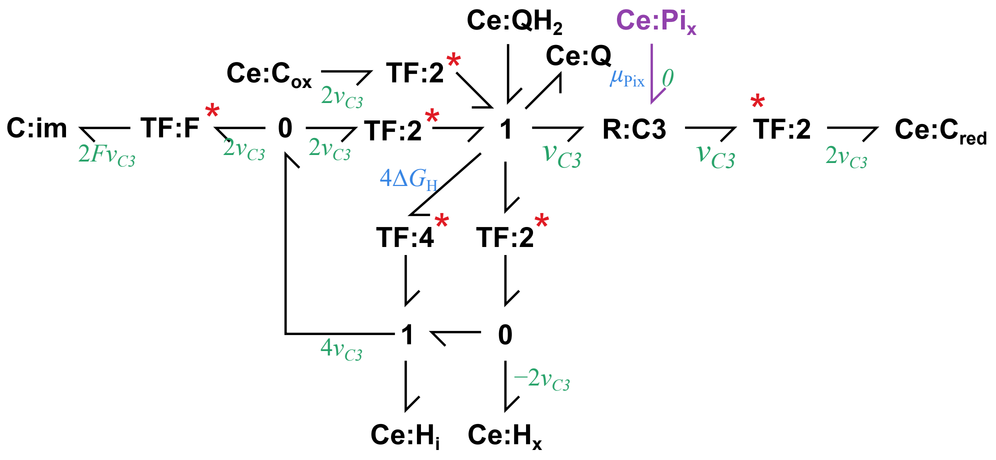

Figure D: Bond graph of Complex III.

The reaction component has a modified mass action rate law, with coefficient $\alpha = 2$ [15]. Matrix

phosphate is also a regulator of the reaction. The rate law is

$$v_{\mathrm{C3}} = r^{\mathrm{reg}}_{\mathrm{C3}} \left[E(\mu^{a}_{\mathrm{C3}} + (2\mu_{\mathrm{C_{ox}}} - 4\Delta G_{\mathrm{H}} - 2\mu_{\mathrm{H_x}} + 2F\Delta\Psi + \mu_{\mathrm{QH_2}} - \mu_{\mathrm{Q}})/\alpha) - E(\mu^{a}_{\mathrm{C3}} + 2\mu_{\mathrm{Cred}}/\alpha) \right] \tag{30}$$

$$r^{\mathrm{reg}}_{\mathrm{C3}} = \frac{1 + E(\mu_{\mathrm{Pi_x}} - \mu^{\mathrm{reg}}_{\mathrm{Pi3}})}{1 + E(\mu_{\mathrm{Pi_x}} - \mu^{\mathrm{reg}}_{\mathrm{Pi4}})} \tag{31}$$

$$\alpha = 2 \tag{32}$$

$$\mu^{a}_{\mathrm{C3}} = 21.24 \text{ kJ/mol} \tag{33}$$

$$\mu^{\mathrm{reg}}_{\mathrm{Pi3}} = -1119.44 \text{ kJ/mol} \tag{34}$$

$$\mu^{\mathrm{reg}}_{\mathrm{Pi4}} = -1107.36 \text{ kJ/mol} \tag{35}$$

C.3.4 Complex IV

$$2\mathrm{C_{red}} + 2\mathrm{H_2} + 0.5\mathrm{O_2} \rightleftharpoons 2\mathrm{C_{ox}} + 2\mathrm{H_2O} + 2\Delta\mathrm{H} \tag{36}$$

The bond graph of Complex IV is shown in Figure E.

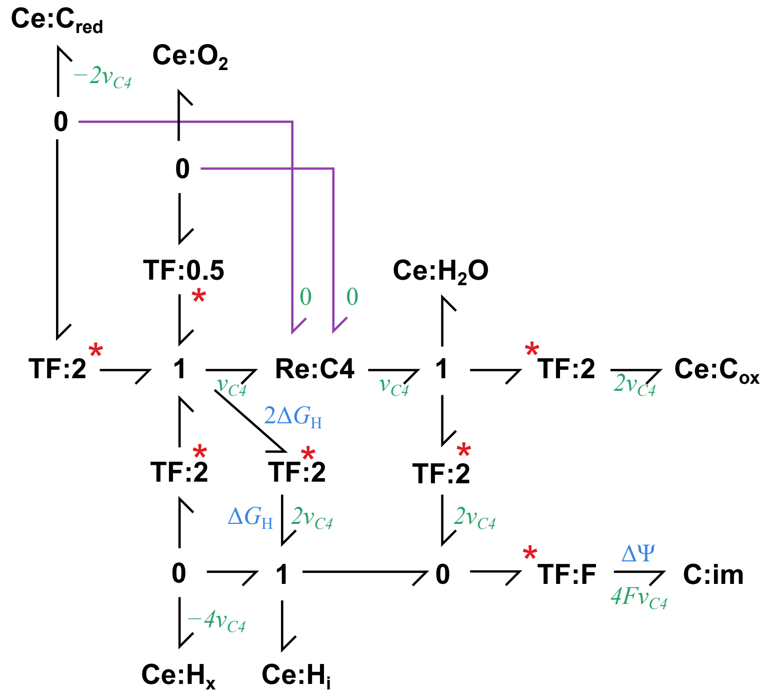

Figure E: Bond graph of Complex IV.

The reaction component has a modified mass action rate law, with coefficient $\alpha = 2$. Oxygen and

C_{red} are regulators of the reaction. The rate law is

$$v_{C4} = r_{C4}^{reg} [E(\mu_{C4}^{a} + (2\mu_{H_x} + 2\mu_{C_{red}} + 0.5\mu_{O_2} - 2\Delta G_H)/\alpha) \\ -E(\mu_{C4}^{a} + (2F\Delta\Psi + 2\mu_{C_{ox}} + \mu_{H_2O})/\alpha)] \quad (37)$$

$$r_{C4}^{reg} = \frac{E(\mu_{C_{red}} - \mu_{C_{red}}^{reg})}{1 + E(\mu_{O_2}^{reg} - \mu_{O_2})} \quad (38)$$

$$\alpha = 2 \quad (39)$$

$$\mu_{C4}^{a} = 99.09 \quad (40)$$

$$\mu_{C_{red}}^{reg} = -42.04 \text{ kJ/mol} \quad (41)$$

$$\mu_{O_2}^{reg} = -5.93 \text{ kJ/mol} \quad (42)$$

C.3.5 ATP synthase

$$\text{mADP}_x + \text{Pi}_x + \text{H}_x + 3\Delta G_H \rightleftharpoons \text{mATP}_x + \text{H}_2\text{O} \quad (43)$$

The bond graph of the ATP synthase is shown in Figure F. In line with the Beard model, we have assumed that 3 protons are required to synthesise an ATP molecule. However, we note that this value may vary slightly between models [9].

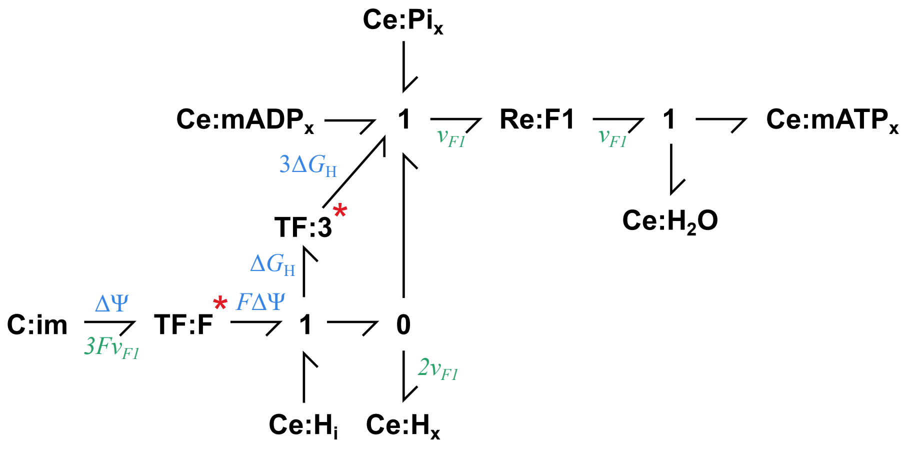

Figure F: Bond graph of ATP synthase.

The reaction is modelled using a mass action rate law, and has the equation

$$v_{F1} = E(\mu_{F1}^{a} + \mu_{mADP_x} + \mu_{Pi_x} + \mu_{H_x} + 3\Delta G_H) - E(\mu_{F1}^{a} + \mu_{mATP_x} + \mu_{H_2O}) \quad (44)$$

$$\mu_{F1}^{a} = 3504.12 \text{ kJ/mol} \quad (45)$$

C.3.6 Adenine nucleotide translocase (ANT)

$$\text{fADP}_i + \text{fATP}_x \rightleftharpoons \text{fADP}_x + \text{fATP}_i \quad (46)$$

The bond graph of the ANT is shown in Figure G. The reaction has a rate law based on the empirical equation from the Beard model, and is regulated by ADP in the intermembrane space.

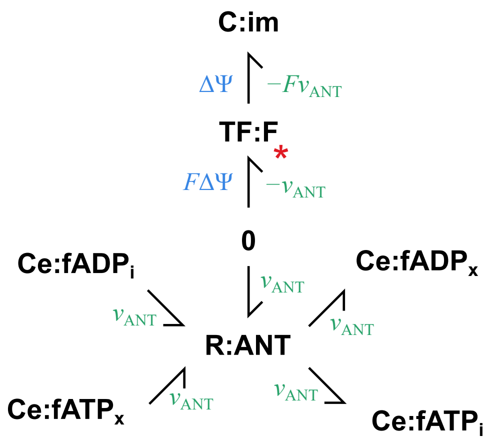

Figure G: Bond graph of ANT.

The rate law is given by the equation

$$v_{\mathrm{ANT}} = r_{\mathrm{ANT}}^{\mathrm{reg}} E(\mu_{\mathrm{ANT}}^{a}) \left[\frac{1}{1 + E(\mu_{\mathrm{fATP_i}} - \mu_{\mathrm{fADP_i}} - \mu_{\mathrm{ANT}}^{b} - pF\Delta\Psi)} - \frac{1}{1 + E(\mu_{\mathrm{fATP_x}} - \mu_{\mathrm{fADP_x}} - \mu_{\mathrm{ANT}}^{b} - (p-1)F\Delta\Psi} \right] \quad (47)$$

$$r_{\mathrm{ANT}}^{\mathrm{reg}} = \frac{1}{1 + E(\mu_{\mathrm{ANT}}^{\mathrm{reg}} - \mu_{\mathrm{ADPfi}})} \quad (48)$$

$$\mu_{\mathrm{ANT}}^{a} = -8.12 \text{ kJ/mol} \quad (49)$$

$$p = 0.35 \quad (50)$$

$$\mu_{\mathrm{ANT}}^{b} = \mu_{\mathrm{fATP}}^{0} - \mu_{\mathrm{fADP}}^{0} \quad (51)$$

$$\mu_{\mathrm{ANT}}^{\mathrm{reg}} = -1935.03 \text{ kJ/mol} \quad (52)$$

It can be shown that $v_{\mathrm{ANT}} \cdot (\mu_{\mathrm{fATP_i}} - \mu_{\mathrm{fADP_i}} - \mu_{\mathrm{fATP_x}} - \mu_{\mathrm{fADP_x}} + F\Delta\Psi) \geq 0$, hence the reaction component is dissipative.

C.3.7 Mg^{2+} binding to ATP and ADP

Many of the reactions involving ATP and ADP use their Mg-bound forms. The reactions for magnesium binding are modelled using the bond graphs in Figure H.

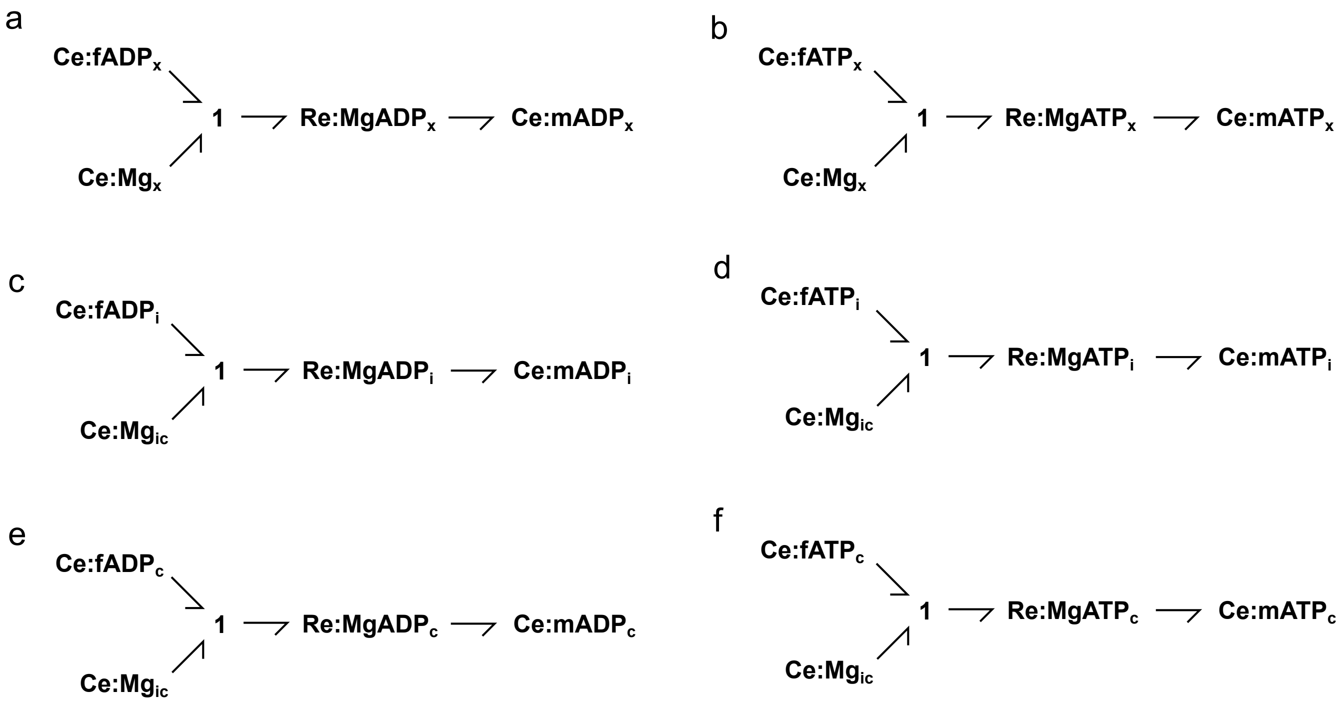

Figure H: Bond graphs of the reactions associated with Mg binding. Mg binding to (a) ADP in the matrix; (b) ATP in the matrix; (c) ADP in the intermembrane space; (d) ATP in the intermembrane space; (e) ADP in the cytosol; (f) ATP in the cytosol.

These reactions follow the law of mass action, given below.

$$v_{\mathrm{MgADP_x}} = E(\mu^a_{\mathrm{MgADP}} + \mu_{\mathrm{fADP_x}} + \mu_{\mathrm{Mg_x}}) - E(\mu^a_{\mathrm{MgADP}} + \mu_{\mathrm{mADP_x}}) \tag{53}$$

$$v_{\mathrm{MgATP_x}} = E(\mu^a_{\mathrm{MgATP}} + \mu_{\mathrm{fATP_x}} + \mu_{\mathrm{Mg_x}}) - E(\mu^a_{\mathrm{MgATP}} + \mu_{\mathrm{mATP_x}}) \tag{54}$$

$$v_{\mathrm{MgADP_i}} = E(\mu^a_{\mathrm{MgADP}} + \mu_{\mathrm{fADP_i}} + \mu_{\mathrm{Mg_{ic}}}) - E(\mu^a_{\mathrm{MgADP}} + \mu_{\mathrm{mADP_i}}) \tag{55}$$

$$v_{\mathrm{MgATP_i}} = E(\mu^a_{\mathrm{MgATP}} + \mu_{\mathrm{fATP_i}} + \mu_{\mathrm{Mg_{ic}}}) - E(\mu^a_{\mathrm{MgATP}} + \mu_{\mathrm{mATP_i}}) \tag{56}$$

$$v_{\mathrm{MgADP_c}} = E(\mu^a_{\mathrm{MgADP}} + \mu_{\mathrm{fADP_c}} + \mu_{\mathrm{Mg_{ic}}}) - E(\mu^a_{\mathrm{MgADP}} + \mu_{\mathrm{mADP_c}}) \tag{57}$$

$$v_{\mathrm{MgATP_c}} = E(\mu^a_{\mathrm{MgATP}} + \mu_{\mathrm{fATP_c}} + \mu_{\mathrm{Mg_{ic}}}) - E(\mu^a_{\mathrm{MgATP}} + \mu_{\mathrm{mATP_c}}) \tag{58}$$

$$\mu^a_{\mathrm{MgADP}} = 2396.33 \text{ kJ/mol} \tag{59}$$

$$\mu^a_{\mathrm{MgATP}} = 3263.37 \text{ kJ/mol} \tag{60}$$

The parameters μ^a_{MgADP} and μ^a_{MgATP} were chosen to be high so that these reactions would effectively be in rapid equilibrium.

C.3.8 Proton leak

$$\mathrm{H_i} \rightleftharpoons \mathrm{H_x} \tag{61}$$

The bond graph of proton leak is shown in Figure I.

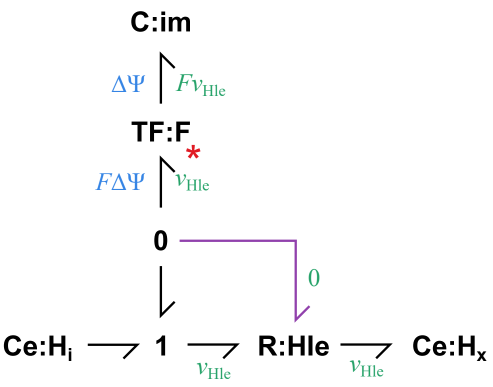

Figure I: Bond graph of Complex Hle.

The reaction follows a mass action rate law, but is modulated by the mitochondrial membrane potential according to the Nernst-Goldman equation. The rate law is

$$v\mathrm{Hle} = r_{\mathrm{Hle}}^{\mathrm{reg}}\left[E(\mu_{\mathrm{Hle}}^{a} + \mu_{\mathrm{Hi}} + F\Delta\Psi) - E(\mu_{\mathrm{Hle}}^{a} + \mu_{\mathrm{Hx}} + F\Delta\Psi)\right] \tag{62}$$

$$r_{\mathrm{Hle}}^{\mathrm{reg}} = \frac{\Delta\Psi}{E(F\Delta\Psi) - 1} \tag{63}$$

$$\mu_{\mathrm{Hle}}^{a} = 13.66 \text{ kJ/mol} \tag{64}$$

C.3.9 K⁺/H⁺ antiport

$$\mathrm{H_i} + \mathrm{K_x} \rightleftharpoons \mathrm{H_x} + \mathrm{K_i} \tag{65}$$

The bond graph of K^+/H^+ antiports is shown in Figure J.

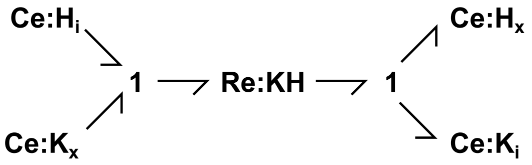

Figure J: Bond graph of K⁺/H⁺ antiport.

The reaction is modelled using a mass action rate law, and has the equation

$$v_{\mathrm{KH}} = E(\mu_{\mathrm{KH}}^{a} + \mu_{\mathrm{H_i}} + \mu_{\mathrm{K_x}}) - E(\mu_{\mathrm{KH}}^{a} + \mu_{\mathrm{H_x}} + \mu_{\mathrm{K_i}}) \tag{66}$$

$$\mu_{\mathrm{KH}}^{a} = 42.57 \text{ kJ/mol} \tag{67}$$

C.3.10 Substrate transport

All forms of ATP, ADP, AMP, Pi, Cr and PCr are able to diffuse through the outer mitochondrial membrane. The reactions for these transport reactions are given in Figure K.

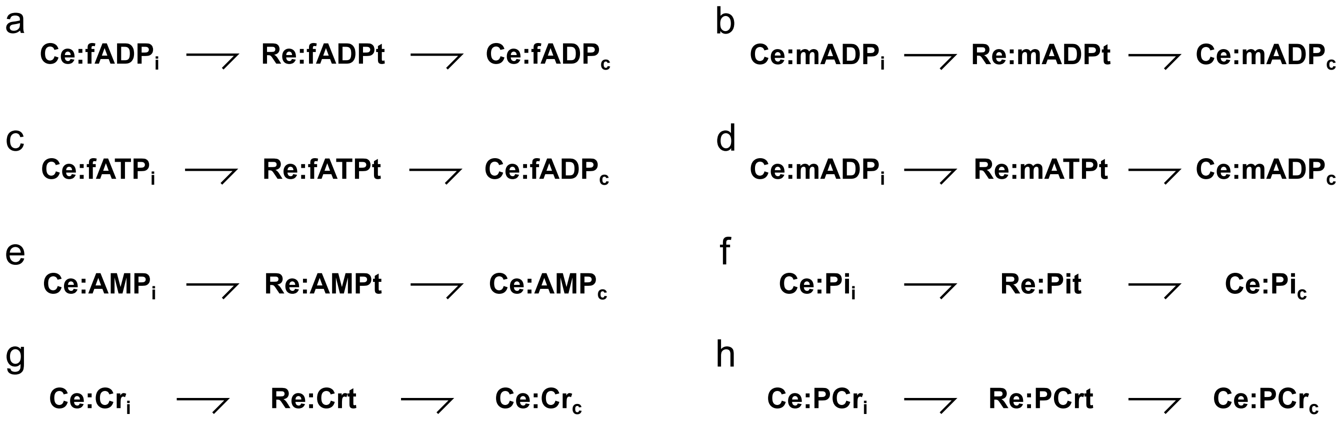

Figure K: Bond graphs of the substrate transport reactions between the intermembrane space and cytosol. (a) fADP; (b) mADP; (c) fATP; (d) mADP; (e) AMP; (f) Pi; (g) Cr; (h) PCr.

These reactions follow the law of mass action, given below.

$$v_{\mathrm{fADPt}} = E(\mu^a_{\mathrm{fADPt}} + \mu_{\mathrm{fADP_c}}) - E(\mu^a_{\mathrm{fADPt}} + \mu_{\mathrm{fADP_i}}) \tag{68}$$
$$v_{\mathrm{mADPt}} = E(\mu^a_{\mathrm{mADPt}} + \mu_{\mathrm{mADP_c}}) - E(\mu^a_{\mathrm{mADPt}} + \mu_{\mathrm{mADP_i}}) \tag{69}$$
$$v_{\mathrm{fATPt}} = E(\mu^a_{\mathrm{fATPt}} + \mu_{\mathrm{fATP_c}}) - E(\mu^a_{\mathrm{fATPt}} + \mu_{\mathrm{fATP_i}}) \tag{70}$$
$$v_{\mathrm{mATPt}} = E(\mu^a_{\mathrm{mATPt}} + \mu_{\mathrm{mATP_c}}) - E(\mu^a_{\mathrm{mATPt}} + \mu_{\mathrm{mATP_i}}) \tag{71}$$
$$v_{\mathrm{AMPt}} = E(\mu^a_{\mathrm{AMPt}} + \mu_{\mathrm{AMP_c}}) - E(\mu^a_{\mathrm{AMPt}} + \mu_{\mathrm{AMP_i}}) \tag{72}$$
$$v_{\mathrm{Pit}} = E(\mu^a_{\mathrm{Pit}} + \mu_{\mathrm{Pi_c}}) - E(\mu^a_{\mathrm{Pit}} + \mu_{\mathrm{Pi_i}}) \tag{73}$$
$$v_{\mathrm{Crt}} = E(\mu^a_{\mathrm{Crt}} + \mu_{\mathrm{Cr_c}}) - E(\mu^a_{\mathrm{Crt}} + \mu_{\mathrm{Cr_i}}) \tag{74}$$
$$v_{\mathrm{PCrt}} = E(\mu^a_{\mathrm{PCrt}} + \mu_{\mathrm{PCr_c}}) - E(\mu^a_{\mathrm{PCrt}} + \mu_{\mathrm{PCr_i}}) \tag{75}$$
$$\mu^a_{\mathrm{fADPt}} = 1919.38 \text{ kJ/mol} \tag{76}$$
$$\mu^a_{\mathrm{mADPt}} = 2397.28 \text{ kJ/mol} \tag{77}$$
$$\mu^a_{\mathrm{fATPt}} = 2786.42 \text{ kJ/mol} \tag{78}$$
$$\mu^a_{\mathrm{mATPt}} = 3270.93 \text{ kJ/mol} \tag{79}$$
$$\mu^a_{\mathrm{AMPt}} = 1050.08 \text{ kJ/mol} \tag{80}$$
$$\mu^a_{\mathrm{Pit}} = 1117.02 \text{ kJ/mol} \tag{81}$$
$$\mu^a_{\mathrm{Crt}} = 266.79 \text{ kJ/mol} \tag{82}$$
$$\mu^a_{\mathrm{PCrt}} = 1085.12 \text{ kJ/mol} \tag{83}$$

C.3.11 Hydrogen buffering

$$\mathrm{H_x} + \mathrm{B_x} \rightleftharpoons \mathrm{HB_x} \tag{84}$$

A hydrogen buffering reaction was modelled using a simple mass action reaction assumed to be at equilibrium. The bond graph of the reaction is shown in Figure L.

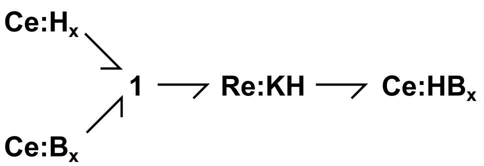

Figure L: Bond graph of hydrogen buffering.

$$v_{\mathrm{Hbuff}} = E(\mu^a_{\mathrm{Hbuff}} + \mu_{\mathrm{H_x}} + \mu_{\mathrm{B_x}}) - E(\mu^a_{\mathrm{Hbuff}} + \mu_{\mathrm{HB_x}}) \tag{85}$$
$$\mu^a_{\mathrm{Hbuff}} = 51.26\ \mathrm{kJ/mol} \tag{86}$$

C.3.12 Adenylate kinase

The adenylate kinase enzyme catalyses the conversion of two ADP molecules into ATP and AMP. The reaction occurs in both the cytosol and intermembrane space, and the corresponsing bond graph are given in Figure M.

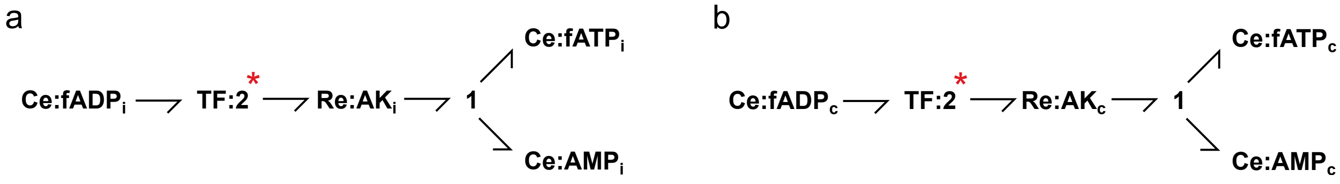

Figure M: Bond graphs of the reactions associated with adenylate kinase. (a) Cytosolic reaction; (b) Intermembrane space reaction.

These reactions follow the law of mass action, given below.

$$v_{\mathrm{AK_i}} = E(\mu^a_{\mathrm{AK}} + 2\mu_{\mathrm{fADP_i}}) - E(\mu^a_{\mathrm{AK}} + \mu_{\mathrm{fATP_i}} + \mu_{\mathrm{AMP_i}}) \tag{87}$$
$$v_{\mathrm{AK_c}} = E(\mu^a_{\mathrm{AK}} + 2\mu_{\mathrm{fADP_c}}) - E(\mu^a_{\mathrm{AK}} + \mu_{\mathrm{fATP_c}} + \mu_{\mathrm{AMP_c}}) \tag{88}$$
$$\mu^a_{\mathrm{AK}} = 3842.09\ \mathrm{kJ/mol} \tag{89}$$

The parameter μ^a_{AK} was chosen to be high so that these reactions would effectively be in rapid equilibrium.

Compared to the Beard model, an additional adenylate kinase reaction was added in the cytosolic compartment (Figure M(a)). The reaction was assumed to be at rapid equilibrium, with the same reaction parameter as that in the intermembrane space.

C.3.13 Phosphate transporter

$$\mathrm{Pi_i} + 2\mathrm{H_i} \rightleftharpoons \mathrm{Pi_x} + 2\mathrm{H_x} \tag{90}$$

The bond graph of the phosphate transporter is shown in Figure N.

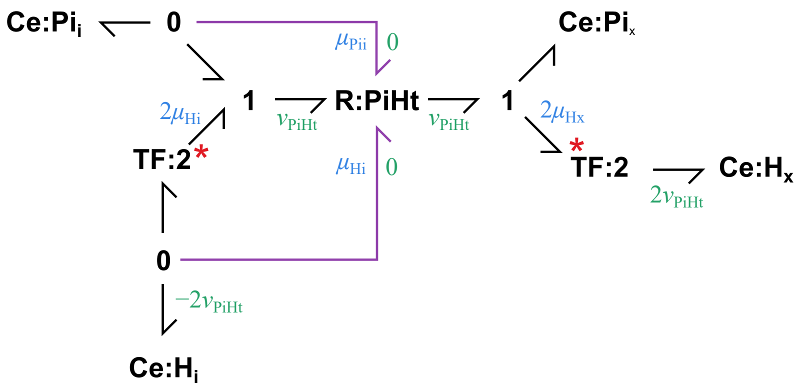

Figure N: Bond graph of the phosphate transporter.

To model Michaelis-Menten-like kinetics, the reaction is modulated by both $\mathrm{Pi_i}$ and $\mathrm{H_i}$. The corresponding rate law is

$$v_{\mathrm{PiHt}} = r_{\mathrm{PiHt}}^{\mathrm{reg}} \left[E(\mu_{\mathrm{PiHt}}^{a} + \mu_{\mathrm{Pi_i}} + 2\mu_{\mathrm{H_i}}) - E(\mu_{\mathrm{PiHt}}^{a} + \mu_{\mathrm{Pi_x}} + 2\mu_{\mathrm{H_x}})\right] \quad (91)$$

$$r_{\mathrm{PiHt}}^{\mathrm{reg}} = \left[\left(1 + \frac{E(\mu_{\mathrm{H_i}} - \mu_{\mathrm{HPi}}^{d} + \mu_{\mathrm{Pi_i}} - \mu_{\mathrm{PiHt}}^{\mathrm{reg}})}{(1 + E(\mu_{\mathrm{H_i}} - \mu_{\mathrm{HPi}}^{d}))}\right)(1 + E(\mu_{\mathrm{H_i}} - \mu_{\mathrm{HPi}}^{d})\right]^{-1} \quad (92)$$

$$\mu_{\mathrm{PiHt}}^{a} = -1117.33 \text{ kJ/mol} \quad (93)$$

$$\mu_{\mathrm{HPi}}^{d} = -38.44 \text{ kJ/mol} \quad (94)$$

$$\mu_{\mathrm{PiHt}}^{\mathrm{reg}} = 1187.27 \text{ kJ/mol} \quad (95)$$

C.3.14 Creatine kinase

Creatine kinase reactions were included in the intermembrane space and cytosol. The bond graphs of these reactions are shown in Figure O.

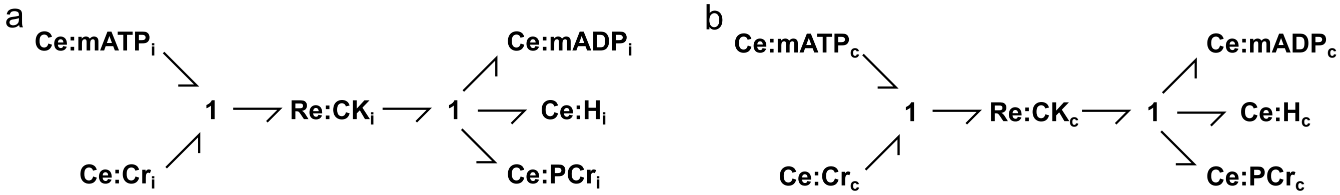

Figure O: Bond graph of the creatine kinase reactions. (a) Intermembrane space; (b) Cytosol.

These reactions follow the law of mass action, and reported as follows.

$$v_{\mathrm{CK_i}} = E(\mu_{\mathrm{CK_i}}^{a} + \mu_{\mathrm{mATP_i}} + \mu_{\mathrm{Cr_i}}) - E(\mu_{\mathrm{CK_i}}^{a} + \mu_{\mathrm{mADP_i}} + \mu_{\mathrm{PCr_i}} + \mu_{\mathrm{H_i}}) \quad (96)$$

$$v_{\mathrm{CK_c}} = E(\mu_{\mathrm{CK_c}}^{a} + \mu_{\mathrm{mATP_c}} + \mu_{\mathrm{Cr_c}}) - E(\mu_{\mathrm{CK_c}}^{a} + \mu_{\mathrm{mADP_c}} + \mu_{\mathrm{PCr_c}} + \mu_{\mathrm{H_c}}) \quad (97)$$

$$\mu_{\mathrm{CK_i}}^{a} = 3533.99 \text{ kJ/mol} \quad (98)$$

$$\mu_{\mathrm{CK_c}}^{a} = 3542.36 \text{ kJ/mol} \quad (99)$$

The parameters $\mu_{\mathrm{CK_i}}^{a}$ and $\mu_{\mathrm{CK_c}}^{a}$ were chosen to be high so that these reactions would effectively be in rapid equilibrium.

C.3.15 ATP consumption

$$\mathrm{mATP_c} + \mathrm{H_2O} \rightleftharpoons \mathrm{mADP_c} + \mathrm{Pi_c} + \mathrm{H_c} \tag{100}$$

A phenomenologial reaction was used to model the consumption of ATP by cellular processes. The bond graph of the reaction is shown in Figure P.

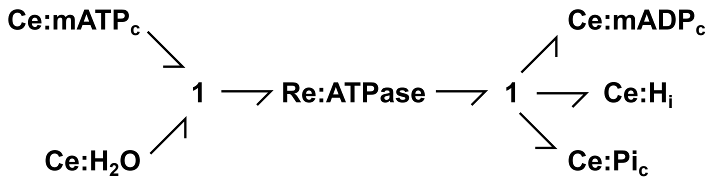

Figure P: Bond graph of ATP consumption.

The reaction is modelled using a mass action rate law, and has the equation

$$v_{\mathrm{ATPase}} = E(\mu^a_{\mathrm{ATPase}} + \mu_{\mathrm{mATP_c}} + \mu_{\mathrm{H_2O}}) - E(\mu^a_{\mathrm{ATPase}} + \mu_{\mathrm{mADP_c}} + \mu_{\mathrm{Pi_c}} + \mu_{\mathrm{H_i}}) \tag{101}$$

$$\mu^a_{\mathrm{ATPase}} = 3487.09 \text{ kJ/mol} \tag{102}$$

An initial estimate of $\mu^a_{\mathrm{ATPase}} = 3487.09$ kJ/mol was determined by fitting to simulation results from the Beard model. However, the energy demand of a cardiomyocyte can change depending on the heart rate [16]. Hence, the parameter μ^a_{ATPase} was varied from 3481.39 to 3491.90 kJ/mol in generating the results to simulate the effect of varying workload from a baseline level to high workload. We note that range of simulated workloads (Fig. 6g–h of the Main Text) covers workloads observed in human cardiomyocytes (121 pmol $O_2 \cdot s^{-1} \cdot (\text{mg tissue})^{-1}$, which corresponds to 6.05×10^{-4} mol $O_2 \cdot (\text{L mito water})^{-1} \cdot s^{-1}$) [6].

C.4 Model integration

The bond graphs in figures B–P were coupled together by using the white-box approach [2, 17]. Briefly, common **Ce** components between modules are merged into a single component connected to a 0-junction to enforce mass conservation. Additionally, the species H_i, H_c O_2 and K_i are modelled as chemostats, i.e. they have fixed concentrations.

The resulting mass balance equations are below:

$$\dot{q}_{\text{im}} = F(4v_{\text{C1}} + 2v_{\text{C3}} + 4v_{\text{C4}} - v_{\text{ANT}} - v_{\text{Hle}} - 3v_{\text{F1}}) \tag{103}$$

$$\dot{x}_{\text{NADH}_\text{x}} = v_{\text{DH}} - v_{\text{C1}} \tag{104}$$

$$\dot{x}_{\text{NAD}_\text{x}} = -v_{\text{DH}} + v_{\text{C1}} \tag{105}$$

$$\dot{x}_{\text{Q}} = -v_{\text{C1}} + v_{\text{C3}} \tag{106}$$

$$\dot{x}_{\text{QH}_2} = v_{\text{C1}} - v_{\text{C3}} \tag{107}$$

$$\dot{x}_{\text{H}_\text{x}} = 2v_{\text{F1}} + 2v_{\text{PiHt}} + v_{\text{DH}} + v_{\text{Hle}} - 5v_{\text{C1}} - v_{\text{KH}} - 2v_{\text{C3}} - 4v_{\text{C4}} - v_{\text{Hbuff}} \tag{108}$$

$$\dot{x}_{\text{B}_\text{x}} = -v_{\text{Hbuff}} \tag{109}$$

$$\dot{x}_{\text{HB}_\text{x}} = v_{\text{Hbuff}} \tag{110}$$

$$\dot{x}_{\text{C}_\text{ox}} = -2v_{\text{C3}} + 2v_{\text{C4}} \tag{111}$$

$$\dot{x}_{\text{C}_\text{red}} = 2v_{\text{C3}} - 2v_{\text{C4}} \tag{112}$$

$$\dot{x}_{\text{fATP}_\text{x}} = -v_{\text{ANT}} - v_{\text{MgATP}_\text{x}} \tag{113}$$

$$\dot{x}_{\text{mATP}_\text{x}} = v_{\text{F1}} + v_{\text{MgATP}_\text{x}} \tag{114}$$

$$\dot{x}_{\text{fATP}_\text{i}} = v_{\text{AK}_\text{i}} + v_{\text{ANT}} + v_{\text{fATP}} - v_{\text{MgATPi}} \tag{115}$$

$$\dot{x}_{\text{fADP}_\text{x}} = v_{\text{ANT}} - v_{\text{MgADP}_\text{x}} \tag{116}$$

$$\dot{x}_{\text{mADP}_\text{x}} = v_{\text{MgADP}_\text{x}} - v_{\text{F1}} \tag{117}$$

$$\dot{x}_{\text{fADP}_\text{i}} = v_{\text{fADP}} - 2v_{\text{AK}_\text{i}} - v_{\text{ANT}} - v_{\text{MgADPi}} \tag{118}$$

$$\dot{x}_{\text{Pi}_\text{x}} = v_{\text{PiHt}} - v_{\text{F1}} \tag{119}$$

$$\dot{x}_{\text{Pi}_\text{i}} = v_{\text{Pit}} - v_{\text{PiHt}} \tag{120}$$

$$\dot{x}_{\text{Mg}_\text{x}} = -v_{\text{MgADP}_\text{x}} - v_{\text{MgATP}_\text{x}} \tag{121}$$

$$\dot{x}_{\text{K}_\text{x}} = v_{\text{K}} + v_{\text{KH}} \tag{122}$$

$$\dot{x}_{\text{AMP}_\text{i}} = v_{\text{AK}_\text{i}} + v_{\text{AMP}} \tag{123}$$

$$\dot{x}_{\text{mATP}_\text{i}} = v_{\text{MgATPi}} + v_{\text{mATP}} - v_{\text{CK}_\text{i}} \tag{124}$$

$$\dot{x}_{\text{mADP}_\text{i}} = v_{\text{MgADPi}} + v_{\text{mADP}} + v_{\text{CK}_\text{i}} \tag{125}$$

$$\dot{x}_{\text{Mg}_\text{ic}} = -v_{\text{MgATPe}} - v_{\text{MgADPe}} - v_{\text{MgATPi}} - v_{\text{MgADPi}} \tag{126}$$

$$\dot{x}_{\text{mATP}_\text{c}} = -v_{\text{mATP}} + v_{\text{MgATPe}} - v_{\text{ATPase}} - v_{\text{CK}_\text{c}} \tag{127}$$

$$\dot{x}_{\text{fATP}_\text{c}} = -v_{\text{fATP}} - v_{\text{MgATPe}} + v_{\text{AK}_\text{c}} \tag{128}$$

$$\dot{x}_{\text{mADP}_\text{c}} = -v_{\text{mADP}} + v_{\text{MgADPe}} + v_{\text{ATPase}} + v_{\text{CK}_\text{c}} \tag{129}$$

$$\dot{x}_{\text{fADP}_\text{c}} = -v_{\text{fADP}} - v_{\text{MgADPe}} - 2v_{\text{AK}_\text{c}} \tag{130}$$

$$\dot{x}_{\text{AMP}_\text{c}} = -v_{\text{AMP}} + v_{\text{AK}_\text{c}} \tag{131}$$

$$\dot{x}_{\text{Pi}_\text{c}} = -v_{\text{Pit}} + v_{\text{ATPase}} \tag{132}$$

$$\dot{x}_{\text{Cr}_\text{c}} = -v_{\text{CK}_\text{c}} - v_{\text{Crt}} \tag{133}$$

$$\dot{x}_{\text{PCr}_\text{c}} = v_{\text{CK}_\text{c}} - v_{\text{PCrt}} \tag{134}$$

$$\dot{x}_{\text{Cr}_\text{i}} = -v_{\text{CK}_\text{i}} + v_{\text{Crt}} \tag{135}$$

$$\dot{x}_{\text{PCr}_\text{i}} = v_{\text{CK}_\text{i}} + v_{\text{PCrt}} \tag{136}$$

D Parameter estimation

Where possible, the reaction parameters were determined using the kinetic parameters from the Beard model. Because the rate laws of bond graph models lead to mathematical equations similar to Beard, the parameters were determined by equating the forward rate coefficients where possible. For example, in the case of K^+/H^+ antiport, the rate law is

$$v_{\mathrm{KH}} = E(\mu^a_{\mathrm{KH}} + \mu_{\mathrm{H_i}} + \mu_{\mathrm{K_x}}) - E(\mu^a_{\mathrm{KH}} + \mu_{\mathrm{H_x}} + \mu_{\mathrm{K_i}}) \tag{137}$$

$$= E(\mu^a_{\mathrm{KH}} + \mu^0_{\mathrm{H_i}} + \mu^0_{\mathrm{K_x}})c_{\mathrm{H_i}}c_{\mathrm{K_x}} - E(\mu^a_{\mathrm{KH}} + \mu^0_{\mathrm{H_x}} + \mu^0_{\mathrm{K_i}})c_{\mathrm{H_x}}c_{\mathrm{K_i}}, \tag{138}$$

which follows the law of mass action. This can be equated to the forward rate constant of the Beard model (X_{KH}) through the equation

$$E(\mu^a_{\mathrm{KH}} + \mu^0_{\mathrm{H_i}} + \mu^0_{\mathrm{K_x}}) = X_{\mathrm{KH}} \quad \Rightarrow \quad \mu^a_{\mathrm{KH}} = L(X_{\mathrm{KH}}) - \mu^0_{\mathrm{H_i}} - \mu^0_{\mathrm{K_x}} \tag{139}$$

The other reaction parameters (where present in the Beard model) were calculated similarly, with exceptions and additional constraints discussed in the sections below.

D.1 Hydrogen buffering

The Beard model used a phenomenological linear buffering capacitance term for hydrogen. This equation fails to capture the conservation of mass in buffered hydrogen, so we replaced this with a simple mass action model of hydrogen buffering $\mathrm{H} + \mathrm{B} \rightleftharpoons \mathrm{HB}$, assumed to be at quasi-equilibrium. Under these conditions, it can be shown that

$$\dot{x}_{\mathrm{H_x}} = b \sum v \tag{140}$$

$$b = \frac{1}{1 + B_t K/(c_{\mathrm{H}} + K)^2} \tag{141}$$

where $\sum v$ is the sum of fluxes producing/consuming hydrogen, $B_t = c_{\mathrm{B}} + c_{\mathrm{HB}}$ is the total buffer concentration and $K = E(\mu^0_{\mathrm{HB}} - \mu^0_{\mathrm{H}} - \mu^0_{\mathrm{H}})$ is the dissociation constant of the reaction [18]. The factor b was fitted to the term $100x_{\mathrm{H_x}}$ term used in the Beard model, in the range pH 7.0 to pH 7.4. Nonlinear least squares fitting was used, resulting in the parameters $B_t = 0.04071$ M, $K = 6.6892 \times 10^{-8}$ M.

D.2 Adenine nucleotide translocase

To ensure physiological behaviour of the model following the addition of creatine kinase, the model was fitted to PCr/ATP ratio data in rats [4]. Because the model was most sensitive to ANT among the internal reactions (Appendix F), we decided to update the value of μ^a_{ANT} to fit with this dataset. Each data point contained a value of the PCr to ATP ratio, given by $(\mathrm{PCr/ATP})_i$, for a given workload w_i. Workloads were matched to the data by using 54921 mmol O_2.kg dw^{-1}.min^{-1}.(s.L mito.mol^{-1}) as the conversion factor between v_{C4} (in mol/L mito/s) to VO_2 (in mmol O_2/kg dw/min) [19], with linear interpolation used to estimate PCr/ATP ratios between simulated workloads.

The model prediction $(c_{\mathrm{PCr}}/c_{\mathrm{ATP}})_i$ was evaluated against the data using a sum of squares cost function with a regulariser to maintain consistency with the original parameter value ($\mu^a_{\mathrm{ANT0}} =$

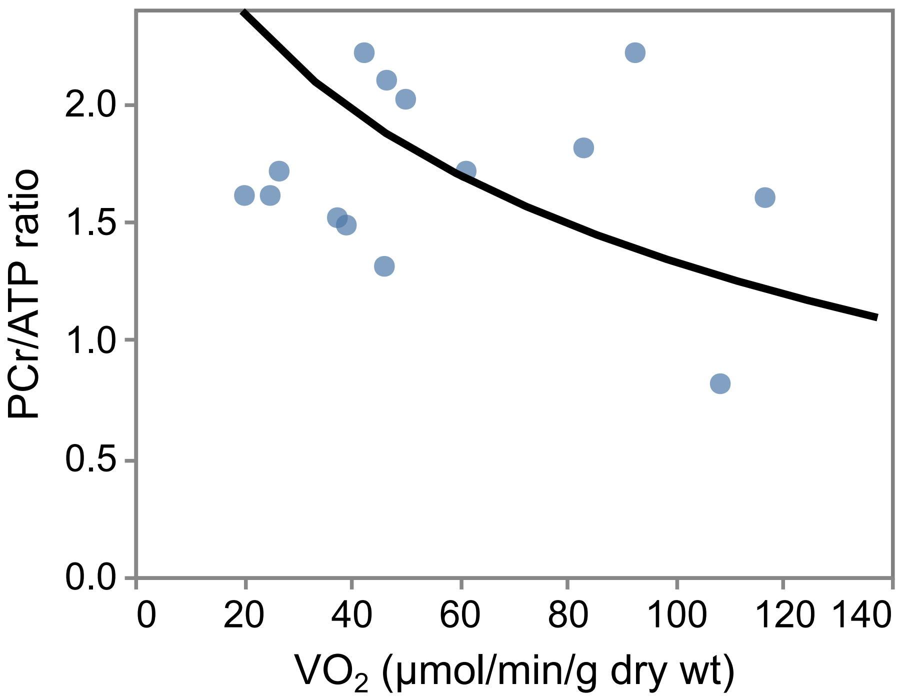

Figure Q: Fit of model against PCr/ATP ratio data. Model predictions are shown in the black line, and data points are shown in blue dots. The model was fitted using a sum of squares objective function with a regularisation term.

-11.96) generated from the Beard model:

$$J(\mu^a_{\mathrm{ANT}}) = \sum_i \left[(\mathrm{PCr}/\mathrm{ATP})_i - (c_{\mathrm{PCr}}/c_{\mathrm{ATP}})_i\right]^2 + \gamma(\mu^a_{\mathrm{ANT}} - \mu^a_{\mathrm{ANT0}})^2 \tag{142}$$

where $\gamma = 0.01$. The value $\mu^a_{\mathrm{ANT}} = -8.12$ kJ/mol was found to minimise this cost function. A comparison of the model prediction to data is shown in Figure Q.

D.3 ATP synthase

The Beard model neglected the effects of proton concentration, which can in some cases lead to thermodynamic inconsistencies. The bond graph model added a dependence on pH, assuming a nominal pH of 7.2 in the Beard model. The reaction parameter μ^a_{F1} was calculated in a similar manner as that described at the start of Appendix D.

E Initial conditions

To more accurately represent the physiological state of the cardiomyocyte, the initial conditions were updated. The initial conditions for the species are given in Table 3. The membrane potential $\Delta\Psi$ was initialised to 160 mV [3].

Table 3: Initial conditions of species in the oxidative phosphorylation model. All concentrations are given in mol/L.

Species	Initial concentration	Source
$NADH_x$	0.0015	[a]
NAD_x	0.00147	[a]
Q	0.00055	[a]
QH_2	0.0008	[a]
C_{ox}	0.0017	[a]
C_{red}	0.001	[a]
O_2	2.60×10^{-5}	[a]
$mATP_i$	0.00876874	[b]
$mATP_x$	0.00932348	[b]
$fATP_i$	0.00023126	[b]
$fATP_x$	0.00024589	[b]
$fATP_c$	0.00023126	[b]
$fADP_x$	0.00011887	[b]
$fADP_i$	4.47×10^{-6}	[b]
$fADP_c$	4.47×10^{-6}	[b]
$mATP_c$	0.00876874	[b]
$mADP_i$	1.17×10^{-5}	[b]
$mADP_c$	1.17×10^{-5}	[b]
$mADP_x$	0.00031175	[b]
Pi_x	0.003	[e]
Pi_i	0.001	[b]
Pi_c	0.0048	[b]
H_x	6.31×10^{-8}	[a]
H_i	7.94×10^{-8}	[a]
H_c	7.94×10^{-8}	[a]
B_x	0.02094946	[c]
HB_x	0.01976064	[c]
Mg_c	0.00091	[d]
Mg_x	0.00091	[d]
Cr_i	0.0068	[b]
Cr_c	0.0068	[b]
PCr_i	0.0162	[b]
PCr_c	0.0162	[b]
AMP_c	3.47×10^{-5}	[b]
AMP_i	3.47×10^{-5}	[b]
K_x	0.14	[a]

[a] Beard [3]
[b] See § E.1
[c] Calculated from B_t (§ D.1). Assuming the buffering reaction is at equilibrium, $c_{B_x} = B_t/(1 + c_{H_x}/K)$ and $c_{HB_x} = B_t - c_{H_x}$, where B_t is the total amount of buffer and K is the dissociation constant.
[d] Watanabe and Konishi [20]
[e] Set to 3 mM to favour the forward reaction of the ATP synthase reaction

E.1 Energetic molecules

The initial values of several metabolites were updated to reflect more physiologically realistic ranges of concentrations. The derivation of these is outlined below.

The total concentration of ATP, ADP, Pi, Cr and and PCr were taken from the first row of Table 2 of Aliev and Saks [21]. The concentrations for Pi_c, Cr_c, Cr_i, PCr_c and PCr_i, were taken directly from these values. The values for ATP and ADP were taken to be the total concentrations in the cytosol and intermembrane space, with the dissociation constant used to calculate the free and Mg-bound forms, namely

$$c_{\mathrm{fATP}} = \frac{c_{\mathrm{ATP,tot}}}{1 + c_{\mathrm{Mg}}/K_{DT}} \tag{143}$$

$$c_{\mathrm{mATP}} = c_{\mathrm{ATP,tot}} - c_{\mathrm{fATP}} \tag{144}$$

$$c_{\mathrm{fADP}} = \frac{c_{\mathrm{ADP,tot}}}{1 + c_{\mathrm{Mg}}/K_{DD}} \tag{145}$$

$$c_{\mathrm{mADP}} = c_{\mathrm{ADP,tot}} - c_{\mathrm{fADP}}. \tag{146}$$

AMP concentrations were initialised assuming equilibrium of the adenylate kinase reaction:

$$c_{\mathrm{AMP}} = \frac{c_{\mathrm{fADP}}^2}{c_{\mathrm{fATP}}} E(2\mu_{\mathrm{fADP}}^0 - \mu_{\mathrm{fATP}}^0 - \mu_{\mathrm{AMP}}^0). \tag{147}$$

Equation 2 of Aliev and Saks was used to calculate initial ADP and ATP concentrations in the matrix:

$$c_{\mathrm{ADP_x}} = \frac{c_{\mathrm{ADP_i}} c_{\mathrm{ANP_x}}}{c_{\mathrm{ADP_i}} + c_{\mathrm{ATP_i}}/\beta} \tag{148}$$

$$c_{\mathrm{ATP_x}} = c_{\mathrm{ANP_x}} - c_{\mathrm{ADP_x}} \tag{149}$$

$$c_{\mathrm{ANP_x}} = 0.01\ \mathrm{M} \tag{150}$$

$$\beta = 25. \tag{151}$$

Equations analogous to Eq. 143–146 were used to calculate the concentrations of free and Mg-bound forms.

F Sensitivity analysis

To assess the sensitivity of the model to parameters, the model was run with perturbations to the parameters. The parameters associated with the reaction rates ($E(\mu^a)$) were increased or decreased by 30%, and the changes to ATP consumption rate relative to the baseline at steady state were recorded (Table 4). The model is most sensitive to changes in rates for the ATPase, driving force for the dehydrogenase reaction and rate of the dehydrogenase. The model shows only modest sensitivity to ANT, which is the most sensitive out of the internal reactions. The sensitivity to the mitochondrial complexes is low, which appears to be consistent with experimental measurements of mitochondrial respiration showing low sensitivities to complex I and IV activities [22].

Table 4: Effect of perturbing parameters on workload. Parameters were perturbed by ±30%, and the relative change to J_{ATPase} at steady state was recorded.

Parameter	−30%	+30%
μ^a_{ATPase}	-0.29896	0.293336
μ_r	-0.06149	0.000431
μ^a_{DH}	-0.05641	0.000348
μ^a_{ANT}	-0.00101	0.000513
μ^a_{MgADP}	-0.00045	0.000264
μ^a_{fADPt}	-0.00011	8.27×10^{-5}
μ^a_{Hle}	0.000103	-0.00012
μ^a_{MgATP}	-0.0001	5.02×10^{-5}
μ^a_{C3}	-4.75×10^{-5}	2.76×10^{-5}
μ^a_{C1}	-3.69×10^{-5}	2.46×10^{-5}
μ^a_{C4}	-3.64×10^{-5}	2.32×10^{-5}
μ^a_{PiHt}	-2.17×10^{-5}	9.72×10^{-6}
μ^a_{fATPt}	1.72×10^{-5}	-1.25×10^{-5}
μ^a_{CKi}	-1.13×10^{-6}	6.12×10^{-7}
μ^a_{AMPt}	-1.11×10^{-6}	7.55×10^{-7}
μ^a_{AK}	-1.08×10^{-6}	8.62×10^{-7}
μ^a_{mATPt}	-2.14×10^{-7}	1.17×10^{-7}
μ^a_{mADPt}	8.20×10^{-8}	-8.09×10^{-8}
μ^a_{F1}	-7.45×10^{-8}	4.02×10^{-8}
μ^a_{CK}	-3.98×10^{-8}	2.12×10^{-8}
μ^a_{Pit}	-2.13×10^{-8}	1.19×10^{-8}
μ^a_{KH}	7.80×10^{-10}	-1.30×10^{-10}

G Simulation protocol

The omics analysis of metabolites showed that abundances of both NAD and NADH were increased by 43% and 65% respectively in older cardiomyocytes (Fig. 5, main text). It was also observed that the abundances of creatine and phosphocreatine were decreased by 29% and 8% respectively in older cardiomyocytes, resulting in a 14% reduction in total creatine, assuming that fold-changes are applied to the initial conditions. To infer the functional consequences of these fold changes, we used the bond graph model to simulate the effects of these changes. The trends seen in older hearts were simulated by using the baseline model (Appendix C) to represent the younger group and applying the observed fold changes in NAD, NADH, creatine and phosphocreatine concentrations to represent the old group. NAD and NADH concentrations were held constant in these simulations.

Each of the models was simulated for a range of values for μ^a_{ATPase} to reflect the effects of varying workloads. The rate of oxygen consumption was defined as $\text{VO}_2 = 0.5 J_{\text{C4}}$. Results from the model were recorded at steady state, after simulating for 1000 seconds. Fig. 6f of the main text shows the results under a high workload (1.21 mol O_2/L mito/s) while Fig. 6g–h show the PCr/ATP ratios and ADP concentrations for different workloads.

References

1. Gawthrop, P. J. & Crampin, E. J. Energy-based analysis of biochemical cycles using bond graphs. *Proceedings of the Royal Society of London A: Mathematical, Physical and Engineering Sciences* **470**, 20140459 (2014).

2. Gawthrop, P. J. & Pan, M. Network thermodynamics of biological systems: A bond graph approach. *Mathematical Biosciences* **352**, 108899 (2022).

3. Beard, D. A. A biophysical model of the mitochondrial respiratory system and oxidative phosphorylation. *PLOS Computational Biology* **1**, e36 (2005).

4. Vendelin, M., Kongas, O. & Saks, V. Regulation of mitochondrial respiration in heart cells analyzed by reaction-diffusion model of energy transfer. *American Journal of Physiology-Cell Physiology* (2000).

5. Dawson, T. H. Allometric relations and scaling laws for the cardiovascular system of mammals. *Systems* **2**, 168–185 (2014).

6. Lemieux, H., Semsroth, S., Antretter, H., Höfer, D. & Gnaiger, E. Mitochondrial respiratory control and early defects of oxidative phosphorylation in the failing human heart. *International Journal of Biochemistry & Cell Biology* **43**, 1729–1738 (2011).

7. Gnaiger, E., Lassnig, B., Kuznetsov, A., Rieger, G. & Margreiter, R. Mitochondrial oxygen affinity, respiratory flux control and excess capacity of cytochrome c oxidase. *Journal of Experimental Biology* **201**, 1129–1139 (1998).

8. Barth, E., Stämmler, G., Speiser, B. & Schaper, J. Ultrastructural quantitation of mitochondria and myofilaments in cardiac muscle from 10 different animal species including man. *Journal of Molecular and Cellular Cardiology* **24**, 669–681 (1992).

9. Bazil, J. N., Beard, D. A. & Vinnakota, K. C. Catalytic coupling of oxidative phosphorylation, ATP demand, and reactive oxygen species generation. *Biophysical Journal* **110**, 962–971 (2016).

10. Wu, F., Yang, F., Vinnakota, K. C. & Beard, D. A. Computer modeling of mitochondrial tricarboxylic acid cycle, oxidative phosphorylation, metabolite transport, and electrophysiology. *Journal of Biological Chemistry* **282**, 24525–24537 (2007).

11. Flamholz, A., Noor, E., Bar-Even, A. & Milo, R. eQuilibrator–the biochemical thermodynamics calculator. *Nucleic Acids Research* **40**, D770–D775 (2012).

12. Ould-Moulaye, C. B., Dussap, C. G. & Gros, J. B. A consistent set of formation properties of nucleic acid compounds: Purines and pyrimidines in the solid state and in aqueous solution. *Thermochimica Acta* **375**, 93–107 (2001).

13. Beard, D. A. & Qian, H. *Chemical Biophysics: Quantitative Analysis of Cellular Systems* (Cambridge University Press, Cambridge, England, UK, 2008).

14. Vinnakota, K. C. & Bassingthwaighte, J. B. Myocardial density and composition: a basis for calculating intracellular metabolite concentrations. *American Journal of Physiology-Heart and Circulatory Physiology* (2004).

15. Gawthrop, P. J., Cudmore, P. & Crampin, E. J. Physically-plausible modelling of biomolecular systems: A simplified, energy-based model of the mitochondrial electron transport chain. *J. Theor. Biol.* **493**, 110223 (2020).

16. Tran, K., Loiselle, D. S. & Crampin, E. J. Regulation of cardiac cellular bioenergetics: mechanisms and consequences. *Physiol. Rep.* **3**, e12464 (2015).

17. Pan, M., Gawthrop, P. J., Cursons, J. & Crampin, E. J. Modular assembly of dynamic models in systems biology. *PLOS Computational Biology* **17**, e1009513 (2021).

18. Keener, J. & Sneyd, J. *Mathematical Physiology* (Springer, New York, 2009).

19. Ghosh, S. *et al.* Insights on the impact of mitochondrial organisation on bioenergetics in high-resolution computational models of cardiac cell architecture. *PLOS Computational Biology* **14**, e1006640 (2018).

20. Watanabe, M. & Konishi, M. Intracellular calibration of the fluorescent Mg2+ indicator furaptra in rat ventricular myocytes. *Pflügers Archiv* **442**, 35–40 (2001).

21. Aliev, M. K. & Saks, V. A. Compartmentalized energy transfer in cardiomyocytes: use of mathematical modeling for analysis of in vivo regulation of respiration. *Biophysical Journal* **73**, 428–445 (1997).

22. Lucas, D. T. & Szweda, L. I. Declines in mitochondrial respiration during cardiac reperfusion: Age-dependent inactivation of α-ketoglutarate dehydrogenase. *Proceedings of the National Academy of Sciences* **96**, 6689–6693 (1999).
