# Supplementary material for: The Human Cardiac “Age‐OME”: Age‐Specific Changes in Myocardial Molecular Expression
Source: Aging Cell. 2025 Sep 7;24(11):e70219. doi: 10.1111/acel.70219 (PMC12610413; doi:10.1111/acel.70219)
Supplement: Supplementary file 1 — Data S1. acel70219‐sup‐0001‐DataS1.zip. [file ACEL-24-e70219-s011.zip › Supplementary Information.docx]

**Supplementary Information**

**Supplementary Methods**

**Sample size calculation**

The sample size calculation was performed assuming matched biological sex*.* To determine the sample size calculation, we defined the effect size for the i-th analyte as

$\Delta_{i}=\frac{\delta_{i}}{\sigma_{i}}$,

where $n$ is the sample size, $\delta_{i}=\log_{2} FC_{i}$, set to $\delta_{i}=3\sigma_{i},$ and $\sigma_{i}$ is the standard deviation of the i-th analyte. Our minimum sample size is then computed focusing on the effect size, namely $\Delta_{i}=3$ for each i-th analyte in the Limma framework ^1, 2^. For the sample size calculation, we set the probability of type II error, $\beta$, equal to $0.1$, which leads to the power of the test of at least $1-\beta= 0.9$.

We set $\alpha_{\mathrm{fdr}}=0.05$, probability of type I error (after FDR correction) [A3.4], where the $\alpha_{\mathrm{fdr}}$ is the type I error corrected by a false discovery rate. This is processed by assuming that only 5% are truly altered amongst all analytes. Further details on the software to compute the sample size calculation with adjustment for false discovery rate are reported in Liu and Hwang (2007) ^1^.

The minimal sample size is $n = 7$ samples in each cohort. Since high-throughput technologies cannot guarantee the detection of each analyte's peak in each sample, to avoid the missingness of the values affected, we also decided to consider only analytes that are present at least in 75% of the samples in each cohort. This consideration leads to recompute the sample size for each cohort, which will be indicated with n^∗^, by solving the following equation

$$1:0.75=n^{*}:n$$

where $n = 7$, from Equation 5.2. Solving Equation 5.3

$$n^{*}=n/0.75$$

$= 9.333$.

Consequently, the sample size for each cohort should be at least $n^{*}\approx10$.

**Proteomics**

Frozen heart tissue was powdered in liquid nitrogen and approximately 10mg was weighed and homogenized in 4% sodium deoxycholate and 100mM Tris-HCl pH 7.5. Samples were heated to 95^o^C and then sonicated at 70% amplitude using QSonica R2 (Q Sonica). Samples were then centrifuged at 18,000 x g and the supernatant was collected. Protein concentration was determined by BCA assay (Pierce). 20ug of protein for each sample underwent trypsin digestion overnight at 37^o^C before peptides were prepared for mass spectrometry as described previously ^3^. Peptide samples were directly injected onto a 30cm x 70 um C18 (Dr. Maisch, Ammerbuch, Germany, 1.9 µm particle size) fused silica analytical column with a 10 µm pulled tip, coupled online to a nanospray ESI source. Peptides were resolved over a gradient from 5% - 40% acetonitrile over 120 min with a flow rate of 300 nL/min. Peptides were ionized by electrospray ionization at 2.3 kV. MS/MS analysis was performed using a Q-Exactive Fusion Lumos mass spectrometer (ThermoFisher) with 27% normalised HCD collision energy for fragmentation. Spectra were attained in a Data-Independent Acquisition (DIA) using 20 variable isolation windows.

RAW data files including the high pH fractions were analysed using the integrated quantitative proteomics software DIA-NN ^4^ (version 1.7). The database provided to the search engine for identification contained the human database downloaded on the 5th of May 2020. FDR was set to 1% of precursor ions to remove interferences. The match between runs was enabled. Trypsin was set as the digestion enzyme with a maximum of 2 missed cleavages. Carbamidomethylation of Cys was set as a fixed modification and oxidation of Met were set as variable modifications. Retention time dependent profiling was used and quantification setting was set to any LC (high accuracy). Protein inference was based on genes. Neural network classifier was set to double-pass mode. The MaxLFQ algorithm was used for label-free quantitation, integrated into the DIA-NN ^5, 6^.

**Metabolomics**

Polar metabolites were extracted from frozen heart tissues as previously described ^7^. Briefly, approximately 50 mg of ground heart tissue was weighed into 2 mL Eppendorf tube and subjected to a three-phase extraction protocol involving tissue, ice-cold extraction medium (methanol:water, and chloroform). Samples were then centrifuged at 14 300 rpm at 4 °C for 25 min. The aqueous layer was transferred into a new microfuge tube, concentrated in the Speed-Vac SPD120 (Thermo Fisher Scientific) and dried under nitrogen stream, followed by reconstitution in the acetonitrile/methanol/formic acid (75:25:0.2; v/v/v, HPLC grade; Thermo Fisher Scientific) for the HILIC analysis, and acetonitrile/methanol (25:25; v/v/v, HPLC grade) for the AMIDE analysis. Targeted metabolite profiling used in this study was established using reference standards for each individual metabolite to determine MS multiple reaction-monitoring transitions, declustering potentials, and collision energies and chromatographic retention time, as described previously ^7, 8^.

For both HILIC and AMIDE analysis, LC-MS/MS system composed of an Agilent 1260 Infinity liquid chromatography (Santa Clara, CA, USA) system coupled to a QTRAP5500 mass spectrometer (AB Sciex, Foster City, CA, USA) was used. The polar metabolites in both positive and negative ionization mode were separated in hydrophilic interaction liquid chromatography (HILIC) mode using an Atlantis^®^ HILIC column (Waters) and a XBridge^TM^ Amide column (Waters), respectively, which allow the separation of metabolites of different properties, both as previously described ^7, 9^. Sample analysis was conducted in a randomized sample order and data was acquired in the same batch on the same day. To account for variations during the extraction procedure, internal standards were added to the extraction buffer (Phenylalanine-d8 (Phe-d8), Valine-d8 (Val-d8), Thymine-d4 (Thy-d4), and Citrate-d4 (Cit-d4)). The analysis software MultiQuant 3.0 (ABSciex) was used for Multiple Reaction Monitoring (MRM) Q1/Q3 peak integration of the raw data files (Analyst software, v.1.6.2; ABSciex). The log_2_ transformed relative abundances were then normalised using EigenMS ^10^.

**Lipidomics**

Lipids were extracted from ~20 mg heart tissue using a two-phase method with methyl-tert-butyl ether (MTBE) and water ^11^. Tissue was homogenised with steel beads in 250 µL methanol containing 0.01% (w/v) butylhydroxytoluene (BHT) and mass spectrometry internal standards: 2 nmol PC(19:0/19:0); 1 nmol each of SM(d18:1/12:0), GluCer(d18:1/12:0), Cer(d18:1/17:0), PS(17:0/17:0), PE(17:0/17:0), PA(17:0/17:0), PI(d7-18:1/15:0), PG(17:0/17:0), CL(14:0/14:0/14:0/14:0), and TG(17:0/17:0/17:0); 0.5 nmol each of DG(d7-18:1/15:0), CholE(17:0), LPC (17:0), LPE(17:1), and AcCa(d3-16:0); and 0.2 nmol each of Sph(d17:1), S1P(d17:1), LacCer(d18:1/12:0), and MG(d7-18:1). MTBE (850 µL) was added, and samples were sonicated in a 4°C water bath for 30 min. Mass spectrometry grade water (212 µL) was added to induce phase separation after vortexing and centrifugation at 2000g for 5 min. The upper organic phase was collected in 5 mL glass tubes and the aqueous phase was extracted twice more with 500 µL MTBE and 150 µL methanol followed by sonication for 15 min and phase separation with 125 µL water. Organic phases from the three extractions were combined and dried under vacuum in a Savant SC210 SpeedVac (ThermoFisher Scientific). Lipids were reconstituted in 400 µL 80% methanol/20% water/0.1% formic acid containing 0.01% (w/v) BHT and stored at -80 °C.

A ThermoFisher Q-Exactive HF-X mass spectrometer coupled to a Vanquish HPLC with a 2.1x100 mm Waters C18 HPLC column (1.7 µm pore size) was used for LC-MS/MS, with minor modifications to our previously-reported method ^11^. HPLC solvent A was 10 mM ammonium formate, 0.1% formic acid in acetonitrile:water (60:40), and solvent B was 10 mM ammonium formate, 0.1% formic acid in isopropanol:acetonitrile (90:10). A 27 min binary gradient at 0.28 mL/min was used: 0 min, 80:20 A/B; 3 min, 80:20 A/B; 5.5 min, 55:45 A/B; 8 min, 35:65 A/B; 13 min, 15:85 A/B; 14 min, 0:100 A/B; 20 min, 0:100 A/B; 20.2 min, 70:30 A/B; 27 min, 70:30 A/B. Data was acquired in full scan/data-dependent MS^2^ mode (full scan resolution 60,000 FWHM, scan range 220–1600 *m/z*). Sample order was randomised, and data was collected in both positive and negative mode for each sample. The ten most abundant ions in each cycle were subjected to MS^2^, with an isolation window of 1.4  *m/z*, collision energy 30  eV, resolution 17,500 FWHM, maximum integration time 110  ms and dynamic exclusion window 10 s**.** An exclusion list of background ions was used based on a solvent blank. An inclusion list was used for all internal standards. LipidSearch software (version 4.2, Thermo Fisher) was used for lipid annotation, chromatogram alignment, and peak integration. Lipid annotation required both accurate precursor ion mass (tolerance 5 ppm) and diagnostic product ions (tolerance 8 ppm). Individual lipids were expressed as ratios to the class-specific internal standard, then multiplied by the amount of internal standard. Then, the log_2_ transformed abundandance was EigenMS normalised.

**RNA extraction and sequencing**

Approximately 50mg of frozen heart tissue was transferred to a 2ml tube containing one 5mm stainless steel bead (Qiagen) and was disrupted and homogenised in TRIzol reagent (Invitrogen) using the TissueLyser LT (Qiagen). RNA extraction was then performed according to TRizol manufacturer’s instructions. The extracted RNA was then DNase treated using the Qiagen RNAse-Free DNase Set and subsequently purified using the RNase Mini kit (Qiagen) according to manufacturer’s instructions. The concentration and quality of the RNA were assessed using a Nanodrop, where 260/230 and 260/280 ratios were evaluated. Additionally, the integrity of the RNA was assessed using an RNA Nano Chip on an Agilent Bioanalyzer.

RNA-Seq libraries were prepared with Illumina Stranded Total RNA prep Ligation with Ribo Zero Plus according to manufactures instructions. The RNA-seq libraries were sequenced using a paired-end 250bp kit on a S4 flow cell of the Illumina NovaSeq 6000. Library preparation and sequencing were performed by the Ramaciotti Centre for Genomics, at the University of New South Wales, Australia.

The quality of each RNA sequence was assessed using ShortRead ^12^. The raw sequences were aligned on hg38 genome assembly (UCSC) with Rsubread version 2.2.1 ^13, 14^. The alignment was performed on pair-end sequences without trimming. Rsubread was also used for generating the gene count matrix. The gene counts were converted in Count Per Million (CPM), log2 transformed, and then normalised using Voom ^15^.

**Immunofluorescence and Confocal microscopy**

LV tissue from a subset of samples used in the omics analysis (Younger n=6, Older n= 6) were prepared for immunostaining. The methodology was adapted from a previously published protocol ^16^. Following overnight incubation at -30°C, samples were embedded in optimal cutting temperature solution (OCT, SHH0026, Sigma-Aldrich) and 15-µm sections were cut. Sections were obtained in duplicate from a randomly selected initial depth and at 300 and 600 µm deeper relative to the first sample. Therefore, there were 6 sections for each unique sample used for staining. The slides were then stored at -80°C until required.

All incubations were at room temperature unless stated otherwise. The LV sections were fixed for 15 minutes with 10% neutral buffered formalin (Sigma-Aldrich). Samples were subsequently washed three times for 5 minutes in phosphate buffered saline (PBS, Sigma) and then incubated in 0.2% (vol/vol) glycine (Ajax Finechem) in PBS for 10 minutes. Samples were then washed in PBS before being permeabilised with 0.5% Triton X-100 (Promega) diluted in PBS for 20 minutes. Samples were then washed three times for 5 minutes in PBS, then incubated in blocking solution (5% normal goat serum (ThermoFisher), 5% acetylated bovine serum albumin (Sigma-Aldrich) in PBS) for 1 hour. The samples were then washed three times for 5 minutes and incubated with mouse anti-SERCA2 ATPase (1:100, #MA3-919, Invitrogen) and rabbit anti-MYOM2 (1:50, #HPA001765, Atlas Antibodies) or anti-MYH6 (1:500, #ab207926, Abcam) for 3 hours at 37°C. After three washes with PBS, samples were incubated with F(ab')2-Goat anti-mouse IgG (H+L) conjugated to Alexa Fluor 594 (1:200, #A-11020, Invitrogen) and F(ab')2-Goat anti-rabbit IgG (H+L) conjugated to Alexa Fluor 488 (1:200, #A48282, Invitrogen). Subsequently, samples were washed three times for 5 minutes in PBS and then incubated with wheat germ agglutinin conjugated to Alexa Fluor 647 (WGA-647; 1:200, #W32466, ThermoFisher) for 1 hour. Samples were then washed three times for 5 minutes in PBS and incubated in DAPI (1 µg/ml in PBS; #62248, Thermo Scientific) for 10 minutes. Finally, coverslips (#10474379, Carl Zeiss) were mounted onto the samples with ProLong Diamond Antifade Mountant (#P36961, Invitrogen) and left to cure at room temperature for 24 hours, shielded from light. Samples were then stored at -30°C until imaging.

LV tissue was imaged using a Leica Thunder 3D Imager (Leica Microsystems). Images were first acquired using a 10× objective (HC PL FLUOTAR, 0.32 NA) via tile scanning with a 10% overlap. Adjacent tiles were then stitched together using the built-in Leica LAS X software. Bulb power and exposure time were maintained across a given experiment for each channel. Excitation and emission bands were configured for their respective fluorophores as follows: DAPI (405ex/450–490em), Alexa Fluor 488 (479ex/507–531em), Alexa Fluor 555 (554ex/578–610em), Alexa Fluor 594 (578ex/602–680em), Alexa Fluor 647 (638ex/666–724em), and Alexa Fluor 770 (730ex/770–850em).

LV tissue was imaged using a Leica TCS SP8 STED 3X microscope (Leica Microsystems, Australia) in confocal mode. Images were acquired using a 93× glycerol objective (HC PL APO, 1.30 NA, #11506417). The fluorophores were excited using either an ultraviolet (UV) laser for DAPI or a tunable white light laser (WLL) for other fluorophores. Laser power, laser lines, and spectral filters for photomultiplier tubes (PMT) and hybrid detectors (HyD) varied between experiments and were configured as follows: for SERCA2/MYOM2, DAPI was excited with a UV laser at 0.5% power (405ex/415–574em) and detected using a PMT; Alexa Fluor 488 was excited with the WLL at 60% power (500ex/510–600em) and detected using a HyD; Alexa Fluor 594 F(ab’)2 was excited with the WLL at 20% power (590ex/600–640em) and detected using a HyD; and WGA-647 was excited with the WLL at 6% power (650ex/660–779em). For MYH6, DAPI was excited with a UV laser at 2% power (405ex/415–530em) and detected using a PMT; Alexa Fluor 594 F(ab’)2 was excited with the WLL at 60% power (590ex/600–640em) and detected using a HyD; and WGA-647 was excited with the WLL at 40% power (650ex/660–794em).

Images were captured at Nyquist sampling rates and for slide-mounted samples utilising the Alexa Fluor 594, the step size was set to 0.18µm. An acquisition speed of 400 Hz accompanied by averaging of 3 line scans was used to acquire images throughout all experiments. To select images that represent each group (younger and older), the image with the median fluorescence intensity for that group was chosen as the representative image.

To quantify the fluorescence intensity of each section, 5 regions of interest (ROIs) were selected away from artefact and sample edges. The fluorescence intensity was then measured within cardiomyocytes found in the ROI. Five ROIs were measured within one section. As there were 6 sections per unique sample, 30 ROIs were therefore created per sample and were averaged. The fluorescence intensity was measured using Image J. To confirm the findings of proteomics analyses, the ROI mean intensities of SERCA and MYH6 were log_2_ transformed and applied a one-sided t-test to test if SERCA and MYH6 decrease with ageing.

**In-Silico Dyadic Calcium Sparks**

The potential effects of changing SERCA2 expression due to age on calcium spark dynamics were investigated using a previously published mathematical model of the cardiac dyad ^17^. All parameters of the model were taken from the published paper unless stated otherwise.

The gating of each RyR was modelled as a two-state Markov process, one representing its open state, while the other is its closed state. The calcium-dependent transition rates between the two states are formalised based on the empirical association between the mean open and closed rates of sheep RyRs, obtained from lipid bilayer experiments, and cytosolic calcium concentration as previously reported ^18^. The RyR open and close transition rates are defined as $k_{open}=\min\left( 4.57\times{10}^{2}\times\left[ Ca_{cyto}^{2+} \right]^{2.12},0.8 \right)\left( ms^{-1} \right)$, and $k_{close}=0.245\times\left[ Ca_{cyto}^{2+} \right]^{-0.27}\left( ms^{-1} \right)$, respectively. The $\left[ Ca_{\mathrm{cyto}}^{2+} \right]$ indicates the calcium concentration in the dyad that is continuous with the cytosol ^18^. This study did not include inositol 1,4,5-trisphosphate receptors originally encoded in the model ^17^. The parameter$S\in\left[ 2^{-0.53233},1 \right]$ was included to incorporate the observed changes in the abundance of SERCA2 in its flux. Consequently, the SERCA2 flux equates as $J_{SERCA2}=2v_{cycle}A_{p}S$, where $v_{cycle}$ and $A_{p}$ are taken from the published model ^17^.

**SERCA2 Regulation and Contractility**

We used a previously published ^19^ computational model of cardiac electrical excitation and intracellular calcium cycling to study the effect of changes in SERCA2 expression with age. The model includes details of the fast Na^+^ current as well as different types of K^+^ currents, such as slow- and rapidly activating delayed rectifier currents to capture the electrical activity of the plasma membrane. Major ion channels and pumps involved in intracellular calcium cycling are represented in the model. These include L-Type Channels (Ca2+ L-chnl) on the transverse-tubular membrane, ryanodine receptors (RyRs) on the sarcoplasmic reticulum, the Na^+^/Ca^2+^, and Na^+^/K^+^ exchange pumps, and the sarcolemmal Ca^2+^ pump. Ca^2+^ reuptake from cytoplasm into the sarcoplasmic reticulum during the relaxation phase of the cardiac cycle is accounted for using a model of the SERCA2 pump. The predicted calcium transients from this model were then coupled to a model ^20^ of cross-bridge cycling to simulate the contractile force generated by acto-myosin interactions in the myofilaments. The myofilament model represents a functional contractile unit of troponin, tropomyosin and actin. The model assumes three cross-bridges can bind to actin binding sites in the functional contractile unit. Changes between cross-bridge states within the functional contractile unit are governed by calcium binding to troponin, and sarcomere length.

**Energy Based Model of Oxidative Phosphorylation**

The effect of simultaneous deregulation of different analytes on the oxidative phosphorylation were assessed using a newly develop model based on the bond graph framework ^21-23^. Extensive details about the model can be found in Item S1.

**Differential Expression Analysis**

Each analyte's abundance difference was tested using the two-sided LIMMA t-test on the normalised and log_2_ transformed data (Table S2) ^1, 24^. More formally, the LIMMA t-test was employed to test $H_{0}:\beta_{a}^{young}=\beta_{a}^{old}$ against $H_{1}:\beta_{a}^{young}\neq\beta_{a}^{old}$, where $\beta_{a}$ denotes the average for the log_2_ abundance of the $a^{th}$ analyte, respectively in the younger and older cohorts. The LIMMA t-test uses a linear model with link function $E\left[ \underline{y}_{a} \right]=X\underline{\beta}_{a}$, where $\underline{y}_{a}\in\mathbb{R}^{N}$ is the vector of the observed log_2_ abundances of the $a^{th}$ analyte, while $X\in\mathbb{R}^{N\times2}$ denotes the design matrix with $N=N_{old}+N_{young}$ observations. The first column of the design matrix contains ones if the log_2_ abundances are from older donors; and zero elsewhere; vice versa for the second column. The $\underline{\beta_{a}}=\left[ \beta_{a}^{old},\beta_{a}^{young} \right]^{\top}$are the related regression coefficients with variance $Var\left[ \underline{\beta} \right]=V_{a}\sigma_{a}$, where $V_{a}$ is the unscaled covariance matrix, and $\sigma_{a}^{2}$ is the residual variance. By multiplying the contrast matrix $C^{\top}=\left[ 1,-1 \right]$ with $\underline{\beta}$, it leads to log_2_ Fold Change (log_2_FC) defined as $\alpha_{a}=\beta_{a}^{old}-\beta_{a}^{young}$, with variance $Var\left[ \alpha_{a} \right]=v_{a}\sigma_{a}^{2}$, and ${v_{a}=C}^{\top}V_{a}C$. Next, a Bayesian hierarchical model is built for $\underline{\alpha}_{a}$, with $\sigma_{a}^{-2} \sim\chi_{d_{prior}}^{2}/(d_{prior} \times s_{prior}^{2})\}$ and $\alpha_{a}|\sigma_{a}^{2} \sim N(0, v_{prior}\times\sigma_{a}^{2})$, where $v_{prior}, s_{prior}^{2}$ indicate the prior estimators for unscaled variance and the residual variance. The resulting posterior mean of $\sigma_{a}^{2}|s_{a}^{2}$ reads $\tilde{s}_{a}^{2}=\left( d_{prior}\times s_{prior}^{2}+d_{a}\times s_{a}^{2} \right)/\left( d_{prior}+d_{a} \right)$. Then, the LIMMA t-statistics is formulated as $t_{a}=\alpha_{a}/\left( \tilde{s}_{a}\sqrt{v_{a}} \right)$, distributed under the null hypothesis as t-distribution, $T_{{(d}_{prior}+d_{a})}$. The False Discovery Rate (FDR) procedure, also known as the Benjamin-Hochberg procedure, was then applied to mitigate the type I error for multiple comparisons. To calculate the posterior odds , denoted as $Od_{a}$, marginal distributions $t_{a}|(\alpha_{a}=0) \sim T_{\left( d_{prior}+d_{a} \right)}$ and $t_{a}|\left( \alpha_{a} \neq0 \right) \sim\left( 1+v_{prior}/v_{a} \right)^{1/2} \times T_{\left( d_{prior}+d_{a} \right)}$ are derived. The marginal distributions altogether with probabilities $P\left[ \alpha_{a}=0 \right] = 0.01$, and $P\left[ \alpha_{a}\neq0 \right] = 1 - P\left[ \alpha_{a}=0 \right]$ are used for calculating $P\left[ \alpha_{a}=0|t_{a},s_{a}^{2} \right]$ and $P\left[ \alpha_{a} \neq0|t_{a},s_{a}^{2} \right]$. Then, odds read $Od_{a}=P\left[ \alpha_{a}\neq0|t_{a},s_{a}^{2} \right]/P\left[ \alpha_{a}=0|t_{a},s_{a}^{2} \right]$. The analytes that exhibit FDR adjusted p-value (*P_fdr_*) less than 0.05 are then defined as differentially expressed.

For statistical tests involving ratios of analytes, such as the redox ratio of metabolites, the t-test was employed. Each ratio was tested by considering the differences in log_2_ transformed analytes’ abundances between the two groups.

**Heatmaps and Principal Component Analysis**

The heatmap was constructed on the dataset with k-nearest neighbours (kNN) imputed values, and k = 10 ^25, 26^. Then, each analyte with (*P_fdr_* < 0.1) was shifted to zero mean and used to construct the heatmap, where observations were arranged based on two independent agglomerative hierarchical clustering procedures, summarised by dendrograms. Each agglomerative clustering was performed on the Euclidean distance matrix. The Ward's minimum variance linkage was then applied to identify the clusters and their distances at each step of the clustering procedure ^27^.

The principal Component Analysis (PCA) for each class of analyte was performed on the covariance matrix, computed on the kNN, with k = 10, and imputed data ^28^. Briefly, let $D^{*}\in\mathbb{R}^{N {\times N}_{a}}$ represent the column-zero-mean-shifted dataset, with $N_{a}$ analytes and $N$ observations. Using the Singular Value Decomposition, $D^{*}=ULA^{\top}$, the rank two approximation is extracted, namely $D\approx U_{2}L_{2}A_{2}^{\top}$, where $L_{2}$ contains the two singular values with the highest magnitude, $U_{2}$ and $A_{2}$ are the left and right singular component associated to $U_{2}$ . Then the first two principal components are obtained as $P=D^{*}A_{2}\in\mathbb{R}^{N\times2}$. Then, the total variation is computed as a ratio of the sum of squared first two singular values over the total sum of squared singular values. The younger and older donors' observations contained in $P$ are utilised to estimate the parameters of bivariate normal distributions to calculate the respective 95^th^ percentiles.

**Set Enrichment Analysis**

The omics Set Enrichment Analysis (omics SEA) was performed using the Fast Gene Enrichment Analysis (FGSEA) algorithm, a method based on a multilevel-split Monte Carlo scheme to approximate the p-value of the GSEA test ^29, 30^. The SEA aims to investigate if biological functions show any evidence of dysregulation by using all the analytes. Given a particular set of analytes ($S$) of size $N_{S}$, and analytes ($A$) of size $N_{A}$ ranked in descending order according to a metric $m_{a}$, the Enrichment Score statistics (ES) by computing first $ES_{i}=\sum_{a\in A_{i}} [\delta_{a\in S}\times|m_{a}|/\left( \sum_{a\in S} m_{a} \right)-\delta_{a\notin S}/\left( N_{A}-N_{S} \right)]$, where, $\delta_{a\in S}$ is the Kronecker delta, $A_{i}$ is the set that contains the first $i$ analytes, $a_{i}$, $i=1,\ldots,N_{A},$in $A$. Next, $ES^{+}=\max_{1\leq i\leq N_{a}} ES_{i}$ and $ES^{-}=\min_{1\leq i\leq N_{a}} ES_{i}$ are computed, and the direction of the dysregulation is positive if $\left| ES^{+} \right|>\left| ES^{-} \right|$, and negative if $\left| ES^{+} \right|<\left| ES^{-} \right|$. Employing the multilevel-split Monte Carlo scheme, the hypothesis $H_{0}:ES^{+/-}=E{S_{random}}^{+/-}$ against $H_{1}:ES^{+/-}\neq E{S_{random}}^{+/-}$ is tested. Multiple testing correction FDR is then applied to the p-values. For the omics SEA, $m_{a}$ is the effect size (log_2_ fold change/pooled standard deviation) of the a^th^ analyte calculated as part of the DE analysis ^31^. The Normalised enrichment score is obtained as the ratio of the observed ES statistics and the $ES_{random}$ calculated for each set. If $\left| ES^{+} \right|>\left| ES^{-} \right|$, then the leading edges are $\{a_{1},\ldots,a_{i^{*}}\}$, where $i^{*}=\underset{1\leq i\leq N_{a}}{\mathrm{argmax}} ES_{i}$, where $a_{i}$ represents the i^th^ analyte with $A$. If the $\left| ES^{+} \right|<\left| ES^{-} \right|$, the leading edges are $\{a_{i^{*}},\ldots,a_{N_{A}}\}$.

The omics SEA was performed on the WikiPathways database (See Table S8), which contains good coverage lipid annotations in pathways as a part of a joint effort with LIPID MAPS ^32-35^. The list of WikiPathways analytes was retrieved through the graphite package ^34^. Pathways with less than 10 or greater than 50 analytes were excluded from the SEA test, as these are too small or too large to draw any specific insight. As omics SEA aims to identify potentially affected biological processes, set $A$ was constructed by prioritising protein abundances over transcript when the same gene was annotated in both omics to reflect the central dogma of molecular biology. The beta-oxidation pathway was manually curated to all the measured acylcarnitines.

The omics SEA analysis for the enrichment of the lipid classes was calculated using the lipids log_2_FC and by sorting the lipids in their respective classes according to LIPID MAPS ^36^. Then, lipid classes were inspected by plotting the normalised mmol/mg density per tissue within each cohort, namely the younger and older cohorts. Change of carbon atoms, or length, within Fatty Acids (FA), attached to the lipids were further investigated. The lipids were first divided based on the length of the fatty acids in three categories, Medium Chain (MCFA, $6 \leq FA_{length}\leq12$), Long Chain (LCFA, $13 \leq FA_{length}\leq18$) and Very Long Chain Fatty Acids (VLCFA, $FA_{length}>18$) ^37^. Each FA inherited the log_2_ fold change of the lipid divided by the pooled standard error. Next, the t-test was employed to check if the average log_2_FC in any length class of FA differs statistically from zero (See Supplementary Data 2). Additional analyses were conducted to gain insight into the relation of log_2_FC of lipids within each class, according to LIPID MAPS, and $FA_{length}$. A separate analysis was also conducted to understand if there is any linear relationship between log_2_FC of lipids within each class and the number of double carbon bonds on their fatty acid chain $\left( C=C \right)$. Both analyses have been performed using the Weighted Least Square (WLS), with weights equal to the inverse pooled standard deviation of each lipid log_2_FC, to address heteroskedasticity.

**Network Analysis**

Each omics dataset was also analysed at the network level by estimating interactions amongst analytes of each type, namely proteins, metabolites, lipids, and transcripts, using the Weighted Correlation Network Analysis (WCNA) (See Supplementary Data 6,7 and 8). This was performed on the overall data and on each cohort separately. The WCNA was constructed on the scale-free topology by using the soft thresholding by iteratively filtering pairwise correlations (edges of the networks) till the regression between the degree of analytes (node) and related probability mass reached $R^{2}=90\%$ ^38^. Successively, the random walk-based community detection algorithm was used to determine the most populated community in the network ^39^. Network calculations were performed through igraph ^40^. Using the WikiPathway database, the Over-Representation Analysis (ORA) was performed on the most populated community. The ORA aims to test if a specific pathway was represented by chance by the community. Under the null hypothesis, the statistics can be approximated as a binomial distribution. For WCNA on the lipid, the ORA was performed on the lipid classes. For the individual cohort network analyses, hubs and bottlenecks were identified using the 90^th^ quantile of the degree of centrality and betweenness.

**References**

1. Liu, P. and J.T. Hwang, Quick calculation for sample size while controlling false discovery rate with application to microarray analysis*.* *Bioinformatics*, 2007. **23**(6): p. 739-46.

2. Sullivan, G.M. and R. Feinn, Using Effect Size-or Why the P Value Is Not Enough*.* *J Grad Med Educ*, 2012. **4**(3): p. 279-82.

3. Harney, D.J., A.T. Hutchison, et al., Small-protein Enrichment Assay Enables the Rapid, Unbiased Analysis of Over 100 Low Abundance Factors from Human Plasma*.* *Mol Cell Proteomics*, 2019. **18**(9): p. 1899-1915.

4. Demichev, V., C.B. Messner, et al., DIA-NN: neural networks and interference correction enable deep proteome coverage in high throughput*.* *Nat Methods*, 2020. **17**(1): p. 41-44.

5. Cox, J. and M. Mann, MaxQuant enables high peptide identification rates, individualized p.p.b.-range mass accuracies and proteome-wide protein quantification*.* *Nat Biotechnol*, 2008. **26**(12): p. 1367-72.

6. Cox, J., N. Neuhauser, et al., Andromeda: a peptide search engine integrated into the MaxQuant environment*.* *J Proteome Res*, 2011. **10**(4): p. 1794-805.

7. Koay, Y.C., K. Stanton, et al., Effect of chronic exercise in healthy young male adults: a metabolomic analysis*.* *Cardiovasc Res*, 2020.

8. Koay, Y.C., Y.-C. Chen, et al., Plasma levels of trimethylamine-N-oxide can be increased with ‘healthy’ and ‘unhealthy’ diets and do not correlate with the extent of atherosclerosis but with plaque instability*.* *Cardiovasc Res*, 2020. **117**(2): p. 435-449.

9. Koay, Y.C., J.A. Wali, et al., Ingestion of resistant starch by mice markedly increases microbiome‐derived metabolites*.* *FASEB J*, 2019. **33**(7): p. 8033-8042.

10. Karpievitch, Y.V., S.B. Nikolic, et al., Metabolomics data normalization with EigenMS*.* *PLoS One*, 2014. **9**(12): p. e116221.

11. Turner, N., X.Y. Lim, et al., A selective inhibitor of ceramide synthase 1 reveals a novel role in fat metabolism*.* *Nat Commun*, 2018. **9**(1): p. 3165.

12. Morgan, M., S. Anders, et al., ShortRead: a bioconductor package for input, quality assessment and exploration of high-throughput sequence data*.* *Bioinformatics*, 2009. **25**(19): p. 2607-8.

13. Liao, Y., G.K. Smyth, et al., The R package Rsubread is easier, faster, cheaper and better for alignment and quantification of RNA sequencing reads*.* *Nucleic Acids Res*, 2019. **47**(8): p. e47.

14. Pan, B., R. Kusko, et al., Similarities and differences between variants called with human reference genome HG19 or HG38*.* *BMC Bioinformatics*, 2019. **20**(Suppl 2): p. 101.

15. Law, C.W., Y. Chen, et al., voom: Precision weights unlock linear model analysis tools for RNA-seq read counts*.* *Genome Biol*, 2014. **15**(2): p. R29.

16. Taper, M., G. Carrington, et al., A comparison of fixation and immunofluorescence protocols for successful reproducibility and improved signal in human left ventricle cardiac tissue*.* *J Microsc*, 2024. **296**(1): p. 34-47.

17. Chung, J., A. Tilūnaitė, et al., IP(3)R activity increases propensity of RyR-mediated sparks by elevating dyadic [Ca(2＋)]*.* *Math Biosci*, 2023. **355**: p. 108923.

18. Cannell, M.B., C.H. Kong, et al., Control of sarcoplasmic reticulum Ca2+ release by stochastic RyR gating within a 3D model of the cardiac dyad and importance of induction decay for CICR termination*.* *Biophys J*, 2013. **104**(10): p. 2149-59.

19. Iyer, V., R. Mazhari, et al., A computational model of the human left-ventricular epicardial myocyte*.* *Biophys J*, 2004. **87**(3): p. 1507-25.

20. Rice, J.J., R.L. Winslow, et al., Comparison of putative cooperative mechanisms in cardiac muscle: length dependence and dynamic responses*.* *Am J Physiol*, 1999. **276**(5): p. H1734-54.

21. Gawthrop, P.J. and E.J. Crampin, Energy-based analysis of biochemical cycles using bond graphs*.* *Proc Math Phys Eng Sci*, 2014. **470**(2171): p. 20140459.

22. Gawthrop, P.J. and M. Pan, Network thermodynamics of biological systems: A bond graph approach*.* *Math Biosci*, 2022. **352**: p. 108899.

23. Pan, M., P.J. Gawthrop, et al., Modular assembly of dynamic models in systems biology*.* *PLoS Comput Biol*, 2021. **17**(10): p. e1009513.

24. Smyth, G.K., Linear models and empirical bayes methods for assessing differential expression in microarray experiments*.* *Stat Appl Genet Mol Biol*, 2004. **3**: p. Article3.

25. Hrydziuszko, O. and M.R. Viant, Missing values in mass spectrometry based metabolomics: an undervalued step in the data processing pipeline*.* *Metabolomics*, 2012. **8**(1): p. 161-174.

26. Troyanskaya, O., M. Cantor, et al., Missing value estimation methods for DNA microarrays*.* *Bioinformatics*, 2001. **17**(6): p. 520-5.

27. Landau, S., M. Leese, et al., *Cluster analysis*. 5th ed. 2011: John Wiley & Sons.

28. Jolliffe, I.T. and J. Cadima, Principal component analysis: a review and recent developments*.* *Philos Trans A Math Phys Eng Sci*, 2016. **374**(2065): p. 20150202.

29. Sergushichev, A.A., An algorithm for fast preranked gene set enrichment analysis using cumulative statistic calculation*.* *bioRxiv*, 2016: p. 060012.

30. Subramanian, A., P. Tamayo, et al., Gene set enrichment analysis: a knowledge-based approach for interpreting genome-wide expression profiles*.* *Proc Natl Acad Sci U S A*, 2005. **102**(43): p. 15545-50.

31. Zyla, J., M. Marczyk, et al., Ranking metrics in gene set enrichment analysis: do they matter? *BMC Bioinformatics*, 2017. **18**(1): p. 256.

32. Pico, A., WikiPathways: Community Curation of Biological Pathways*.* *Nature Precedings*, 2010.

33. Pico, A.R., T. Kelder, et al., WikiPathways: pathway editing for the people*.* *PLoS Biol*, 2008. **6**(7): p. e184.

34. Slenter, D.N., M. Kutmon, et al., WikiPathways: a multifaceted pathway database bridging metabolomics to other omics research*.* *Nucleic Acids Res*, 2018. **46**(D1): p. D661-d667.

35. Soh, D., D. Dong, et al., Consistency, comprehensiveness, and compatibility of pathway databases*.* *BMC Bioinformatics*, 2010. **11**: p. 449.

36. Fahy, E., S. Subramaniam, et al., Update of the LIPID MAPS comprehensive classification system for lipids*.* *J Lipid Res*, 2009. **50 Suppl**(Suppl): p. S9-14.

37. Kihara, A., Very long-chain fatty acids: elongation, physiology and related disorders*.* *J Biochem*, 2012. **152**(5): p. 387-95.

38. Zhang, B. and S. Horvath, A general framework for weighted gene co-expression network analysis*.* *Stat Appl Genet Mol Biol*, 2005. **4**: p. Article17.

39. Pons, P. and M. Latapy. *Computing communities in large networks using random walks*. in *J. Graph Algorithms Appl*. 2006. Citeseer.

40. Csardi, G. and T. Nepusz, The igraph software package for complex network research*.* *InterJournal, complex systems*, 2006. **1695**(5): p. 1-9.

**
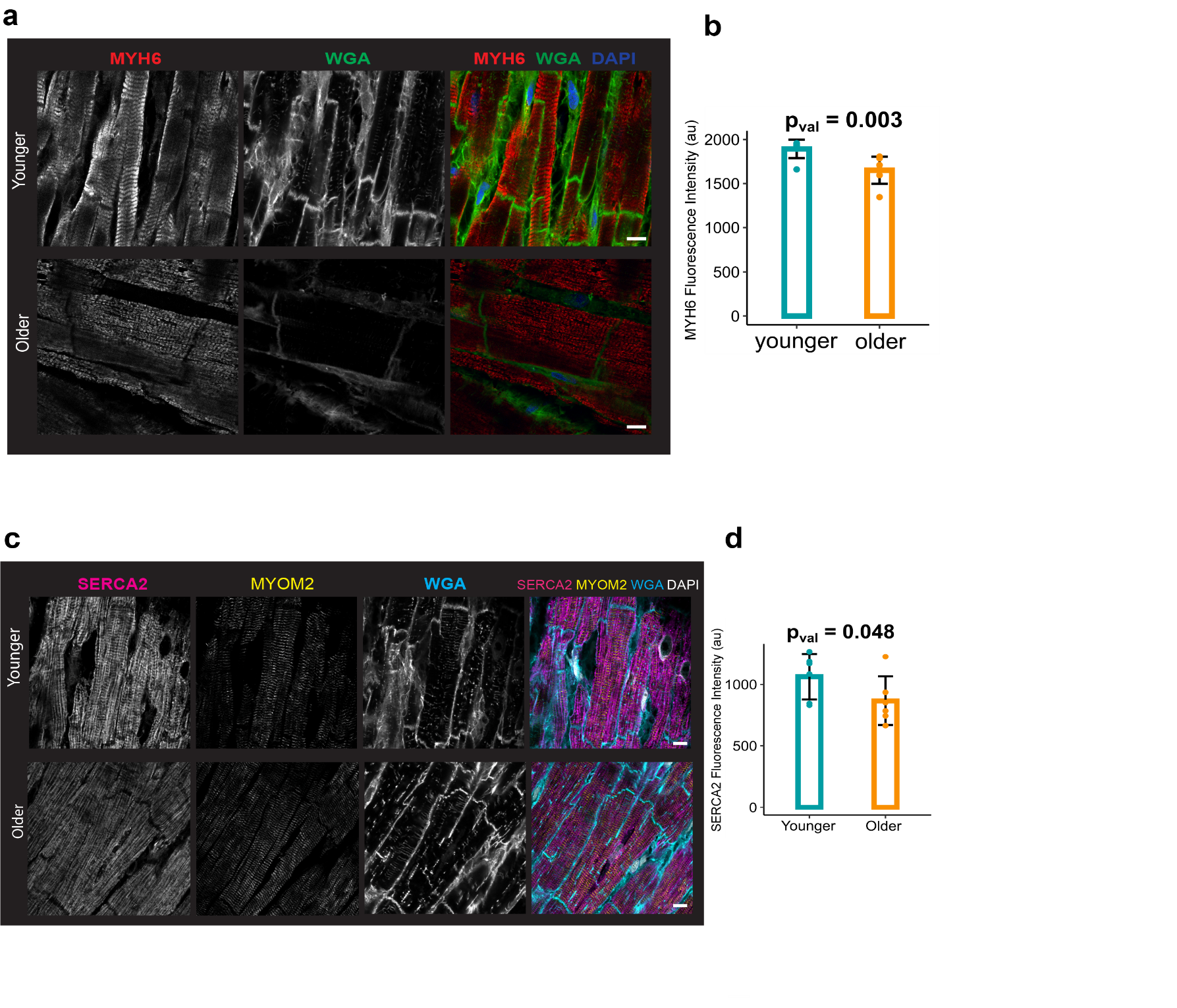
Supplementary Figures**

**Figure S1 SERCA2 and MYH6 Microscopy.** Immunofluorescence microscopy and quantification on younger and older left ventricular frozen tissue sections staining for and MYH6 (a and b) SERCA2 (c and d). (a) MYH6 (red) co-stained with WGA (green- identifying cardiomyocytes) and DAPI (blue- identifying cell nuclei). (b) Quantification of MYH6 fluorescence intensity in young (blue) vs older (orange) sections. (c) SERCA (pink) co-stained with MYOM2 (Yellow- identifying the M-line of cardiomyocytes), WGA (blue) and DAPI (white). (d) Quantification of SERCA fluorescence intensity in young (blue) vs older (orange) sections. Scale bar =10µm. au = Arbitrary units.

**
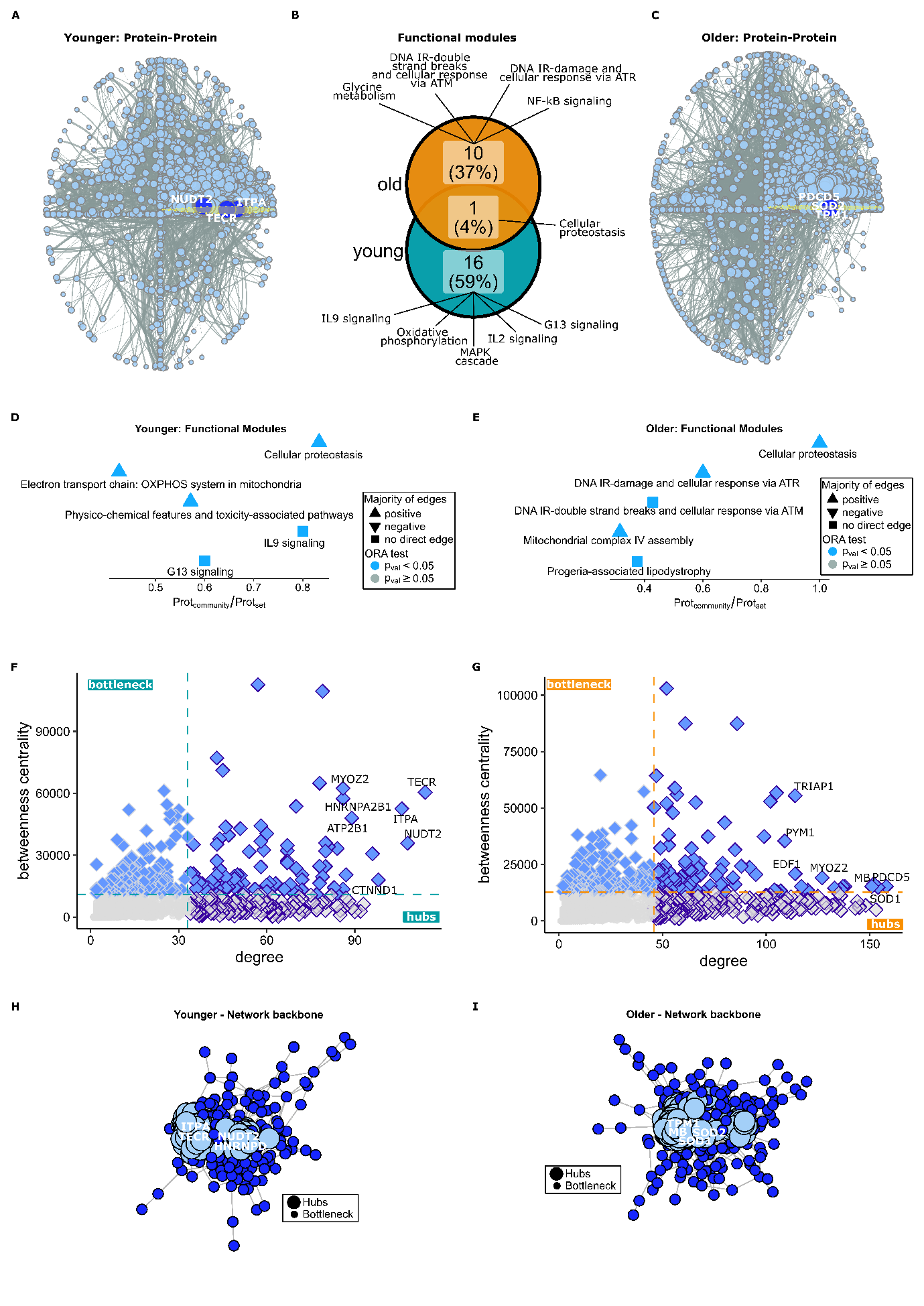
**

**Figure S2: Protein Network analysis of the younger and older cohorts.** (a) Protein network analysis of the younger cohort, with largest connected community in yellow. (b) Venn Diagram demonstrating the functional modules identified in each cohort and those that are enriched in both cohorts. (c) Protein network analysis on the older cohort with largest connected community in yellow. (d) and (e) Overrepresentation analysis (ORA) of the pathways in the largest protein community in the younger and older cohorts, respectively. An up/down-pointing triangle indicates most correlations are positive/negative, and a square is used when proteins within a pathway are not directly correlated. (f) and (g) Identification of bottlenecks and hubs making up the overall network analysis in younger and older cohorts, respectively. (h) The backbone of the younger cohort protein network. (i) The backbone of the older cohort protein network. Hubs are shown as large light blue circles. Bottleneck proteins are shown as smaller dark blue circles.


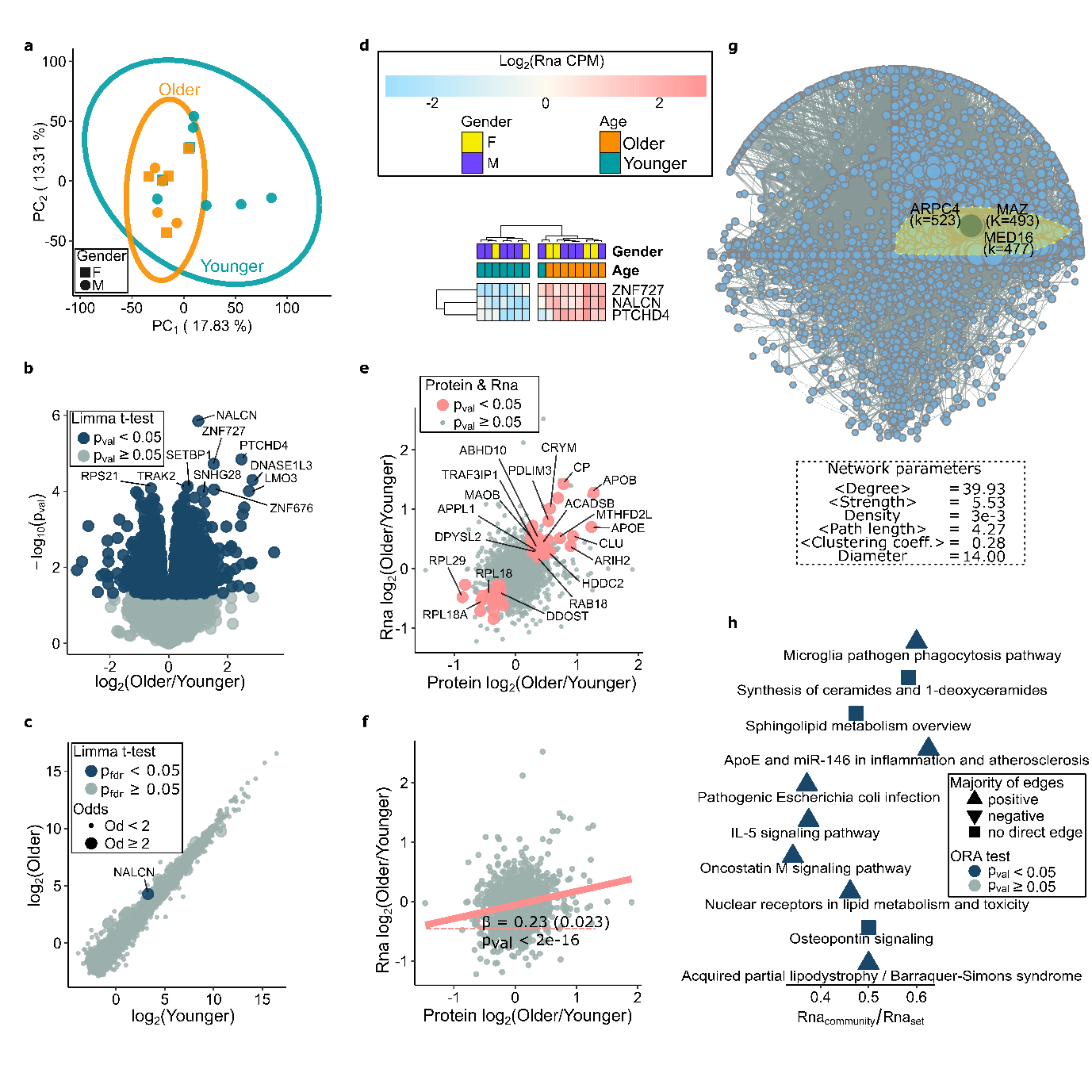


**Figure S3 Analysis of transcripts counts between younger and older hearts.** (a) Principal component analysis (PCA). The first two principal components, PC1 and PC2, are shown. PC_1_ accounts for 17.83%, and PC_2_ for 13.31%, of the total variation. The two ellipsoids represent the 95^th^ percentile of a bivariate normal distribution, respectively, for the younger and older cohorts. (b) Volcano plot of RNA counts, or expression, changes between younger and older hearts. The log_2_ fold changes and p_val_ are computed using the two-sided LIMMA t-test. The significant DE transcripts are labelled. (c) Scatter plot of log_2_ counts in the younger and older cohorts. The mean of the log_2_ RNA counts, odds and p_val_ are computed using the LIMMA t-test. The *P_fdr_* is computed using false rate discovery correction. The DE counts are labelled. (d) Heat map with hierarchical clustering of samples and DE RNA counts. The identified DE log_2_ RNA counts are employed to calculate transcript- and sample-wise dendrograms. (e) Agreement between RNA and Protein. Transcripts and proteins have been tested using LIMMA t-test and those which exhibit simultaneously $p_{val} < 0.05$ are highlighted. (f) Transcripts-Proteins trend. Using ordinary least square, log_2_ fold changes of proteins and transcripts have been analysed to detect if there is any evidence of linear relation. The log_2_ fold changes show evidence of covariance. The regression line, its slope, standard deviation and related p_val_ are reported_._ (g) Scale-free topology weighted correlation network. The nodes represent the transcripts with radius proportional to their degree, while the edges between nodes represent the correlation between pairs with width proportional to the absolute correlation. The nodes with the highest degrees are labelled. The yellow region highlights the most populated community in the network. (h) ORA of biological processes in the largest community of RNA transcripts. An up/down-pointing triangle indicates most correlations are positive/negative, and a square is used when transcripts within a pathway are not directly correlated.


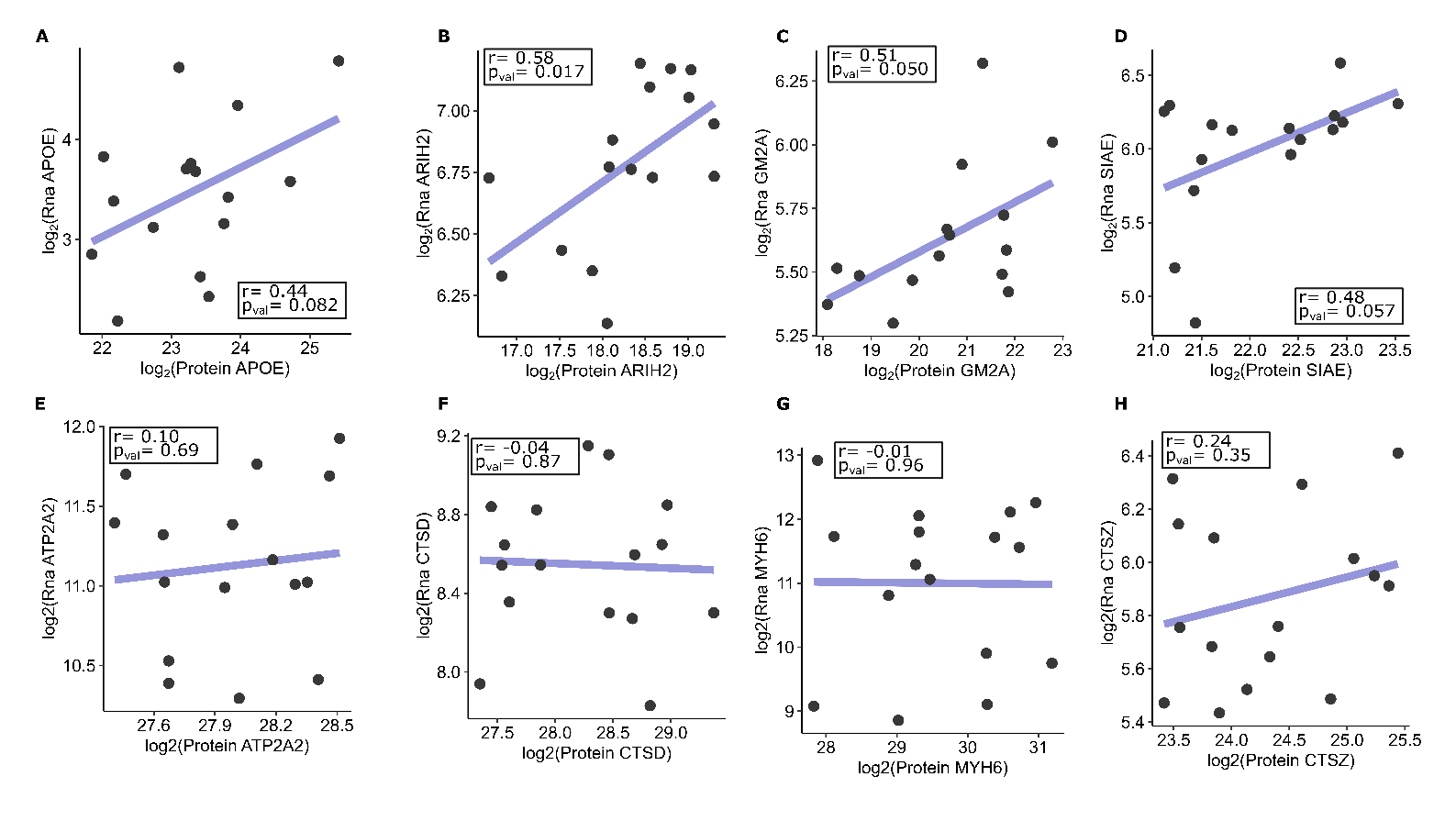


**Figure S4 Correlation between DE proteins and RNA expression**. (a)-(d) DE proteins with the strongest correlation with their corresponding RNA levels; APOE, ARIH2, GM2A and SIAE, respectively. (e)-(h) Correlation analysis results of key biologically relevant DE proteins and corresponding RNA; SERCA2 (ATP2A2), CTSD, MYH6 and CTSZ, respectively. The x-axis is the log2 protein abundance of the chosen DE protein. The y-axis is the log2 expression of the corresponding transcript.


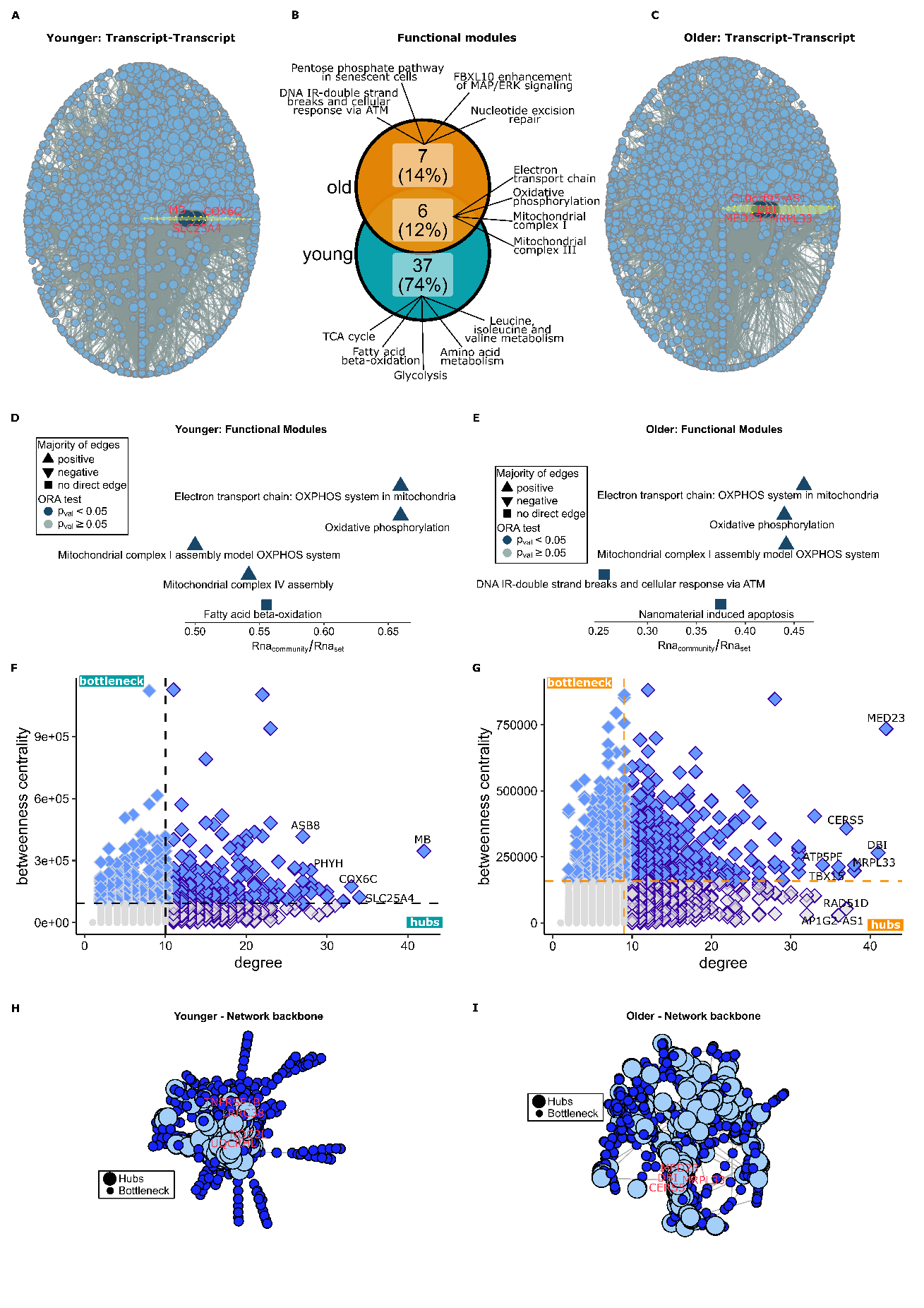
**Figure S5: RNA Network analysis of younger and older cohorts** (a) RNA transcript network analysis of the younger cohort with largest connected community in yellow. (b) Venn Diagram demonstrating the functional modules identified in each cohort and those that are enriched in both cohorts. (c) RNA network analysis on the older cohort with largest connected community in yellow. (d) and (e) Overrepresentation analysis (ORA) of the pathways in the largest RNA transcript community in the younger and older cohorts, respectively. An up/down-pointing triangle indicates most correlations are positive/negative, and a square is used when proteins within a pathway are not directly correlated. (f) and (g) Identification of bottlenecks and hubs making up the overall network analysis in younger and older cohorts, respectively. (h) The backbone of the younger cohort RNA network. (i) The backbone of the older cohort RNA network. Hubs are shown as large light blue circles. Bottleneck proteins are shown as smaller dark blue circles.

**
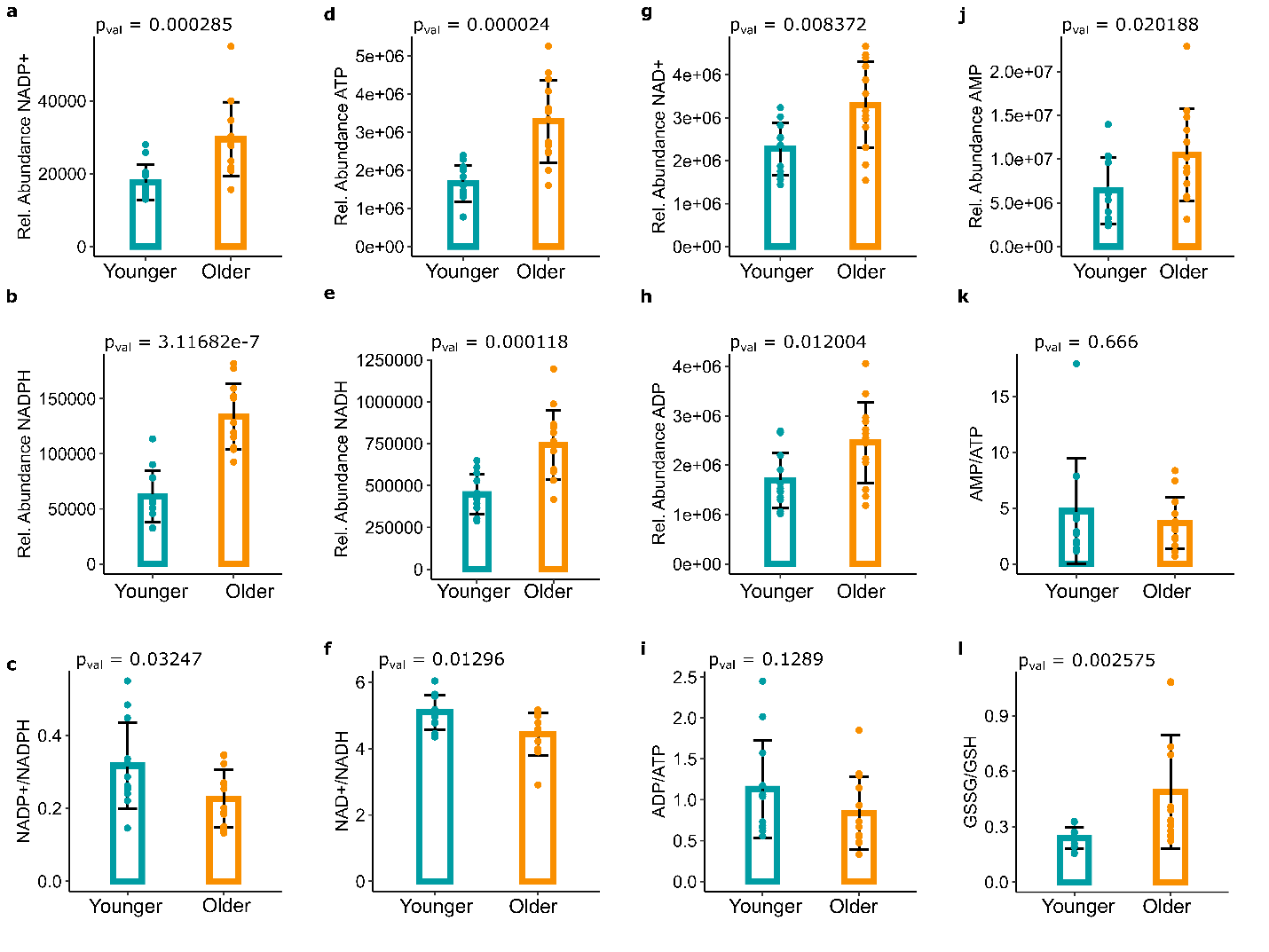
**

**Figure S6 Metabolites and Redox Ratio.** (a) The average of the relative abundances of NADP^+^ in the two cohorts are reported with related error bar, and LIMMA t-test p_val_. (b) The average of the relative abundances of NADPH in each cohort are reported with related error bar and LIMMA t-test p_val_. (c) The average of the NADP^+^/NADPH ratio in each cohort are reported with related error bar, and t-test p_val_. (d) The average of the relative abundances of ATP in the two cohort are reported with related error bar, and LIMMA t-test p_val_. (e) The average of the relative abundances of NADH in the two cohort are reported with related error bar, and LIMMA t-test p_val_. (f) The average of NADP^+^/NADPH ratio in the two cohort are reported with related error bar, and t-test p_val_. (g) The average of the relative abundances of NAD^+^ in the two cohort are reported with related error bar, and LIMMA t-test p_val_. (h) The average of the relative abundances of ADP in the two cohort are reported with related error bar, and LIMMA t-test p_val_. (i) The average ADP/ATP ratio in the two cohort are reported with related error bar, and t-test p_val_. (j) The average of the relative abundances of AMP in the two cohort are reported with related error bar, with LIMMA t-test p_val_. (k) The average of AMP/ATP ratio in the two cohort are reported with related error bar, and t-test p_val_. (l) The average of GSSG/GSH ratio in the two cohort are reported with related error bar, and t-test p_val_. All error bars represent ±SD.


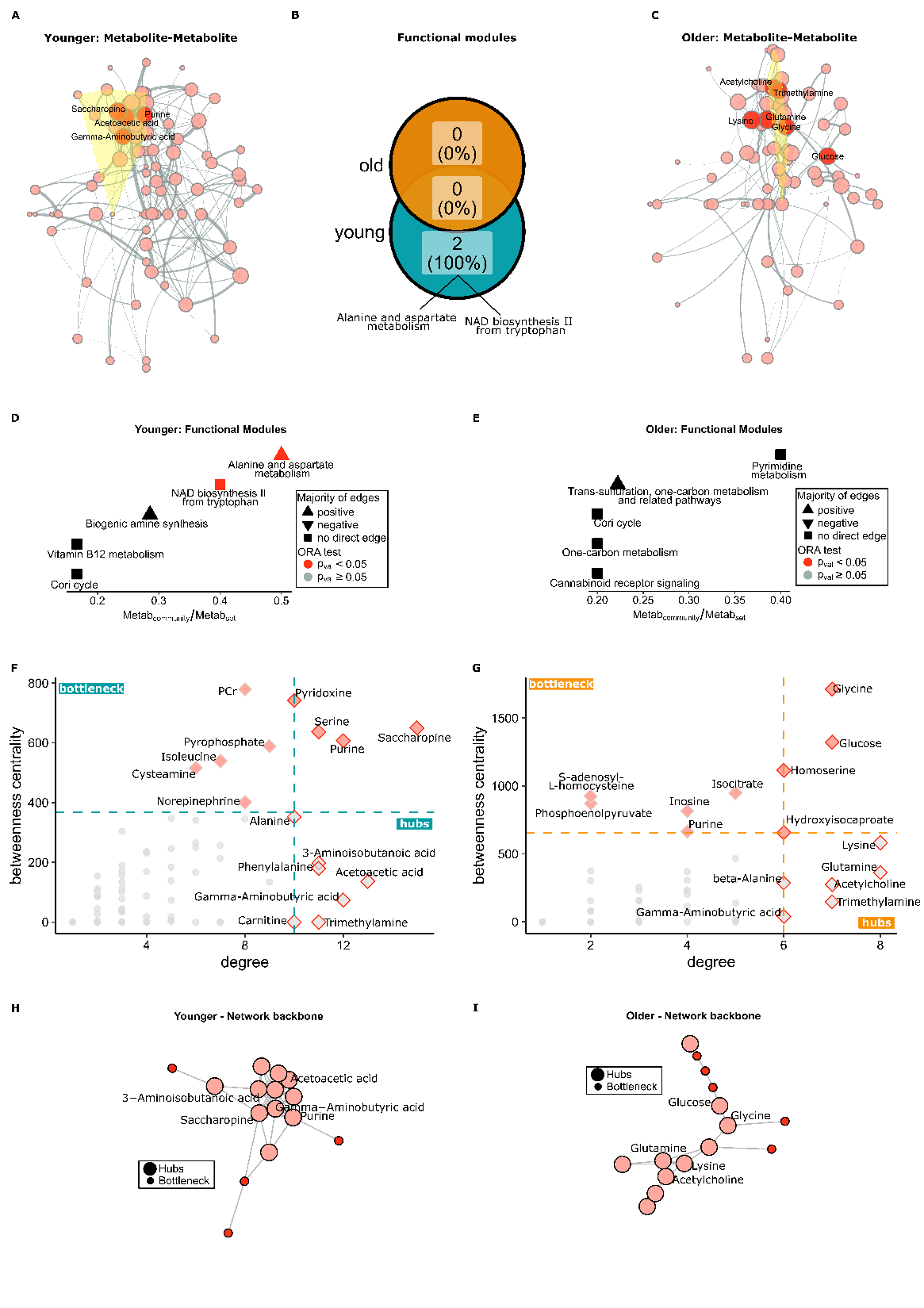
**Figure S7: Metabolite Network analysis on younger and older cohorts.** a) Metabolite network analysis of the younger cohort with largest connected community in yellow. (b) Venn Diagram demonstrating the functional modules identified in each cohort. No modules were found to be enriched in the older cohort. (c) Metabolite network analysis on the older cohort with largest connected community in yellow. (c) Metabolite network analysis on the older cohort. (d) and (e) Overrepresentation analysis (ORA) of the pathways in the largest metabolite community in the younger and older cohorts, respectively. An up/down-pointing triangle indicates most correlations are positive/negative, and a square is used when proteins within a pathway are not directly correlated. (f) and (g) Identification of bottlenecks and hubs making up the overall network analysis in younger and older cohorts, respectively. (h) The backbone of the younger cohort metabolite network. (i) The backbone of the older cohort metabolite network. Hubs are shown as large light blue circles. Bottleneck proteins are shown as smaller dark blue circles.

**
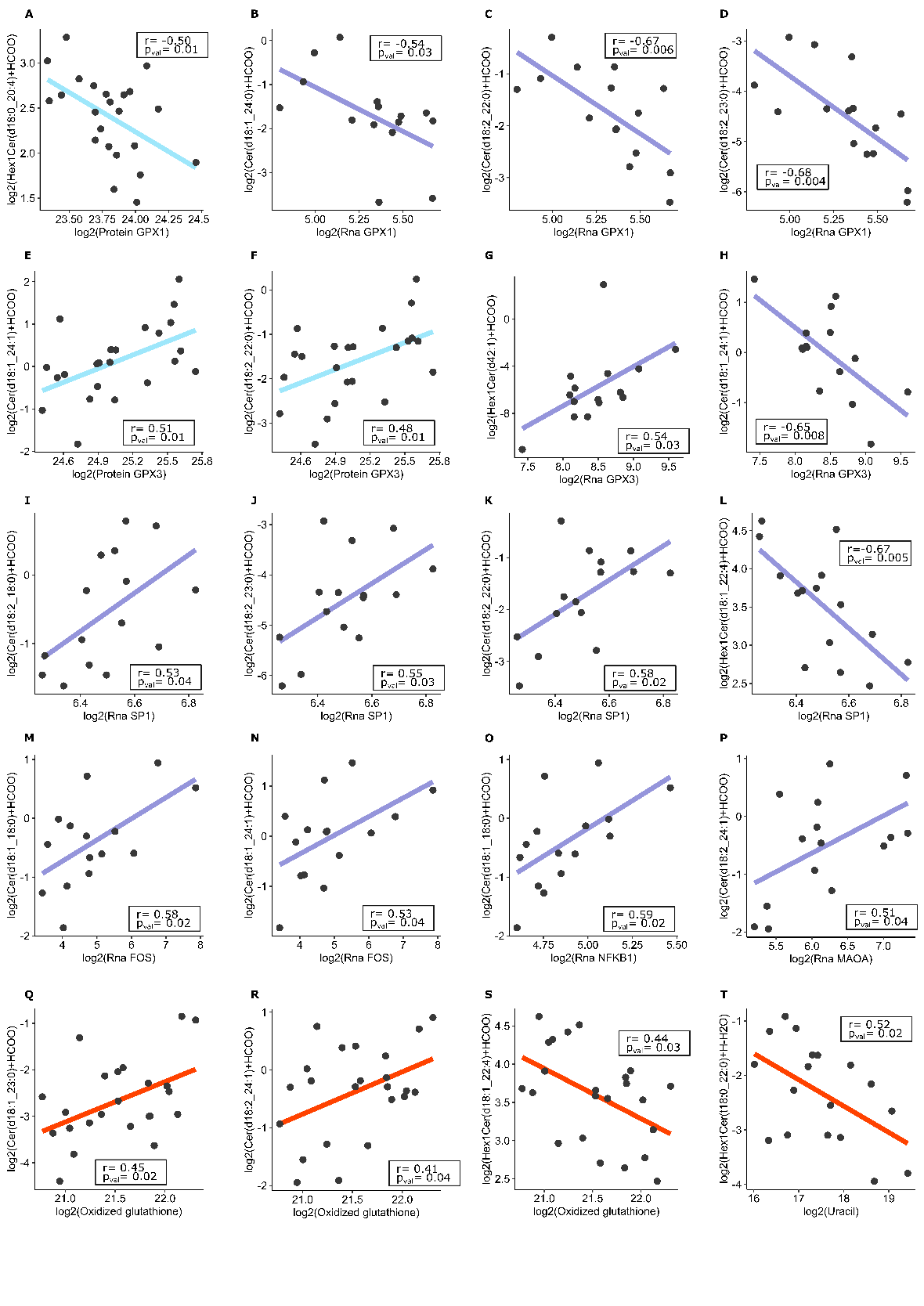
**

**Figure S8 Correlation of ceramides and oxidative stress markers.** The x axis of each graph is the log 2 abundance of the analyte (protein, RNA or metabolite). The y axis is the abundance of a specific ceremide. A blue line represents a correlation with protein. A purple line represents a correlation with RNA. A red line represents a correlation with a metabolite (a) glutathione peroxidase 1 (GPX1) protein correlation a Hexosylceramide. (b) – (d) GPX1 RNA correlation with three different ceramides. (e) and (f) glutathione peroxidase 3 (GPX3) protein correlation with two different ceramides. (g) GPX3 RNA level correlation with a Hexosylceramide. (h) GPX3 RNA level correlation with a ceramide. (i) – (k) transcription factor Sp1 (SP1) RNA correlation with three different ceramides. (l) SP1 correlation with a Hexosylceramide. (m) and (n) C-fos (FOS) RNA correlation with two different ceramides. (o) Nuclear Factor Kappa B Subunit 1 (NFKB1) RNA correlation with a ceramide. (p) Monoamine oxidase A (MAOA) correlation with a ceramide. (q)-(s) Oxidised glutathione correlation with three different ceramides. (t) Uracil correlation with a ceramide.


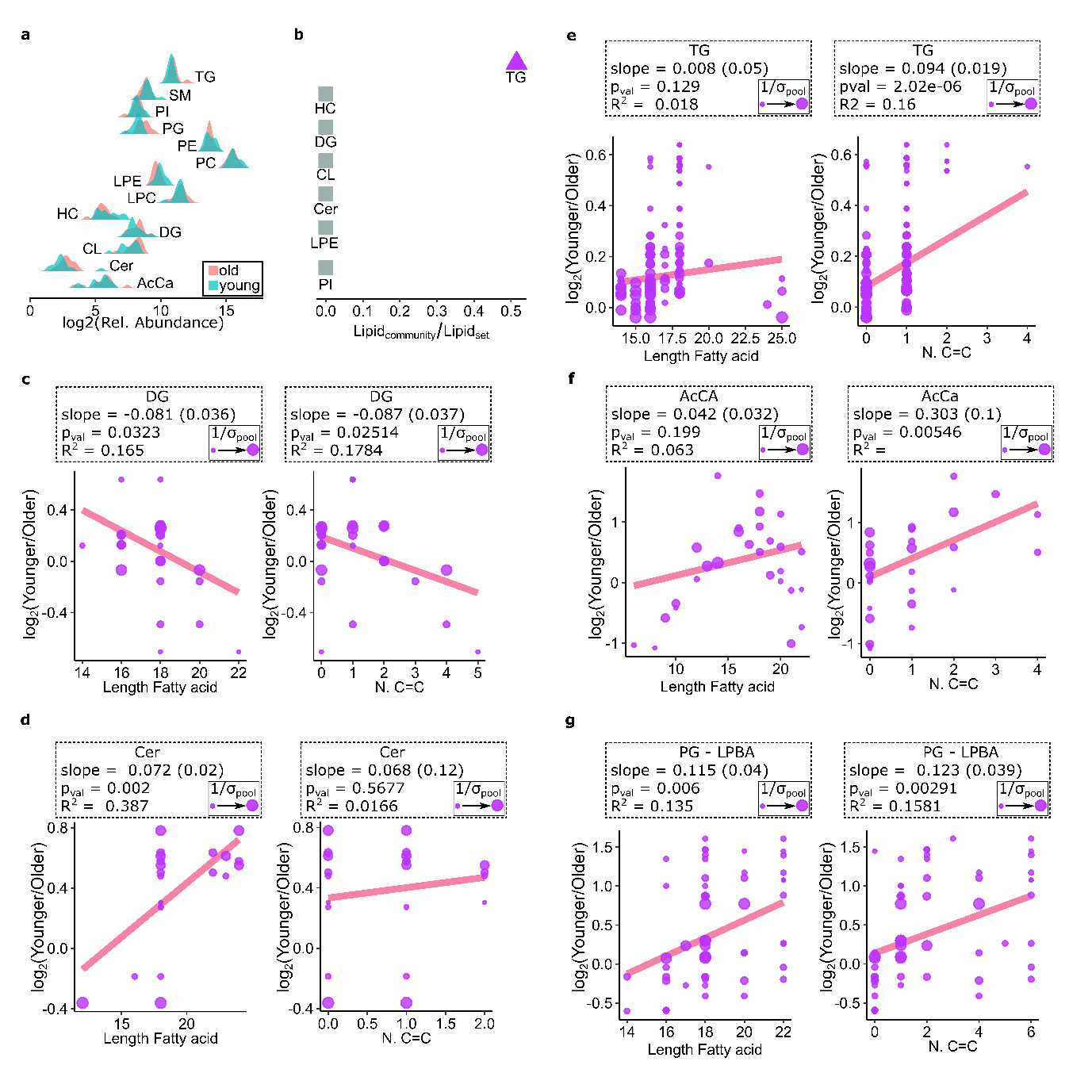


**Figure S9 Analysis of classes of lipids between younger and older hearts.** (a) Distributions of log_2_ abundances of each lipid class for the younger and older cohorts. (b) ORA of pathways in the largest community of lipids. An up/down-pointing triangle indicates most correlations are positive/negative, and a square is used when metabolites within a pathway are not directly correlated. (c) Diacylglycerol (DG) log_2_ fold changes analysed. The trends amongst log_2_ fold changes of DG, length of fatty acids and the number of double bonds are evaluated through the weighted least square. Each log_2_ fold change (dot) is proportional to the inverse of the pooled standard deviation. The summary of each model is reported on the top of the relative plot. (d) Ceramids (Cer) log_2_ fold changes analysed. The trends amongst log_2_ fold changes of Cer, length of fatty acids and the number of double bonds are evaluated through the weighted least square. Each log_2_ fold change (dot) is proportional to the inverse of the pooled standard deviation. The summary of each model is reported on the top of the relative plot. (e) Triglyceride (TG) log_2_ fold changes analysed. The trends amongst log_2_ fold changes of TG, length of fatty acids and the number of double bonds are evaluated through the weighted least square. Each log_2_ fold change (dot) is proportional to the inverse of the pooled standard deviation. The summary of each model is reported on the top of the relative plot. (f) Acylcarnitine (AcCa) log_2_ fold changes analysed. The trends amongst log_2_ fold changes of AcCa, length of fatty acids and the number of double bonds are evaluated through the weighted least square. Each log_2_ fold change (dot) is proportional to the inverse of the pooled standard deviation. The summary of each model is reported on the top of the relative plot. (g) Phosphatidylglycerol-Lysobisphosphatidic acid (PG-LPBA) log_2_ fold changes analysed. The trends amongst log_2_ fold changes of PG-LPBA, length of fatty acids and the number of double bonds are evaluated through the weighted least square. Each log_2_ fold change (dot) is proportional to the inverse of the pooled standard deviation. The summary of each model is reported on the top of the relative plot.


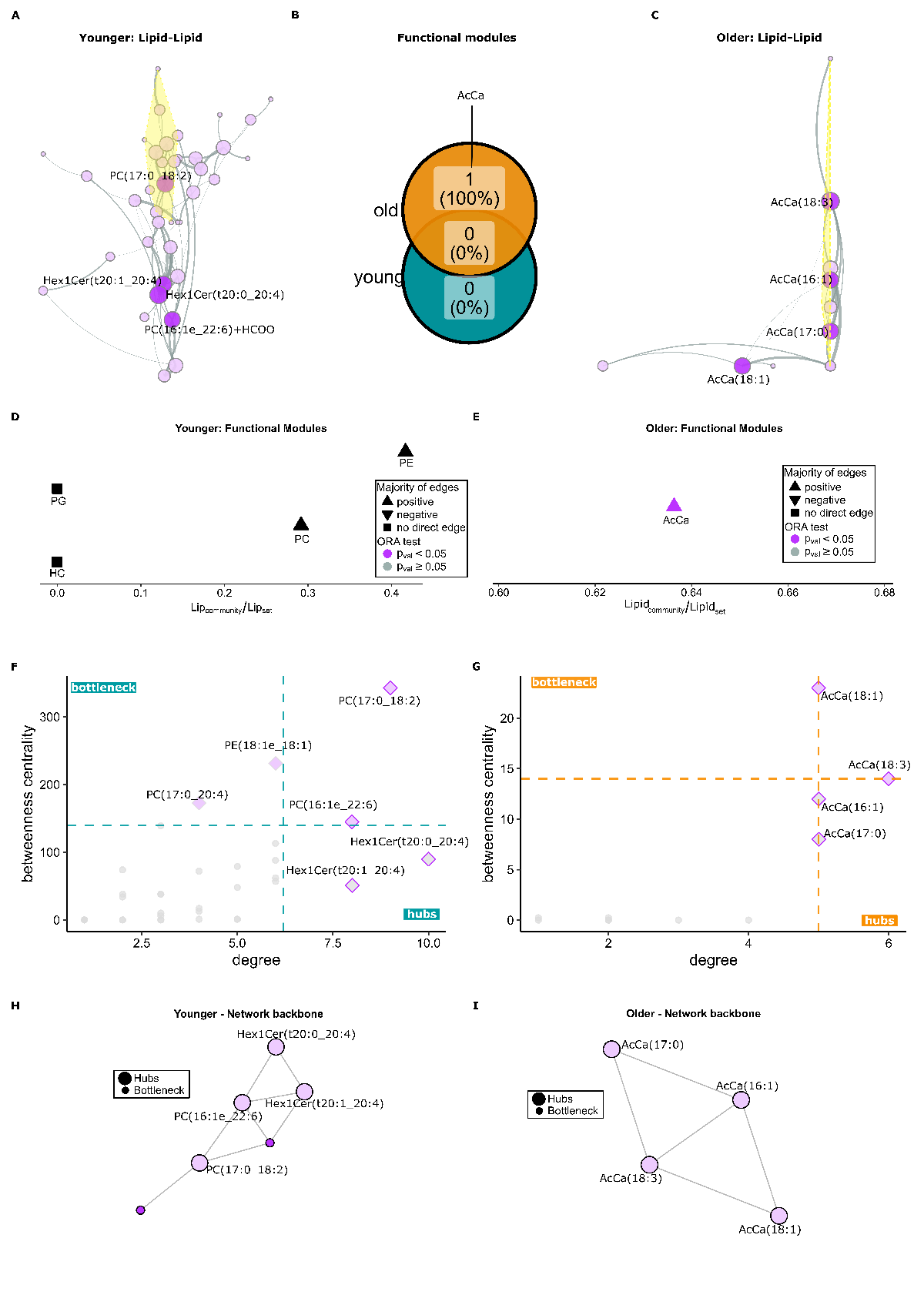
**Figure S10 Lipid Network analysis on younger and older cohorts.** a) Lipid network analysis of the younger cohort with largest connected community in yellow. (b) Venn Diagram demonstrating the functional modules identified in each cohort. No modules were found to be enriched in the younger cohort. (c) Lipid network analysis on the older cohort with largest connected community in yellow. (d) and (e) Overrepresentation analysis (ORA) of the pathways in the largest lipid community in the younger and older cohorts, respectively. An up/down-pointing triangle indicates most correlations are positive/negative, and a square is used when proteins within a pathway are not directly correlated. (f) and (g) Identification of bottlenecks and hubs making up the overall network analysis in younger and older cohorts, respectively. (h) The backbone of the younger cohort lipid network. (i) The backbone of the older cohort lipid network. Hubs are shown as large light blue circles. Bottleneck proteins are shown as smaller dark blue circles.

**
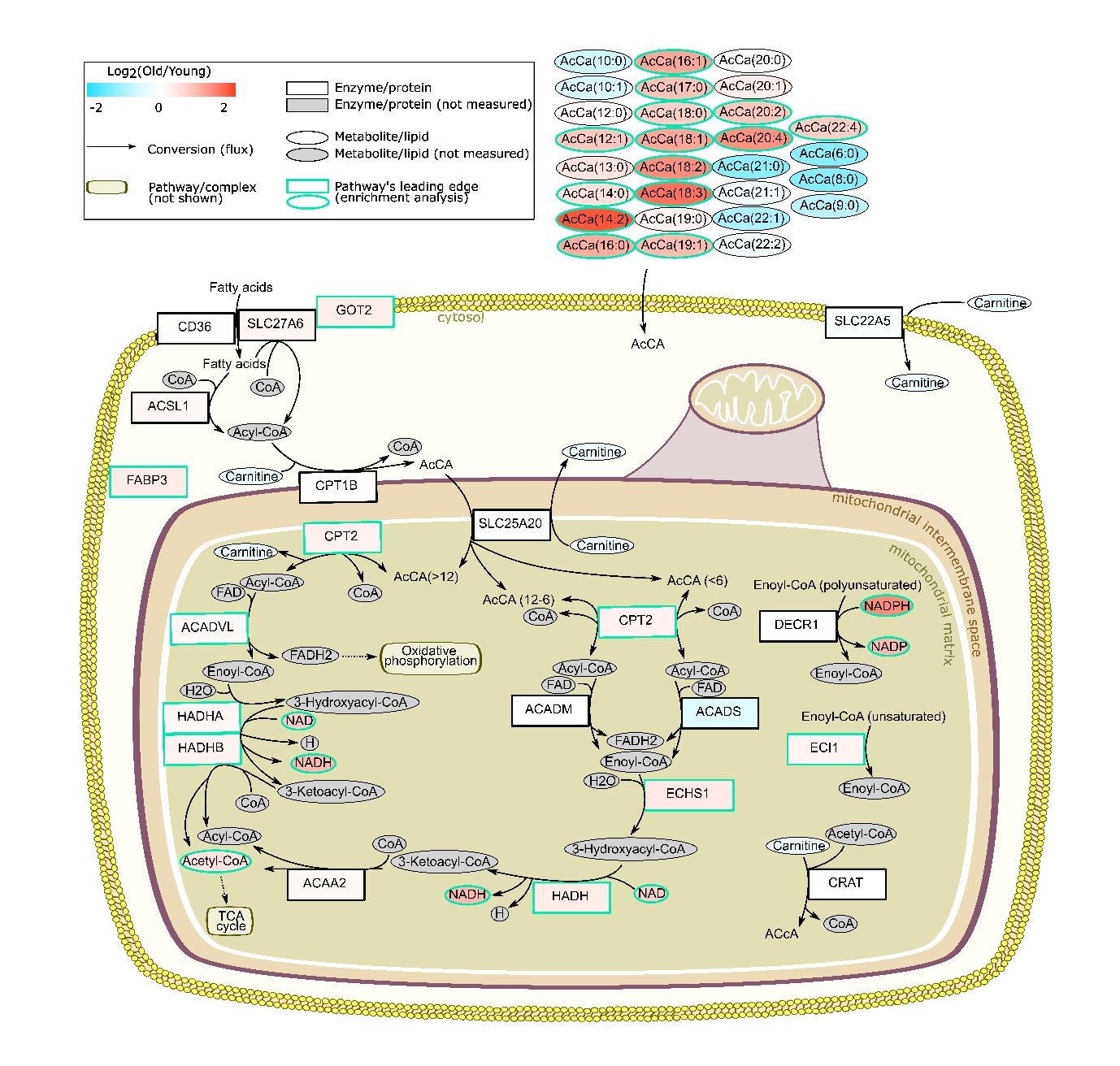
**

**Figure S11 Manually curated beta-oxidation.** The analytes with aquamarine borders are the leading edges of the omics Set Enrichment Analyses (omics SEA). The hypothesis test, dysregulation of the beta-oxidation, leads to *P_val_*  = 0.000584 and Set Size = 54 analytes.
