# Supplementary material for: The Human Cardiac “Age‐OME”: Age‐Specific Changes in Myocardial Molecular Expression
Source: Aging Cell. 2025 Sep 7;24(11):e70219. doi: 10.1111/acel.70219 (PMC12610413; doi:10.1111/acel.70219)
Supplement: Supplementary file 1 — Data S1. acel70219‐sup‐0001‐DataS1.zip. [file ACEL-24-e70219-s011.zip › Supplementary Tables S0-S13 Captions.docx]

**Supplementary Table Captions**

**Data**

**File Name: Table S0**

**Description**: The log transformed, normalised values of all samples for proteomics, metabolomics, lipidomics and transcriptomics as well as data with all imputed values for each omics set. Additionally, the overlap of samples between each omics analysis is also presented.

**File Name: Table S1**

**Description**: The clinical information for each donor sample used in the study

**File Name: Table S2**

**Description:** Differential expression analysis results of younger vs older hearts for proteomics, metabolomics, lipidomics and transcriptomics. Each analyte's abundance difference was tested using the two-sided LIMMA t-test on the normalised and log_2_ transformed data.

**File Name: Table S3**

**Description:** Differential expression analysis of proteins between younger males vs older males and

younger female’s vs older females.

**File Name: Table S4**

**Description:** Effect size comparison and related p-values for the whole cohort, and gender stratified cohorts

**File Name: Table S5**

**Description:** Weighted Correlation Network Analysis (WCNA) results and over-representation analysis (ORA) results at the protein level. The ORA was performed on the most populated community presented in the WCNA.

**File Name: Table S6**

**Description:** Weighted Correlation Network Analysis (WCNA) results and over-representation analysis (ORA) results at the metabolite level. The ORA was performed on the most populated community presented in the WCNA.

**File Name: Table S7**

**Description:** Set enrichment analysis results of younger vs older hearts at the lipid level.

**File Name: Table S8**

**Description:** Weighted Correlation Network Analysis (WCNA) results and over-representation analysis (ORA) results at the lipid level. The ORA was performed on the most populated community presented in the WCNA.

**File Name: Table S9**

**Description:** Weighted Correlation Network Analysis (WCNA) results and over-representation analysis (ORA) results at the transcript level. The ORA was performed on the most populated community presented in the WCNA.

**File Name: Table S10**

**Description:** Set enrichment analysis results of old vs young hearts at the protein, metabolite and transcript level. The analysis was performed on the WikiPathways database.

**File Name: Table S11**

**Description:** Results of network analyses done separately on the younger and older cohorts for the protein, metabolite, lipid and transcript level as well the identification of significant functional modules in each omics set.

**File Name: Table S12**

**Description:** Correlation results between key differentially expressed proteins and their corresponding transcript levels.

**File Name: Table S13**

**Description:** Results of correlation analysis between ceramide levels and oxidative stress markers measured in the proteomics, metabolomics and transcriptomics datasets.
